# Comparison of Non-human Primate versus Human Induced Pluripotent Stem Cell-Derived Cardiomyocytes for Treatment of Myocardial Infarction

**DOI:** 10.1016/j.stemcr.2018.01.002

**Published:** 2018-02-01

**Authors:** Xin Zhao, Haodong Chen, Dan Xiao, Huaxiao Yang, Ilanit Itzhaki, Xulei Qin, Tony Chour, Aitor Aguirre, Kim Lehmann, Youngkyun Kim, Praveen Shukla, Alexandra Holmström, Joe Z. Zhang, Yan Zhuge, Babacar C. Ndoye, Mingtao Zhao, Evgenios Neofytou, Wolfram-Hubertus Zimmermann, Mohit Jain, Joseph C. Wu

**Affiliations:** 1 Stanford Cardiovascular Institute, Stanford, CA 94305-5454, USA; 2 Institute for Stem Cell Biology and Regenerative Medicine, Stanford, CA 94305, USA; 3 Departments of Medicine and Pharmacology, University of California, San Diego, CA 92093, USA; 4 Institute of Pharmacology and Toxicology, University Medical Center Goettingen, 37075 Goettingen, Germany; 5 DZHK (German Center for Cardiovascular Research), Partner Site, Goettingen, Germany; 6 Department of Medicine, Division of Cardiology, Stanford University School of Medicine, Stanford, CA 94305, USA

**Keywords:** iPSC-CM, non-human primate, RNA-seq, metabolomics, myocardial infarction

## Abstract

Non-human primates (NHPs) can serve as a human-like model to study cell therapy using induced pluripotent stem cell-derived cardiomyocytes (iPSC-CMs). However, whether the efficacy of NHP and human iPSC-CMs is mechanistically similar remains unknown. To examine this, RNU rats received intramyocardial injection of 1 × 10^7^ NHP or human iPSC-CMs or the same number of respective fibroblasts or PBS control (n = 9–14/group) at 4 days after 60-min coronary artery occlusion-reperfusion. Cardiac function and left ventricular remodeling were similarly improved in both iPSC-CM-treated groups. To mimic the ischemic environment in the infarcted heart, both cultured NHP and human iPSC-CMs underwent 24-hr hypoxia *in vitro*. Both cells and media were collected, and similarities in transcriptomic as well as metabolomic profiles were noted between both groups. In conclusion, both NHP and human iPSC-CMs confer similar cardioprotection in a rodent myocardial infarction model through relatively similar mechanisms via promotion of cell survival, angiogenesis, and inhibition of hypertrophy and fibrosis.

## Highlights

• Cardiomyocytes from both non-human primate and human iPSCs improve cardiac function
• NHP and human iPSC-CMs similarly attenuate left ventricular remodeling
• Transcriptomic and metabolomic profiles of both iPSC-CMs were similar under hypoxia

## Teaser

In this article, Wu and colleagues demonstrate the therapeutic similarities of non-human primate iPSC-CMs and human iPSC-CMs to treat myocardial infarction by improving cardiac function and attenuating myocardial remodeling in a rodent myocardial infarction model. Mechanisms of iPSC-CM therapy include promotion of cell survival, angiogenesis, and inhibition of hypertrophy and fibrosis.

## Introduction

With the recent progress in effective preventions and advanced treatments, mortality rates for heart failure and acute myocardial infarction (MI) have declined. However, 1 in every 4 deaths is still caused by heart disease in the United States (Vos et al., 2012). Despite early pharmacologic and medical device intervention to reduce myocardial loss after infarction, viable cardiomyocytes (CMs) within the ischemic zone and adjacent areas still experience increased workload and metabolic stress, resulting in heart failure with further cell loss and pathologic myocardial remodeling (Pfeffer and Braunwald, 1990).

In recent years, stem cell-based regenerative therapy has entered preclinical and clinical trials to repair the infarcted heart (Laflamme et al., 2007, Mangi et al., 2003, Menasché et al., 2015, Menasché et al., 2018). The unique properties of induced pluripotent stem cells (iPSCs), including their ability for self-renewal, provide a sufficient resource to generate differentiated cells for transplantation. Although preclinical studies have shown that CMs differentiated from iPSCs can improve cardiac function and attenuate myocardial remodeling after MI (Carpenter et al., 2012, Citro et al., 2014), the field is still mainly at the preclinical trial stage due to technical and practical reasons (Neofytou et al., 2015). It is important to examine the safety and immunogenicity further in a human-like animal model, such as non-human primates (NHPs). NHPs have phylogenetic proximity to humans and can be an ideal model for preclinical autologous/allogeneic transplantation studies. However, an essential step before NHP trials is to prove the equivalence of functional efficacies and underlying therapeutic mechanisms between NHP iPSC-CMs and human iPSC-CMs. This step is necessary to translate NHP animal results to clinical studies.

In the present study, we injected NHP iPSC-CMs or human iPSC-CMs intramyocardially in a subacute MI rat model. Four weeks after cell injection, similar improvements in left ventricular (LV) function and myocardial remodeling were noted in both cell-treated groups, but not in the control groups treated with PBS or NHP fibroblasts or human fibroblasts. We further examined the effects of oxygen depletion on NHP iPSC-CMs and human iPSC-CMs *in vitro*, which is a condition the cells first encounter after injection into the heart. The majority of the changes in transcriptomic and metabolomic profile of NHP and human iPSC-CMs were similar, with some species-specific differences.

## Results

### CM Differentiation from NHP iPSCs and Human iPSCs

Thirty days after differentiation, silencing of reprogramming factors were confirmed by qPCR in both NHP and human iPSC-CMs (data not shown). Immunohistological staining showed that both NHP and human iPSC-CMs expressed key cardiac markers such as cardiac troponin T (cTnT) and α-actinin. In addition, the sarcomere structure was clear and well aligned in both NHP and human iPSC-CMs (Figure S1A), confirming successful differentiation. Both NHP and human iPSC-CMs showed cardiac-specific electrophysiological phenotype by displaying the three subtypes of cardiac action potential: ventricular-like, atrial-like, and nodal-like (Figure S1B and Table S1).

### Left Ventricular Function Change after Cell Injection

**Figure 1:**
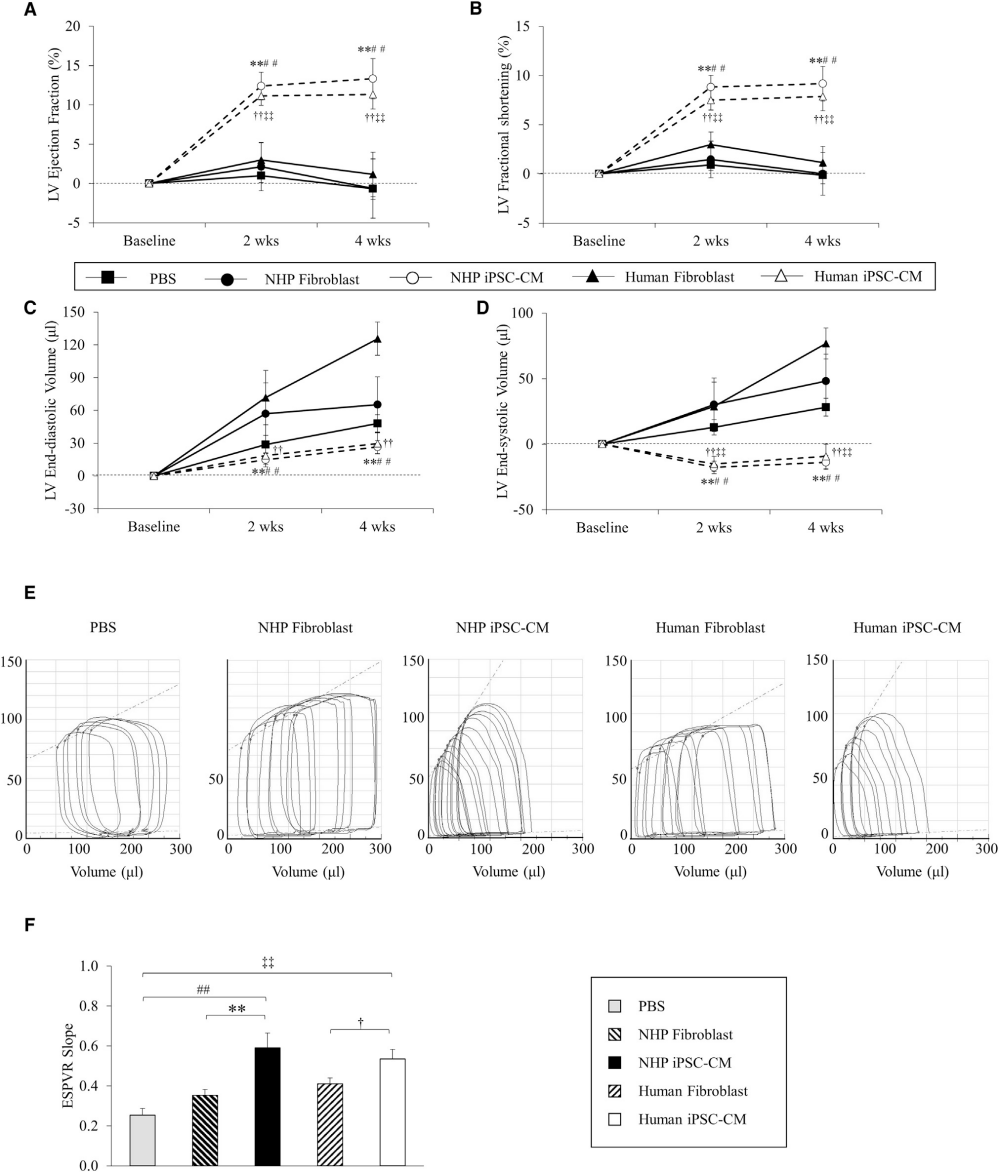
Cardiac Function Assessment after Cell Injection Echocardiography was monitored at 1 day before cell injection, and 2 weeks and 4 weeks after cell injection. (A and B) LV ejection fraction (A) and fractional shortening (B) were improved significantly in both NHP and human iPSC-CM groups compared with the PBS group. (C) LV end-diastolic volume was markedly dilated in the PBS control, NHP fibroblast, and human fibroblast groups compared with both NHP and human iPSC-CM groups. (D) LV end-systolic volume was increased in the PBS control, NHP fibroblast, and human fibroblast groups but decreased in both NHP and human iPSC-CM groups (n = 14 independent experiments). Hemodynamics was analyzed using pressure-volume Millar catheter. (E) Examples of changes in pressure-volume relationships during inferior vena cava occlusion. (F) Slope of end-systolic pressure-volume relationship (ESPVR) showed significantly preserved cardiac contractility in both NHP and human iPSC-CM groups. PBS, n = 7; NHP fibroblast, n = 9; NHP iPSC-CM, n = 11; human fibroblast, n = 9; human iPSC-CM, n = 12 independent experiments. Data are presented as mean ± SEM. ^∗∗^p < 0.01, NHP iPSC-CM versus NHP fibroblast; ^##^p < 0.01, NHP iPSC-CM versus PBS; ^†^p < 0.05, ^††^p < 0.01, human iPSC-CM versus human fibroblast; ^‡‡^p < 0.01, human iPSC-CM versus PBS; by one-way ANOVA.

One day before cell injection, post-MI cardiac function was assessed by echocardiography (Table S2). The rats were divided into five groups to receive PBS, NHP fibroblasts, NHP iPSC-CMs, human fibroblasts, or human iPSC-CMs. Four weeks after injection, the left ventricular ejection fraction (LVEF) did not change significantly in the PBS group (−0.7% ± 1.4%) or fibroblast control groups (−0.6% ± 3.8% in NHP fibroblast group; +1.2% ± 2.8% in human fibroblast group) but was increased significantly in both the NHP iPSC-CM group (+13.3% ± 1.9%) and the human iPSC-CM group (+11.3% ± 2.6%) ( p < 0.01, Figure 1A). Similarly, fractional shortening was also significantly improved in both cell-treated groups (+9.2% ± 1.5% in NHP iPSC-CM group and +7.9% ± 1.8% in human iPSC-CM group versus −0.1% ± 0.9% in PBS group, 0.0% ± 2.2% in NHP fibroblast group, and +1.2% ± 1.6% in human fibroblast group; p < 0.01, Figure 1B). The improved contractility was accompanied by the amelioration of LV chamber dilatation at both phases of end-diastole (+27 ± 5.9 μL in NHP iPSC-CM group and +24 ± 7.9 μL in human iPSC-CM group versus +48 ± 7.9 μL in PBS group, +65 ± 25 μL in NHP fibroblast group, and +126 ± 15 μL in human fibroblast group; p < 0.05, Figure 1C) and end-systole (−14 ± 5.4 μL in NHP iPSC-CM group and −9.3 ± 9.4 μL in human iPSC-CM group versus +28 ± 6.7 μL in PBS group, +48 ± 21 μL in NHP fibroblast group, and +77 ± 12 μL in human fibroblast group; p < 0.05, Figure 1D) compared with the baseline.

Hemodynamic analysis further confirmed the improvement of LV systolic and diastolic function in both NHP and human iPSC-CM-treated groups. With a similar heart rate, LV systolic pressure, and LV systemic pressure, we found that the maximum dP/dt was significantly higher in both NHP iPSC-CM (7,497 ± 316 mmHg/s, n = 11) and human iPSC-CM groups (7,991 ± 434 mmHg/s, n = 12) compared with the NHP fibroblast (5,835 ± 242 mmHg/s, n = 9), human fibroblast (6,385 ± 259 mmHg/s, n = 9), and PBS control groups (6,628 ± 103 mmHg/s, n = 7; p < 0.05) (Table S3). The slope of end-systolic pressure-volume relationship generated by inferior vena cava occlusion suggests a similarly increased contractility in both NHP iPSC-CM and human iPSC-CM groups compared with both NHP and human fibroblast-treated groups as well as the PBS-treated group (p < 0.01, Figures 1E and 1F). The LV minimum dP/dt was lower in both NHP iPSC-CM (−6,393 ± 385 mmHg/s) and human iPSC-CM groups (−6,740 ± 499 mmHg/s) compared with NHP fibroblast (−5,357 ± 333 mmHg/s) and human fibroblast groups (−5,633 ± 196 mmHg/s), as well as the PBS group (−5,327 ± 101 mmHg) (p < 0.05, Table S3). The end-diastolic pressure and time constant, tau, in both cell-treated groups were similar and were significantly decreased compared with both fibroblast groups or PBS group (p < 0.05, Table S3).

### Reduced Infarct Size and Increased Viable Myocardium in Cell-Treated Groups

**Figure 2:**
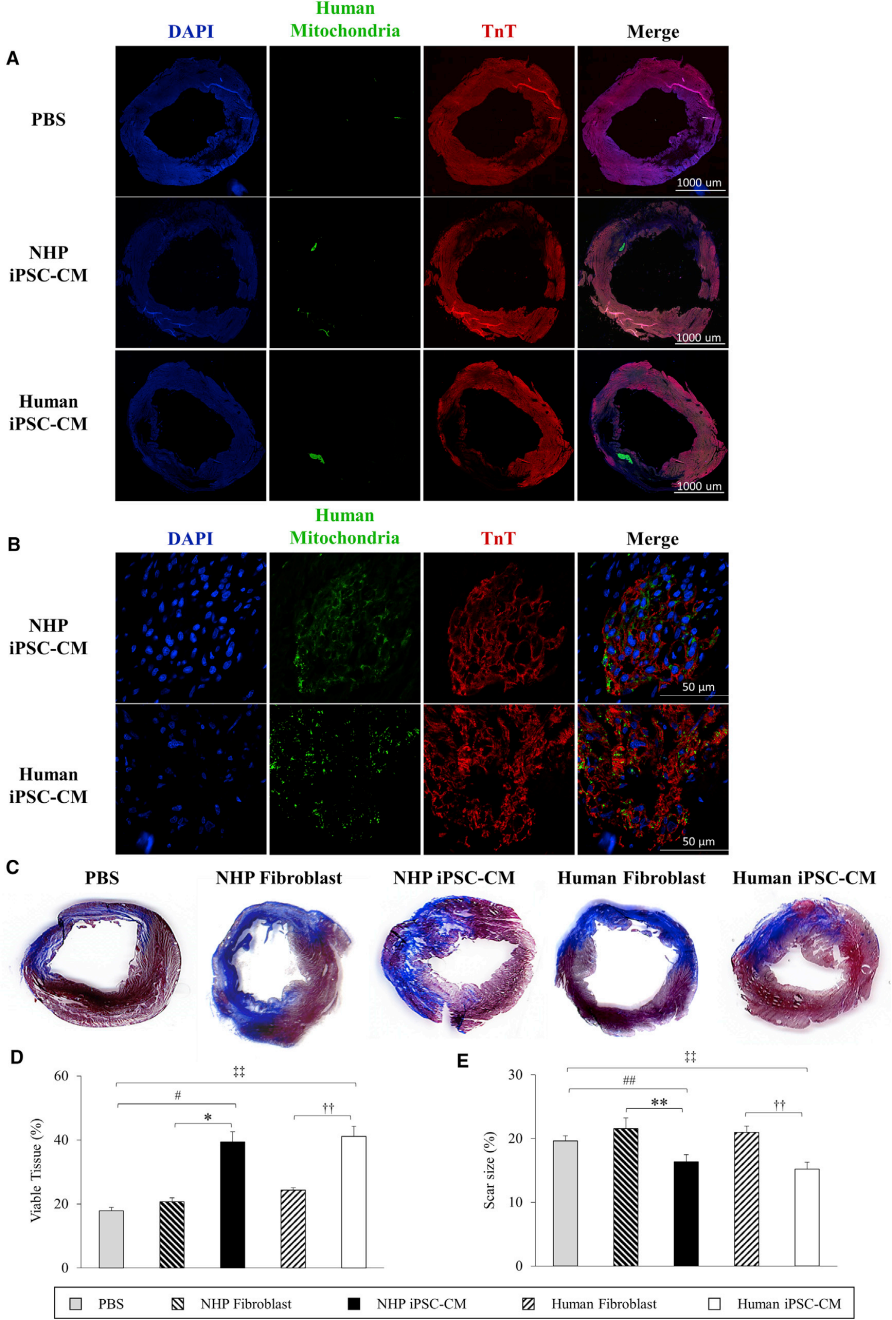
Graft and Scar Size Assessment at 4 Weeks after Myocardial Infarction (A) Examples of viable graft in the ischemic zone. Both NHP and human iPSC-CMs were identified with non-specific cardiac troponin T and human specific mitochondria at 4× magnification. (B) Viable grafts in both groups of iPSC-CM-treated hearts were observed under 63× magnification. (C) Representative pictures of infarcted left ventricle with trichrome staining. PBS and fibroblast control groups showed more transmural infarction than iPSC-CM-treated groups, which had more viable myocardium. (D) The amount of viable myocardium was quantified within the ischemic zone and presented as a percentage of the area of ischemic zone. (E) Scar size, presented as a percentage of fibrotic tissue to whole myocardial area, was compared among PBS and cell-treated groups. PBS, n = 5; NHP fibroblast, n = 6; NHP iPSC-CM, n = 6; human fibroblast, n = 6; human iPSC-CM, n = 6 independent experiments. Data are presented as mean ± SEM. ^∗^p < 0.05, ^∗∗^p < 0.01, NHP iPSC-CM versus NHP fibroblast; ^#^p < 0.05, ^##^p < 0.01, NHP iPSC-CM versus PBS; ^††^p < 0.01, human iPSC-CM versus human fibroblast; ^‡‡^p < 0.01, human iPSC-CM versus PBS.

Four weeks after cell transplantation, graft survival was identified in the ischemic zone at different sections of the left ventricle in both NHP and human iPSC-CM groups as assessed by cTnT and human specific mitochondria double staining (Figures 2A and 2B). The graft resulted in an increased amount of viable tissue within the infarct zone in both NHP iPSC-CM (39% ± 3.2%) and human iPSC-CM groups (41% ± 3.2%) compared with the NHP fibroblast (21% ± 1.2%) and human fibroblast groups (24% ± 0.7%), as well as the PBS group (18% ± 1.1%; p < 0.05) (Figures 2C and 2D). Additionally, the infarct size was significantly smaller in the groups treated with NHP iPSC-CMs (16% ± 1.1%) and human iPSC-CMs (15% ± 1.1%) than that treated with NHP fibroblast (22% ± 1.7%), human fibroblast (21% ± 1.7%), and PBS (20% ± 0.8%; p < 0.05) (Figure 2E).

### Attenuated Myocardial Remodeling with Increased Angiogenesis after CM Transplantation

**Figure 3:**
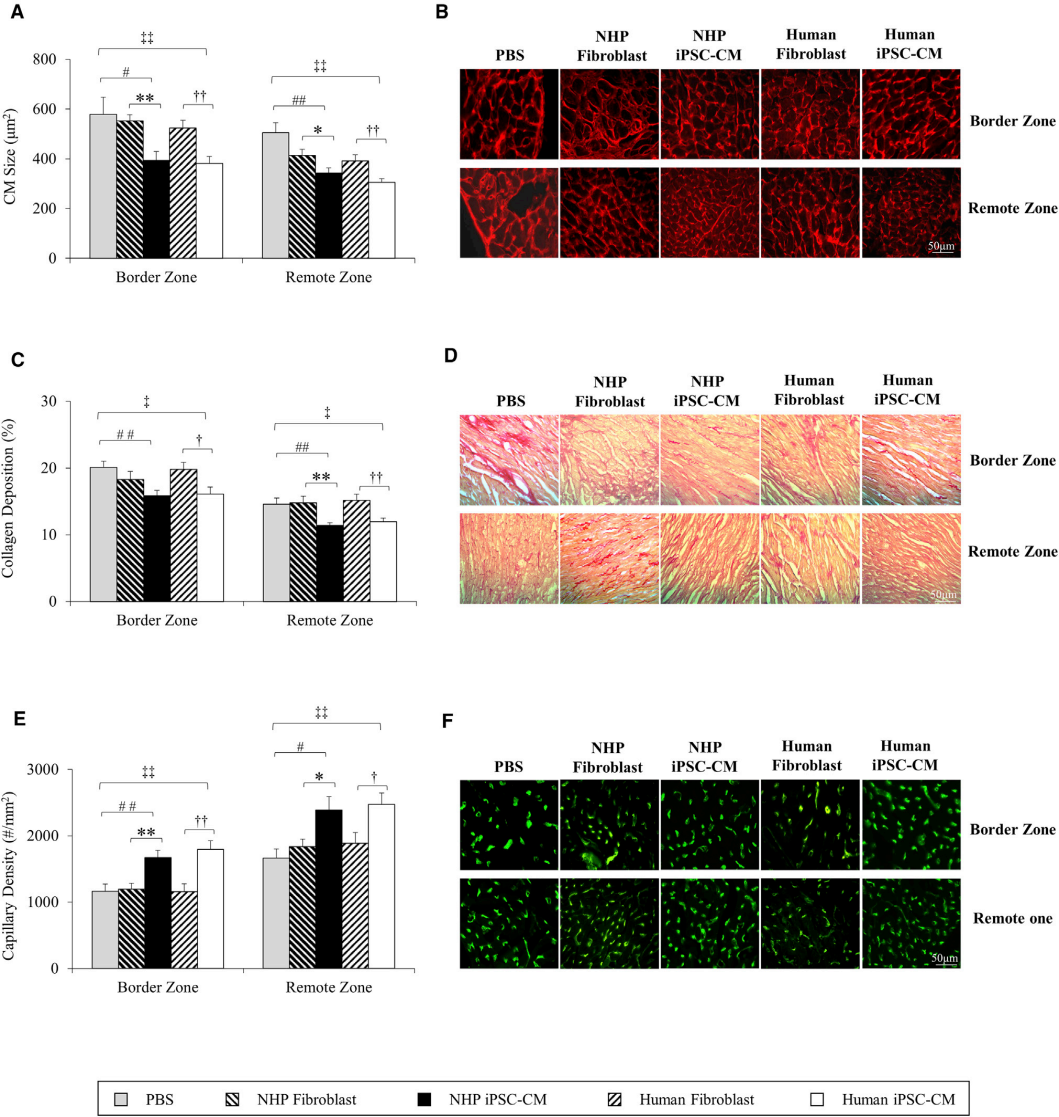
Myocardial Remodeling Was Compared at Histological Level at Border Zone and Remote Zone to the Infarct (A) Cardiomyocyte size was compared among PBS, NHP fibroblast, NHP iPSC-CM, human fibroblast, and human iPSC-CM groups. (B) Examples of wheat germ agglutinin staining showing the size of endogenous mouse cardiomyocytes of the three groups. (C) Fibrosis was quantified as the percentage of interstitial collagen deposition. (D) Representative images of Picro-Sirius red staining showing interstitial fibrosis of the three groups. (E) Capillary density was quantified as absolute number of capillaries per unit area. (F) Representative CD144 staining showing capillaries. PBS, n = 5; NHP fibroblast, n = 6; NHP iPSC-CM, n = 6; human fibroblast, n = 6; human iPSC-CM, n = 6 independent experiments. Data are presented as mean ± SEM. ^∗^p < 0.05, ^∗∗^p < 0.01, NHP iPSC-CM versus NHP fibroblast; ^#^p < 0.05, ^##^p < 0.01, NHP iPSC-CM versus PBS; ^†^p < 0.05, ^††^p < 0.01, human iPSC-CM versus human fibroblast; ^‡^p < 0.05, ^‡‡^p < 0.01 human iPSC-CM versus PBS.

With the improvement in LV function and more viable tissue in cell-treated groups, the extent of myocardial remodeling was further assessed at both the border zone (BZ) and remote zone (RZ) of the infarct site according to the histology approach. The endogenous rat cardiomyocytes were significantly smaller in the groups treated with NHP iPSC-CMs (393 ± 36 μm^2^ at BZ and 343 ± 21 μm^2^ at RZ) or human iPSC-CMs (381 ± 29 μm^2^ at BZ and 343 ± 21 μm^2^ at RZ) compared with PBS group (579 ± 69 μm^2^ at BZ and 505 ± 40 μm^2^ at RZ), NHP fibroblast group (552 ± 25 μm^2^ at BZ and 413 ± 25 μm^2^ at RZ), and human fibroblast group (524 ± 31 μm^2^ at BZ and 391 ± 24 μm^2^ at RZ), suggesting a more limited hypertrophy (p < 0.05, Figures 3A and 3B). A similar reduction of cardiac fibrosis was noted in both cell-treated groups (16% ± 0.8% at BZ and 11% ± 0.4% at RZ in the NHP iPSC-CM group, 16% ± 1.0% at BZ and 12% ± 0.5% at RZ in the human iPSC-CM group), but not in control groups (20% ± 0.9% at BZ and 15% ± 0.9% at RZ in PBS group, 18% ± 1.2% at BZ and 15% ± 1.0% at RZ in NHP fibroblast group, 20% ± 1.1% at BZ and 15% ± 0.9% at RZ in human fibroblast group) (p < 0.05; Figures 3C and 3D). In addition to the attenuation of the myocardial remodeling, the capillary density in both NHP iPSC-CM (1,672 ± 110/mm^2^ at BZ and 2,387 ± 205/mm^2^ at RZ) and human iPSC-CM groups (1,795 ± 130/mm^2^ at BZ and 2,475 ± 170/mm^2^ at RZ) were significantly higher than those in the NHP fibroblast group (1,199 ± 88/mm^2^ at BZ and 1,840 ± 109/mm^2^ at RZ), human fibroblast group (1,158 ± 120/mm^2^ at BZ and 1,889 ± 161/mm^2^ at RZ), and PBS group (1,163 ± 109/mm^2^ at BZ and 1,665 ± 136/mm^2^ at RZ) (p < 0.01; Figures 3E and 3F), indicating a possible increase in angiogenesis and a reduction in cellular remodeling/hypertrophy.

### Gene Profile Change in Response to Oxygen Depletion *In Vitro*

**Figure 4:**
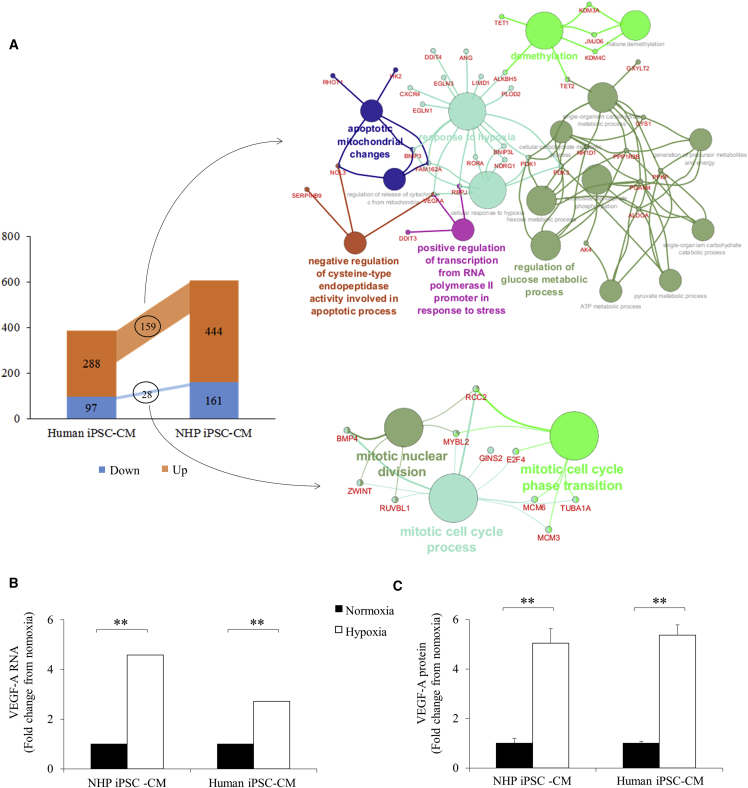
RNA-Seq Analysis of Both NHP and Human iPSC-CMs in Response to 24-hr Oxygen Depletion (A) The numbers of significantly regulated genes (p < 0.05) and the significant biological function of the common genes in both groups are listed. (B) Fold change of VEGF gene in hypoxic iPSC-CMs from both species. (C) Fold change of VEGF in culture medium using proteomic angiogenesis assay. n = 3 independent experiments. Data are presented as mean ± SEM. ^∗∗^p < 0.01.

**Figure 5:**
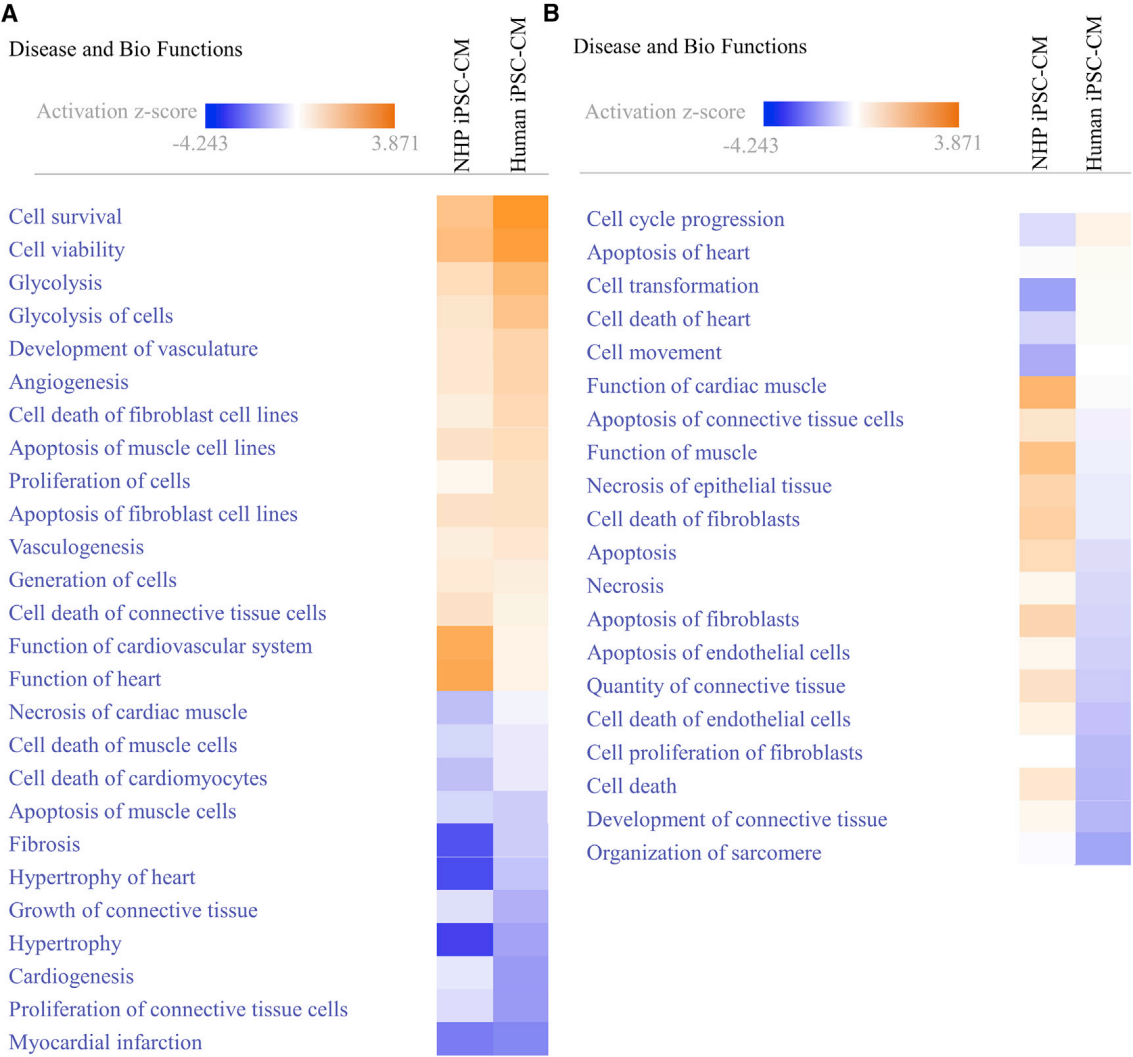
Comparison of Biological Function Revealed by RNA-Seq Analysis between NHP and Human iPSC-CMs after 24-hr Hypoxia (A) Common biological functions of the genes. (B) Biological functions that are regulated toward opposite directions. n = 3 independent experiments.

When transplanted, exogenous cells initially encounter the hypoxic environment of the ischemic heart. To study their responses to hypoxic condition, we next subjected cultured NHP iPSC-CMs and human iPSC-CMs to oxygen depletion for 24 hr. RNA sequencing (RNA-seq) revealed a total of 444 genes upregulated and 161 genes downregulated in NHP iPSC-CMs compared with 288 genes upregulated and 97 genes downregulated in human iPSC-CMs. Among these genes, only 159 were commonly upregulated and 28 commonly downregulated in the two groups (Table S4). Further analysis indicates that these genes are responsible for regulation of glucose metabolism and post-translational modification, among other processes (Figure 4A). However, when evaluating the functions of differentially expressed genes in the two species, more common pathways were identified with the functions of promoting cell viability, cell survival, and glycolysis, as well as in inhibiting cardiac hypertrophy and fibrosis (Figure 5A). Interestingly, some pathways that are regulated toward opposite directions between the two groups are more related to the fate of connective tissue cells, fibroblasts, and endothelial cells (Figure 5B).

Using Ingenuity Pathway Analysis (IPA), genes involved in shared pathways regulating cell survival, angiogenesis (Figures S2A and S2B), hypertrophy, and fibrosis (Figures S3A and S3B) are dimensionally divided into nucleus, cytosol, membrane, and extracellular space according to the spatial distribution of their encoded proteins. Interestingly, the vascular endothelial growth factor (VEGF) was significantly elevated in both groups (4.6-fold increase in NHP iPSC-CMs and 2.7-fold in human iPSC-CMs; p < 0.01, Figure 4B) and was noted in all four protective pathways. More importantly, this protein was located in the extracellular space according to IPA as a secreted factor. The media collected from the cultured CMs further proved that VEGF was indeed secreted from both types of iPSC-CMs after oxygen depletion (5.0 ± 0.6-fold increase in NHP iPSC-CMs and 5.4 ± 0.4-fold in human iPSC-CMs; p < 0.01, Figure 4C).

To understand how these differentially expressed genes were regulated, we next studied the transcription factors that bind to the conserved regions of these genes using the online Database for Annotation, Visualization and Integrated Discovery (DAVID) v6.8. We found that among the top enriched transcription factors, 25 of them, including hepatic leukemia factor and insulin-like growth factor 1 (IGF-1), were shared by both species. Other transcription factors distinctly appeared in one of the groups, such as FOXO1 and STAT1 in NHP iPSC-CMs versus FOXO3 and ATF6 in human iPSC-CMs (Table S5).

### Metabolic Paracrine Actions from iPSC-CMs after Oxygen Depletion

**Figure 6:**
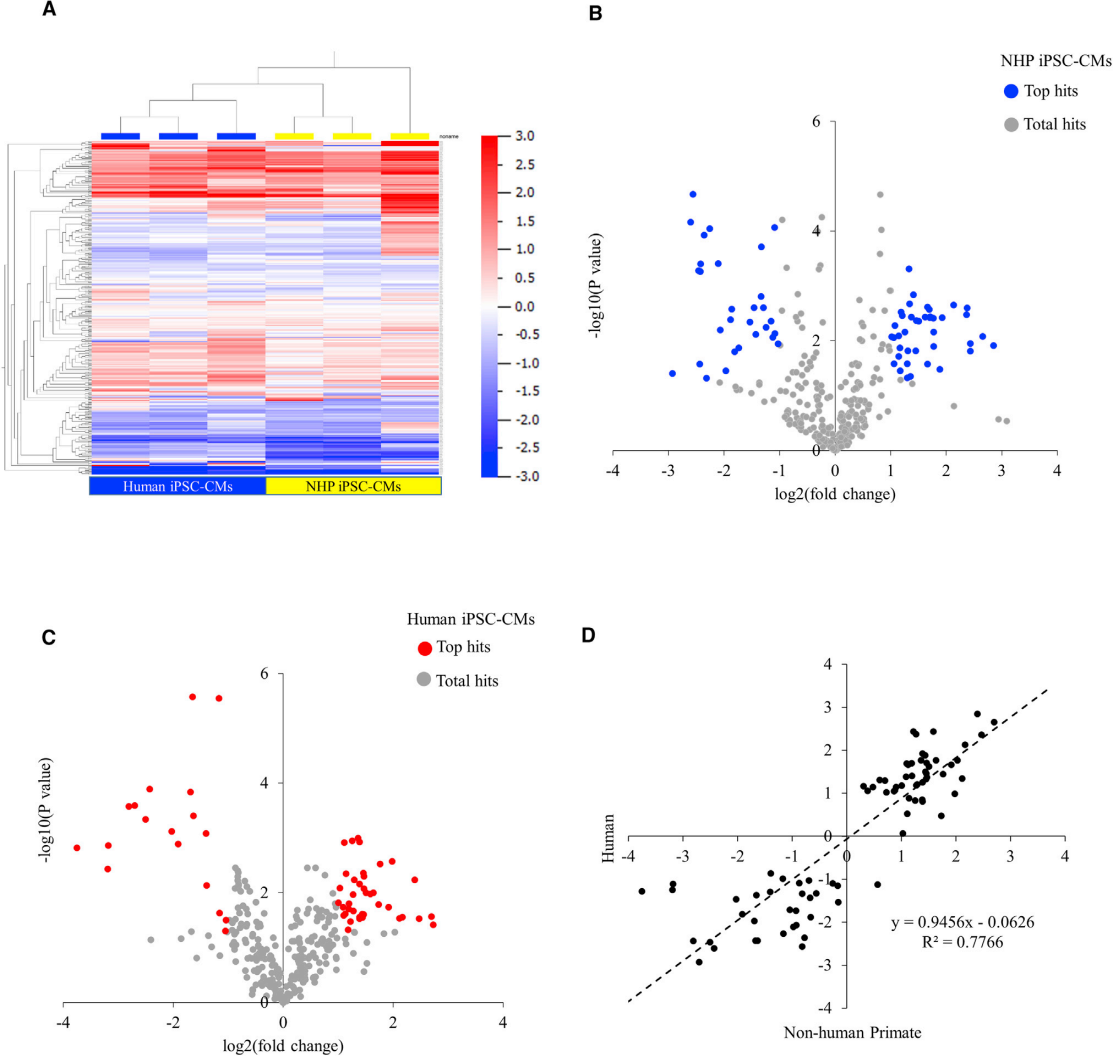
Metabolomic Analysis of the Culture Media in Response to 24-hr Hypoxia (A) Heatmap generated from metabolomic analysis. Relative expression values (log ratios versus normoxic condition) were used. (B) Volcano plot of the total and top regulated (p < 0.05) metabolites seen in NHP iPSC-CMs. (C) Volcano plot of the total and top regulated (p < 0.05) metabolites seen in human iPSC-CMs. (D) Comparison of the metabolites changed more than 2-fold between the two species. n = 3 independent experiments.

To elucidate how paracrine factors secreted from iPSC-CMs affect the microenvironment through metabolic regulation, we collected culture media from NHP and human iPSC-CMs at 24 hr after oxygen depletion for metabolomics analysis. A heatmap was generated based on metabolomics data that compared the fold change of the number of metabolites detected under the hypoxic condition with those under the normoxic condition (Figure 6A). The pattern in the heatmap shows that the signatures of both species are largely similar except for some local differences. A total of 318 variables/metabolites were retained in the volcano plot, among which 70 variables in NHP iPSC-CMs (Figure 6B) and 58 in human iPSC-CMs (Figure 6C) reached statistical difference (p < 0.05). When metabolites that were changed over 2-fold from normoxic condition between the two groups were plotted, the slope of the regression line was 0.95, with a coefficient of determination R^2^ of 0.78, suggesting the majority of the changes in metabolites were proportional between NHP and human iPSC-CMs (Figure 6D). Both groups were found to be involved in regulating ketogenesis, ketolysis, and methylglyoxal-related metabolism (Figure S4A). However, metabolites from NHP iPSC-CMs were more involved in glycogenolysis, whereas the metabolites from human iPSC-CMs were related to lactose degradation and citric acid cycle (Figures S4B and S4C).

## Discussion

Our study compared the similarities and differences between NHP iPSC-CMs and human iPSC-CMs for treatment of subacute MI in a rodent model. For clinical relevance, rather than using permanent coronary artery ligation as used by previous studies (Citro et al., 2014, Lepperhof et al., 2014, Ong et al., 2015), we utilized the ischemia-reperfusion model to simulate MI patients with re-established blood supply after intervention. In addition, instead of acute treatment within 24 hr after MI (Carpenter et al., 2012, Lepperhof et al., 2014, Ong et al., 2015), we injected cells at day 4 post infarction, which is the subacute stage of MI, to simulate the delay for iPSC-CM match and preparation for cell transplantation. After cell injection, both NHP and human iPSC-CM groups showed similar improvement in LV contractility at 2 weeks post transplantation, and this improvement was preserved at week 4. Furthermore, both echocardiography and hemodynamic analysis demonstrate the improvement in both systolic and diastolic function as shown by LVEF and tau. To prove that such an effect is specific to iPSC-CMs, we analyzed a separate negative cohort with functionally unrelated NHP or human fibroblasts. Similar to previous studies (Kolossov et al., 2006, Yeghiazarians et al., 2012), during the 4-week period after cell delivery, fibroblast-treated groups did not alter cardiac function, and severe LV chamber dilatation was observed at the end of the 4-week period. It is worth noting that while the end-diastolic volume continuously increased after cell transplantation in the control groups, the end-systolic volume was markedly reduced in both iPSC-CM-treated groups. This continual dilatation and remodeling in end-diastolic volume may represent a compensatory mechanism in response to increased wall stress (Grossman, 1980). In previous studies using iPSC-CMs from different species, the changes in LV function varied from study to study (Citro et al., 2014, Wu et al., 2016). In our current study, both NHP and human iPSC-CMs showed similar potency in improving LV function and attenuation in chamber dilatation.

This similarity in functional improvement was accompanied by similar changes at the histological levels in both NHP and human iPSC-CMs. We noted that within the ischemic zone, the total viable tissues (composed of grafts and host myocardium) were present to a similar extent between the two iPSC-CM-treated groups. In addition to the increased viable tissue and reduced scar size, we noted a smaller cardiomyocyte size and less interstitial fibrotic tissue in both iPSC-CM-treated groups at both BZ and RZ. This indicates a similar attenuation in remodeling with less hypertrophy and less reactive fibrosis. Moreover, consistent with previous studies showing angiogenesis in the iPSC-CM-treated MI hearts (Lepperhof et al., 2014, Ye et al., 2014), we also found that the capillary density was similarly increased in both iPSC-CM-treated groups at the BZ and RZ. Although the total amount of grafts was difficult to quantify accurately at 4 weeks after cell transplantation, we observed that a small amount of grafts exist at different sections of the LV wall in both iPSC-CM-treated groups. They presented as either free cells or clusters at the ischemic zone and BZ, but there was no sign of remuscularization or contact between engraftment and host myocardium as reported in other studies (Chong et al., 2014, Shiba et al., 2016). Therefore, the improved function was not directly due to the engraftment in the ischemic zone. Comparing the size of the grafts with the whole LV wall, it is reasonable to infer that paracrine effects of iPSC-CMs play a pivotal role in maintaining cardiac function and attenuating myocardial remodeling. Indeed, numerous studies have shown that adult stem cells and their derived cells affect the microenvironment via paracrine actions (Mirotsou et al., 2007, Ong et al., 2015, Ye et al., 2015).

To further understand post-transplantation response of the graft to the host ischemic myocardium, we simulated the oxygen-depletion condition the grafts encountered *in vivo*, and compared the transcriptomic and metabolomic profiles of both iPSC-CMs *in vitro*. Given the species difference, it was not surprising to see certain differences in the iPSC-CMs at the molecular level. Among all genes that were significantly up- or downregulated, RNA-seq revealed only a small portion of shared genes between NHP and human iPSC-CMs. Despite few overlapping genes, we noted that many biological functions were regulated similarly toward the same direction. This finding suggests that in addition to using some of the shared genes, NHP and human iPSC-CMs utilized different sets of genes to achieve the same *in vivo* outcomes. For instance, along with a similar increase in capillary density *in vivo*, the RNA-seq showed a comparable upregulation in VEGF and angiogenin. However, IGF receptor (Bid et al., 2012) was significantly upregulated only in NHP iPSC-CMs, whereas platelet-derived growth factor (Moriya et al., 2014) was upregulated more in human iPSC-CMs. These phenomena also applied to the genes promoting cell survival and inhibiting fibrosis and hypertrophy (Figures S2 and S3), as well as transcription factors that are involved in cardioprotection (Table S5). Although *in vivo* analysis showed that the protection against MI was similar between the two groups, RNA-seq and pathway analysis revealed that fewer genes were involved in these related pathways in human iPSC-CMs compared with NHP iPSC-CMs. Transcription factors that control these genes were changed more dramatically in NHP iPSC-CMs (Table S5).

We also found that some pathways were differentially activated between NHP and human iPSC-CMs, indicating a species-specific response to hypoxia. Interestingly, VEGF was found to be upregulated in both NHP and human iPSC-CMs. VEGF is implicated in all four cardioprotective biological functions, promoting cell survival and angiogenesis as well as inhibiting hypertrophy and fibrosis. Based on IPA, the VEGF gene encodes a protein that will be eventually secreted to extracellular space. Indeed, proteomic analysis of the culture media after oxygen depletion also showed an increased VEGF level. VEGF has been demonstrated by several studies to confer benefits to the ischemic heart (Byrne et al., 2005, Hoeben et al., 2004, Park et al., 2009, Xu et al., 2011). Although gene therapy for MI with VEGF failed in clinical trials (Kastrup et al., 2005, Stewart et al., 2009), its contribution to cardioprotection cannot be excluded.

In addition to VEGF, we also found other hypoxia-induced differentially expressed genes shared by both NHP and human iPSC-CMs. Jumonji domain-containing protein 6 (JMJD6) is an arginine demethylase known to demethylate histone H3 at arginine 2 and histone H4 at arginine 3 (Chang et al., 2007) and found to be upregulated in hypoxia-treated cardiomyocytes in our RNA-seq data. Previous reports have shown that JMJD6 regulates RNA splicing through modification of splicing factor U2 small nuclear ribonucleoprotein auxiliary factor 65-kDa subunit (Webby et al., 2009) and is required in endothelial cells to regulate the splicing of VEGF receptor as well as angiogenic sprouting (Boeckel et al., 2011). Despite its expression in cardiomyocytes as shown by immunostaining (Human Protein Atlas, www.proteinatlas.org), the specific role of JMJD6 in cardiomyocytes under hypoxia condition is not known and may require additional investigation. Moreover, we found some genes to be downregulated by hypoxia in both NHP and human iPSC-CMs, including MYB Proto-Oncogene Like 2 (MYBL2), also known as B-MYB. MYBL2 is a cyclin-dependent kinase and has been shown to activate genes during the S phase of the cell cycle (Joaquin and Watson, 2003). It is unclear how the downregulation of MYBL2 facilitates the ischemic response of cardiomyocytes, but downregulation of the cell cycle has been shown to protect cells from DNA damage or ischemia-induced cell death (Nowsheen and Yang, 2012). Taken together, our RNA-seq data suggest common pathways for both NHP and humna iPSC-CMs in response to ischemia.

The metabolomic profile of the two types of iPSC-CMs in response to oxygen depletion was another parameter we examined in comparing the two species. Unlike changes in the transcriptomic profile in which a small number of common genes were shared between the two species, the changes in secreted metabolites were much more alike. This similarity existed not only in the direction of the changes but also in the extent of the changes of individual metabolites (Figure 6D). In examining the top changed metabolic pathways, we noted, along with certain similarities between the two species, that there were more regulated pathways unique to one species (Figures S4B and S4C).

One potential limitation of our study is the absence of a large animal model due to prohibitive costs (e.g., transplantation of NHP or human iPSC-CMs into NHP). Allogeneic transplantation with rodent iPSC-CM is also an option. However, current protocols do not allow for the generation of rat or mouse iPSC-CMs at a sufficient amount and purity level (Liao et al., 2009, Liu et al., 2013, Merkl et al., 2013, Takenaka-Ninagawa et al., 2014, Yamaguchi et al., 2014). Nevertheless, our observation of comparable improvement with either NHP or human iPSC-CMs in a xenogeneic rodent model allows an objective comparison between these two species. Using the current model we could normalize confounding factors such as rejection and arrhythmia, thus creating no bias against either NHP or human iPSC-CM transplantation. Finally, our experiments provide additional information regarding functional, transcriptomic, and metabolomic changes between NHP and human iPSC-CMs that may lead to better preclinical study designs in the future.

Our study provides the cross-species comparison of iPSC-CMs for the treatment of MI in a xenogeneic rodent model. Given the phylogenetic distance between human and rhesus (23.3 million years) (Kumar and Hedges, 1998), it is not surprising to observe differences and similarities when comparing NHP with human iPSC-CMs. Although the therapeutic efficacy *in vivo* and common pathways *in vitro* between the two types of iPSC-CMs were very similar, there were nevertheless differences at the transcriptomic and metabolomic levels. Future plans to study individual genes or metabolic pathways in NHP iPSC-CMs for predicting outcome in human iPSC-CMs should proceed with caution due to these differences, the exact significance of which awaits further investigation.

## Experimental Procedures

For an extended description of methods, please refer to Supplemental Experimental Procedures.

### Culture and Maintenance of NHP and Human iPSCs

Fibroblasts from a rhesus macaque monkey and healthy male human donor were reprogrammed into iPSCs via Sendai virus vectors carrying the Yamanaka reprogramming factors (Oct4, Sox2, Nanog, and cMyc). Both NHP and human iPSCs were grown to 90% confluence (Lan et al., 2013, Sun et al., 2009) on Matrigel-coated plates (ES Qualified, BD Biosciences, San Diego, CA) using a chemically defined E8 medium as previously described (Chen et al., 2011). The medium was changed daily, and cells were passaged every 3–4 days with EDTA (Thermo Fisher Scientific, CA).

### Cardiac Differentiation

Both NHP and human iPSCs were grown to 90% confluence and subsequently differentiated into beating cardiomyocytes as previously described (Ebert et al., 2014, Liang et al., 2016). In brief, on day 0 cells were supplemented with a basal medium (RPMI 1640 [Thermo Fisher Scientific] and 2% B27 supplement minus insulin [Thermo Fisher Scientific]), with the addition at a concentration of 2–8 μM of CHIR-99021 [Selleck Chemicals], a selective inhibitor of glycogen synthase kinase 3β, which activates the canonical Wnt signaling pathway. On day 2, the medium was replaced with basal medium without CHIR99021 supplementation. On day 3, 5 μM of IWR-1 [Selleck Chemicals], a Wnt antagonist, was added to basal medium for 2 days. On day 5 and every subsequent other day until harvest, the medium was replaced with fresh basal medium.

### Cell Transplantation

Four days after reperfusion, animals were randomly grouped into five groups: (1) 1 × 10^7^ NHP iPSC-CMs (n = 14), (2) 1 × 10^7^ human iPSC-CMs (n = 14), (3) 1× PBS (n = 14), (4) 1 × 10^7^ human fibroblast (n = 9), and (5) 1 × 10^7^ NHP fibroblast (n = 9). The chest was reopened and cells or PBS were injected intramyocardially at 2–3 sites along the edge of the infarct zone with a total volume of 70 μL with a 28-gauge insulin syringe. Study protocols were approved by the Stanford Animal Research Committee. Animal care was provided in accordance with the Stanford University School of Medicine guidelines and policies for the use of laboratory animals.

### Echocardiography

At 1 day before injection, and 2 and 4 weeks after injection, cardiac function was assessed using transthoracic echocardiography (Vevo 2100 Imaging System, VisualSonics). Animals were anesthetized with 2% inhaled isoflurane, and LV internal dimensions were measured in systole and diastole using leading-edge methods and guidelines of the American Society of Echocardiography (Sahn et al., 1978). LV systolic function was estimated using the Vevo Lab software for calculation.

### *In Vitro* Oxygen Depletion Treatment

At 30 days after differentiation, 3 × 10^6^ NHP iPSC-CMs and 3 × 10^6^ human iPSC-CMs were collected and replated onto Matrigel-coated 6-well plates. Culture media were changed every 2 days for 5 days. On day 5, half of the plates were placed in an anaerobic pouch (GasPak EZ Anaerobe Pouch System, Fisher Scientific, MA) without changing culture media and the other half were left untouched. The pouch was sealed with an anaerobic indicator. The color of indicator changed to blue for the anaerobic conditions. Cells were incubated for another 24 hr before media from both hypoxic and normoxic iPSC-CMs were collected for proteomic VEGF analysis. Cells were collected for transcriptomic profile analysis.

### RNA Sequencing

Kits used for RNA-seq were purchased from Thermo Fisher. Total RNA was mixed with ERCC RNA Spike-In Mix 1 and polyadenylated mRNA was isolated using the Dynabeads mRNA DIRECT Micro Purification Kit. A cDNA library was prepared using the Ion Total RNA-seq Kit v2 and sequenced by the Ion Proton using Ion PI Hi-Q Sequencing kit. Unaligned BAM files were generated by the Ion Torrent Suite 5.0.4 and converted into FASTQ files. The reads for three replicates for human or NHP iPSC-CMs, with or without oxygen depletion, were mapped to reference genome hg19 (human) or rheMac8 (NHP) using TopHat (v2.0.13), assembled with Cufflinks (v2.2.1), and differentially expressed genes induced by oxygen depletion treatment were identified by Cuffdiff (v2.2.1). Because the rhesus macaque genome is not well annotated, human genome (RefSeq Genes) and the rhesus macaque genome were aligned for annotation, resulting in the same gene IDs for both human and rhesus macaque. Molecular pathways and disease or toxicological functions of differentially expressed genes (false discovery rate <0.05, log ratio >1.5) were analyzed by IPA (Qiagen).

### Statistical Analysis

Data are expressed as mean ± SEM. Statistical significance was determined using Student's t test or ANOVA test evaluations. p < 0.05 was taken as a minimal level of significance.

## Author Contributions

X.Z. conceived the study, designed the experiments, performed *in vivo* cardiac function assessments and *in vitro* hypoxic assay, and wrote the manuscript. H.C. examined iPSC-CM purity, performed RNA-seq, analyzed metabolomic data, and wrote the manuscript. D.X. differentiated the cells, characterized the cell lines, performed and analyzed echocardiography, and wrote the manuscript. I.I. and P.S. performed electrophysiology. H.Y. analyzed and characterized the histological findings. X.Q. performed echocardiography. T.C., A.H., Y.Z., B.C.N., J.Z.Z., Y.K., and M.Z. differentiated and characterized the cell lines. A.A., K.L., and M.J. performed metabolomic analysis. E.N. supervised animal experiments, including surgeries and histology. W-H.Z. contributed to the experimental design. J.C.W. conceived the idea and provided experimental advice, manuscript writing, and funding support. All authors reviewed the manuscript.

## Accession Numbers

The accession number for the raw and processed data from the RNA-seq experiments reported in this paper is GEO: GSE108676.

## Supplemental Information

Document S1. Supplemental Experimental Procedures, Figures S1–S4, and Tables S1–S3 and S5

Table S4. Shared Genes between NHP iPSC-CMs and Human iPSC-CMs after 24-hour Hypoxia

Document S2. Article plus Supplemental Information
