# Supplementary material for: Comparison of Non-human Primate versus Human Induced Pluripotent Stem Cell-Derived Cardiomyocytes for Treatment of Myocardial Infarction
Source: Stem Cell Reports. 2018 Feb 1;10(2):422–35. doi: 10.1016/j.stemcr.2018.01.002 (PMC5830958; doi:10.1016/j.stemcr.2018.01.002)
Supplement: Document S1. Supplemental Experimental Procedures, Figures S1–S4, and Tables S1–S3 and S5 [file mmc1.pdf]

Stem Cell Reports, Volume *10*

Supplemental Information

Comparison of Non-human Primate versus Human Induced Pluripotent Stem Cell-Derived Cardiomyocytes for Treatment of Myocardial Infarction

Xin Zhao, Haodong Chen, Dan Xiao, Huaxiao Yang, Ilanit Itzhaki, Xulei Qin, Tony Chour, Aitor Aguirre, Kim Lehmann, Youngkyun Kim, Praveen Shukla, Alexandra Holmström, Joe Z. Zhang, Yan Zhuge, Babacar C. Ndoye, Mingtao Zhao, Evgenios Neofytou, Wolfram-Hubertus Zimmermann, Mohit Jain, and Joseph C. Wu

STEM-CELL-REPORTS-D-17-00202

Supplemental Materials

Comparison of Non-Human Primate vs. Human Induced Pluripotent Stem Cell-Derived Cardiomyocytes for Treatment of Myocardial Infarction

Xin Zhao[1, 2, 7], Haodong Chen[1, 2, 7], Dan Xiao[1, 2, 7], Huaxiao Yang[1, 2], Ilanit Itzhaki[1, 2], Xulei Qin[1, 2], Tony Chour[1, 2], Aitor Aguirre[3], Kim Lehmann[3], Youngkyun Kim[1, 2], Praveen Shukla[1, 2], Alexandra Holmström[1, 2], Joe Z. Zhang[1, 2], Yan Zhuge[1, 2], Babacar C. Ndoye[1, 2], Mingtao Zhao[1, 2], Evgenios Neofytou[1, 2], Wolfram-Hubertus Zimmermann[4, 5], Mohit Jain[3], Joseph C. Wu[1, 2, 6*]

[1]Stanford Cardiovascular Institute, Stanford, California

[2]Institute for Stem Cell Biology and Regenerative Medicine, Stanford, California

[3]Departments of Medicine and Pharmacology, University of California, San Diego, California

[4]Institute of Pharmacology and Toxicology, University Medical Center, Goettingen, Goettingen, Germany

[5]DZHK (German Center for Cardiovascular Research, partner site Goettingen, Germany

[6]Department of Medicine, Division of Cardiology, Stanford University School of Medicine, Stanford, California

[7]Authors (X.Z., H.C. and D.X.) contributed equally to this study

STEM-CELL-REPORTS-D-17-00202

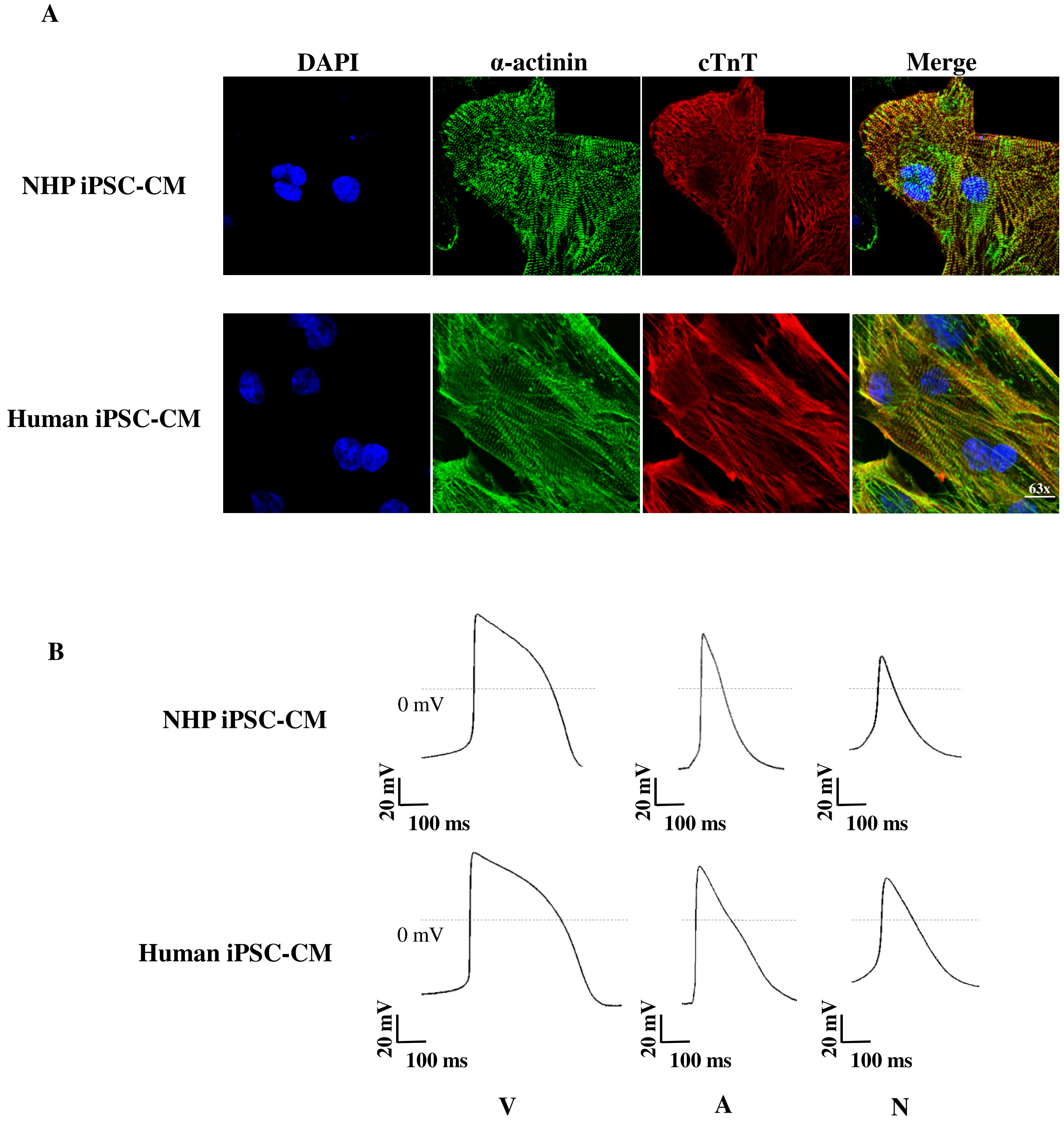

Figure S1. Histological and electrophysiological characterization of NHP iPSC-CMs and human iPSC-CMs. **(A)** Representative iPSC-CM structures stained with α-actinin (green), cardiac troponin T (red), and DAPI (blue). Thirty-day differentiated iPSC-CMs showed well-aligned sarcomere structure. **(B)** Representative recordings of the three major CM action potential (AP) subtypes using whole cell patch clamp. Cells exhibit ventricular-like (V), atrial-like (A), or nodal-like (N) AP morphology.

A Cell Survival

NHP iPSC-CMs

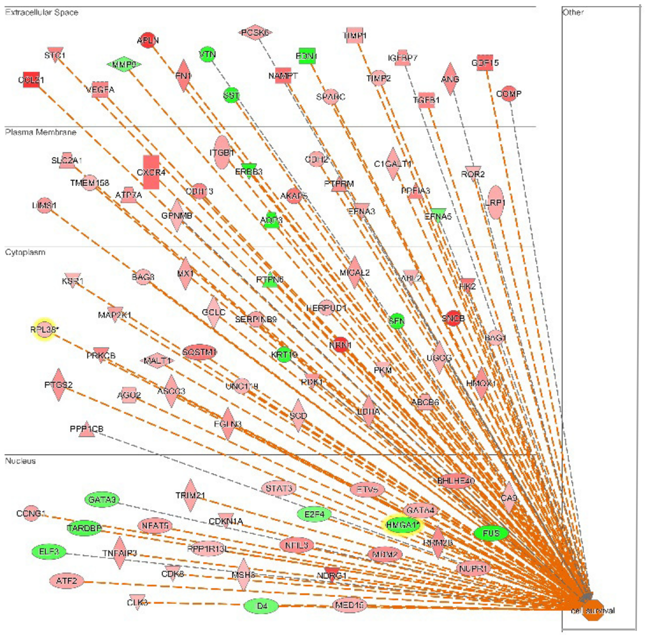

Human iPSC-CMs

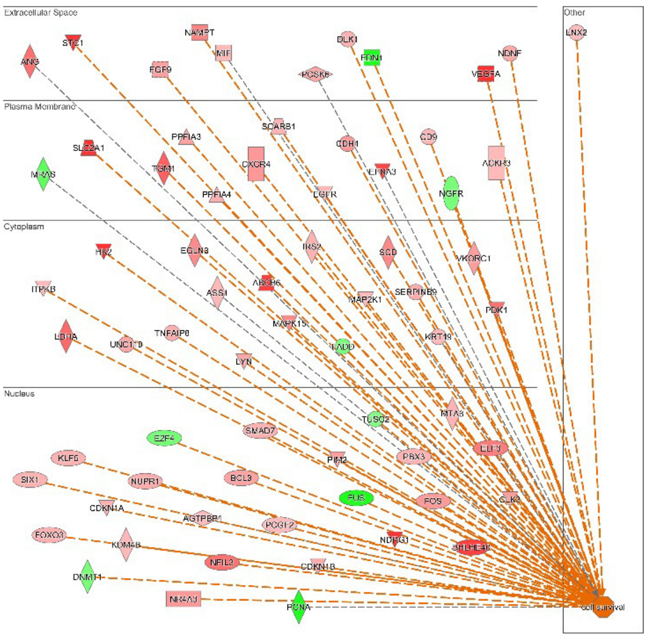

B Angiogenesis

NHP iPSC-CMs

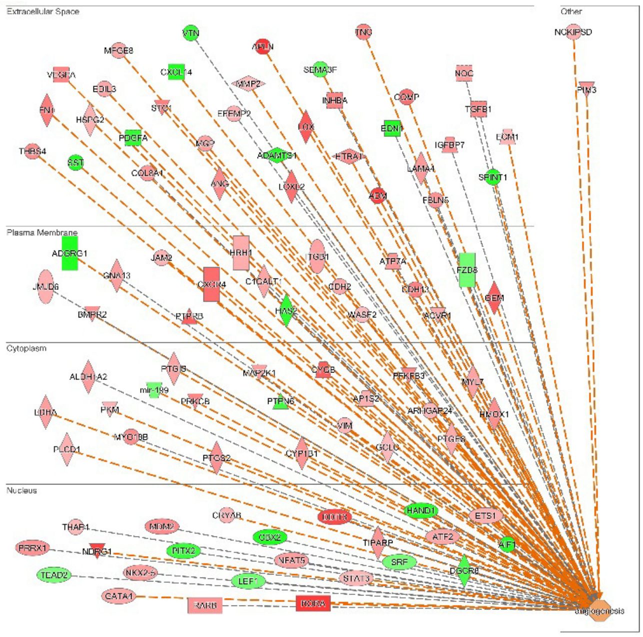

Human iPSC-CMs

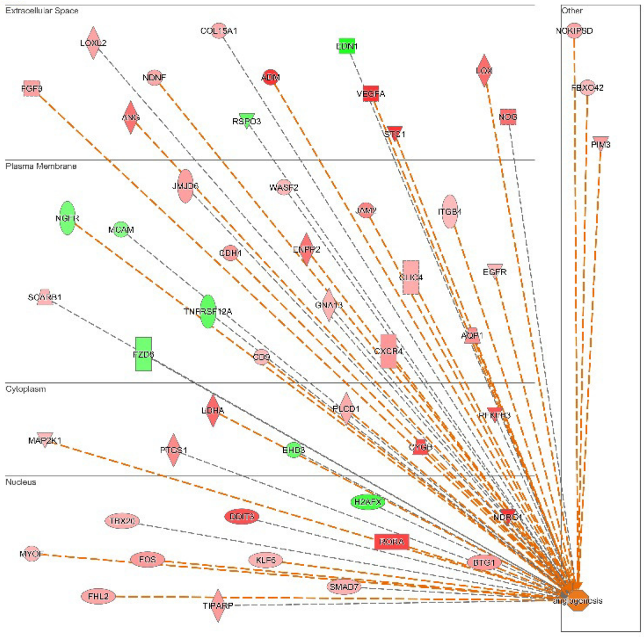

Figure S2. IPA analysis from RNA-seq categorized the significantly regulated genes according to extracellular matrix, membrane, cytosol, and nucleus. Genes in red means upregulation, genes in green means downregulation, and the dash lines in red suggest the gene promotes the pathway. **(A)** Genes responsible for cell survival. **(B)** Genes responsible for angiogenesis.

STEM-CELL-REPORTS-D-17-00202

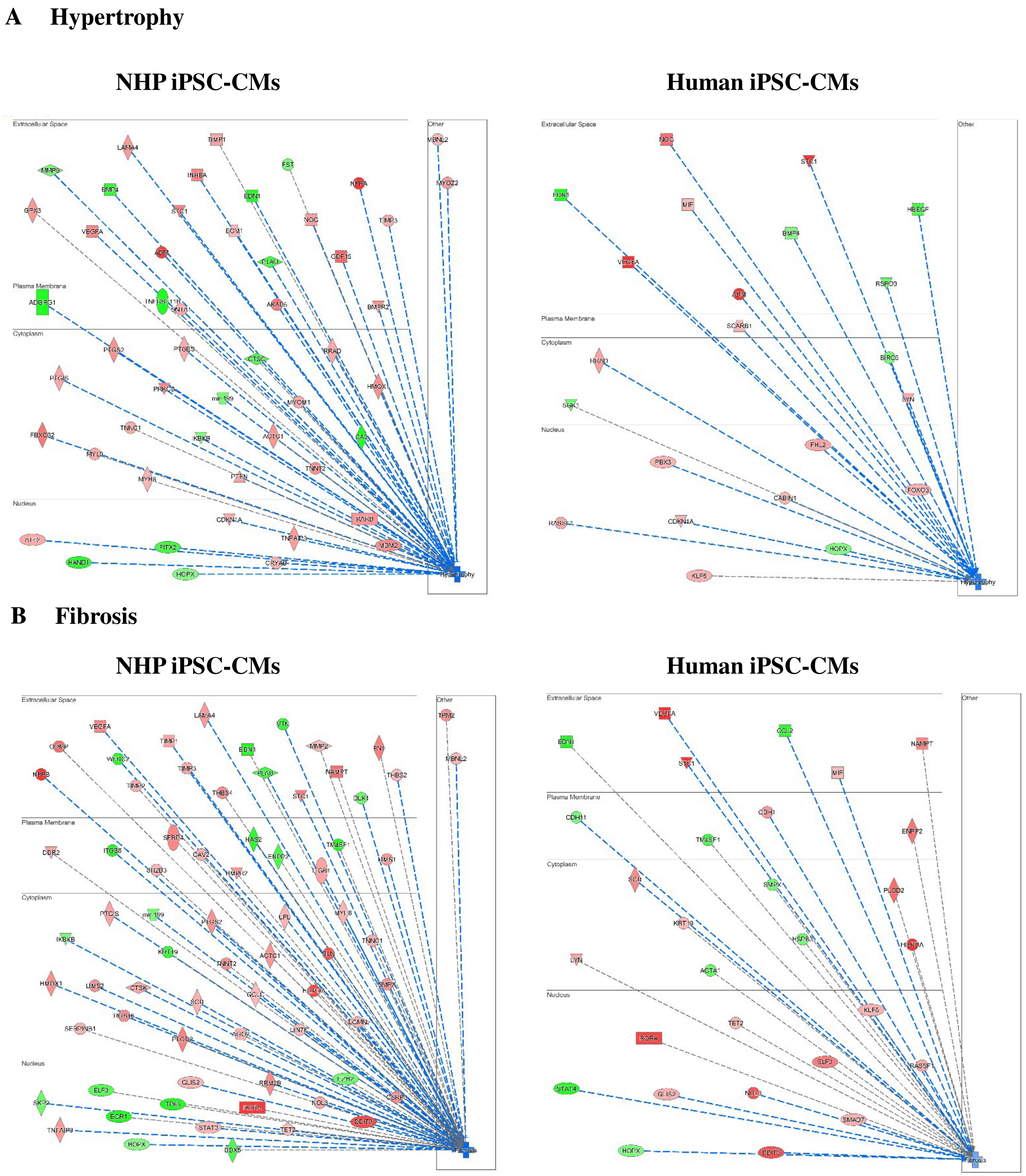

Figure S3. IPA analysis from RNA-seq categorized the significantly regulated genes according to extracellular matrix, membrane, cytosol, and nucleus. Genes in red means upregulation, genes in green means downregulation, and the dash lines in blue suggest the gene inhibits the pathway. **(A)** Genes responsible for hypertrophy. **(B)** Genes responsible for fibrosis.

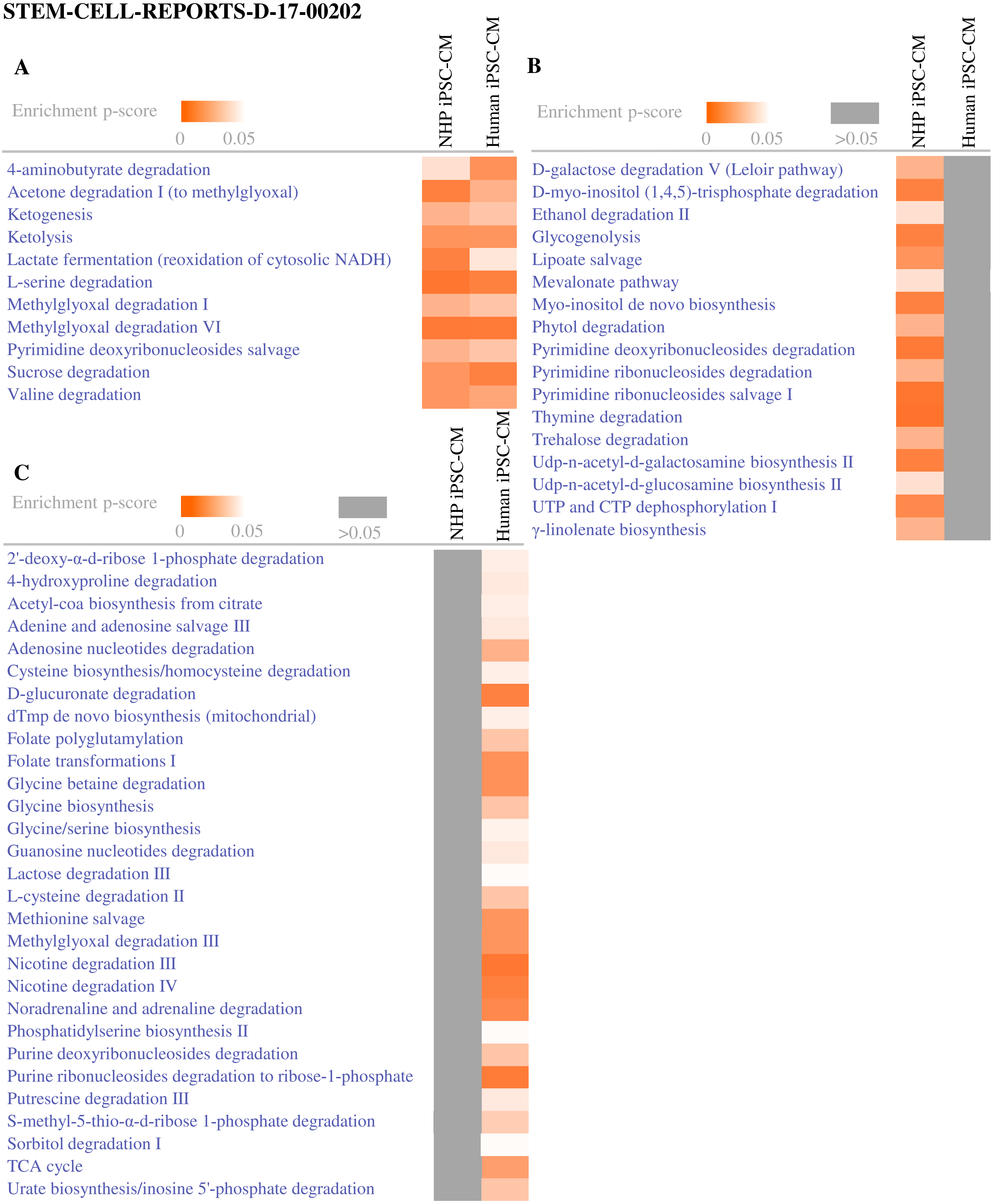

Figure S4. Metabolomics analysis of metabolism related pathways in response to oxygen depletion. **(A)** Common pathways shared between the two species. **(B)** Pathways that are significantly regulated in NHP iPSC-CMs, but not in human iPSC-CMs (grey color). **(C)** Pathways that are significantly regulated in human iPSC-CMs, but not in NHP iPSC-CMs (grey color). N=3.

Table S1: Summary of action potential parameters of NHP and human iPSC-CMs

NHP iPSC-CMs	MDP (mv)	APA (mV)	Overshoot (mV)	Upstroke Velocity (V/sec)	APD50 (msec)	APD70 (msec)	APD90 (msec)	Beating rate (bpm)
**Ventricular-like**	-65 ± 9.2	107 ± 6.0	43 ± 8.6	19 ± 1.3	150 ± 56	174 ± 65	193 ± 71	81 ± 12
**Atrial-like**	-62 ± 4.2	102 ± 7.3	40 ± 7.8	25 ± 15	105 ± 53	130 ± 56	159 ± 58	89 ± 35
**Nodal-like**	-47 ± 3.7	72 ± 2.7	25 ± 6.4	3.4 ± 0.2	71 ± 11	101 ± 25	146 ± 50	134 ± 72
**Human iPSC-CMs**								
**Ventricular-like**	-64 ± 3.9	113 ± 9.1	49 ± 5.8	21 ± 7.7	230 ± 67	264 ± 73	290 ± 78	60 ± 18
**Atrial-like**	-64 ± 4.5	105 ± 7.4	40 ± 9.2	31 ± 20	133 ± 49	169 ± 61	208 ± 72	66 ± 30
**Nodal-like**	-51 ± 2.9	82 ± 10	32 ± 6.9	3.1 ± 1.7	79 ± 26	102 ± 34	130 ± 41	133 ± 28

Results are provided as mean ± SEM. MDP: maximal diastolic potential. APA: action potential amplitude. APD50: action potential duration at 50% repolarization. APD70: action potential duration at 70% repolarization. APD90: action potential duration at 90% repolarization. bpm: beats per minute.

Table S2: Echocardiography assessment before cell injection

	PBS (n=14)	NHP Fibroblast (n=9)	NHP iPSC-CM (n=14)	Human fibroblast (n=9)	Human iPSC-CM (n=14)
LV Ejection Fraction (%)	48.4±1.7	36.0±1.3	48.2±2.9	34.1±3.4	47.9±2.0
LV Fractional Shortening (%)	25.1±1.1	17.9±0.7	25.1±1.8	17.0±1.9	24.9±1.2
LV End-diastolic Volume (µl)	216±13	295±19	193±10	285±17	210±8.2
LV End-systolic Volume (µl)	113±9	190±14	101±9	191±19	110±6.8

Data are presented as Mean ± SE

STEM-CELL-REPORTS-D-17-00202

Table S3: Cardiac function at 4 weeks after cell injection

	PBS (n=7)	NHP Fibroblast (n=9)	NHP iPSC-CM (n=11)	Human fibroblast (n=9)	Human iPSC-CM (n=12)
Heart Rate (bpm)	356±13	372±14	353±7.9	357±9	345±9.9
LV Systolic Pressure (mmHg)	113±4.3	99±2.0	111±2.4	101±2.4	112±2.6†
Mean Arterial Pressure (mmHg)	105±5.5	91±2.9	98±3.0	90±2.3	100±3.0
LV Maximum dP/dt (mmHg/s)	6628±103	5835±242	7497±316$^{*\#}$	6385±259	7991±434*†
LV Minimum dP/dt (mmHg/s)	-5327±101	-5357±333	-6393±385$^{*\#}$	-5633±196	-6740±499‡
End-diastolic Pressure (mmHg)	8.2±0.9	12±0.8	5.4±0.6$^{**\#}$	8.6±0.7	5.2±0.7†‡
Tau (ms)	11±0.4	12±0.4	9.8±0.4**	12±0.3	9.7±0.3††‡

Data are presented as mean ± SEM; *p<0.05, **p<0.01 NHP iPSC-CM vs. NHP fibroblast; $^{\#}$p<0.05, $^{\#\#}$p<0.01 NHP iPSC-CM vs. PBS; †p<0.05, ††p<0.01 human iPSC-CM vs. human fibroblast; and ‡p<0.05, ‡‡p<0.01 human iPSC-CM vs. PBS.

STEM-CELL-REPORTS-D-17-00202

Table S5: Comparison of transcriptional factors regulated after hypoxia in NHP iPSC-CMs and human iPSC-CMs

NHP iPSC-CM					Human iPSC-CM			
Term	Count	%	Benjamini		Term	Count	%	Benjamini
SRF	319	76.7	2.50E-10		SRF	193	72.6	2.90E-04
MEF2	330	79.3	4.80E-10		MEF2	204	76.7	6.40E-04
SOX5	234	56.2	1.60E-09		SOX5	133	50.0	3.10E-03
RSRFC4	228	54.8	4.90E-09		RSRFC4	134	50.4	1.20E-03
USF	266	63.9	1.30E-07		USF	167	62.8	2.20E-04
TATA	247	59.4	2.10E-07		TATA	147	55.3	3.10E-03
HNF1	252	60.6	2.20E-07		HNF1	160	60.2	2.90E-04
AP1	269	64.7	2.20E-07		AP1	170	63.9	2.40E-04
GATA1	342	82.2	2.20E-07		GATA1	218	82.0	4.30E-04
FOXO4	247	59.4	2.40E-07		FOXO4	150	56.4	1.50E-03
FOXJ2	289	69.5	2.50E-07		FOXJ2	181	68.0	4.30E-04
CDC5	211	50.7	2.70E-07		CDC5	128	48.1	1.30E-03
LMO2COM	262	63.0	4.60E-07		LMO2COM	165	62.0	4.70E-04
CDP	266	63.9	5.10E-07		CDP	166	62.4	8.40E-04
FREAC7	223	53.6	5.10E-07		FREAC7	134	50.4	3.00E-03
POU3F2	262	63.0	5.30E-07		POU3F2	165	62.0	5.00E-04
ARNT	219	52.6	5.50E-07		ARNT	141	53.0	3.20E-04
NKX61	202	48.6	1.20E-06		NKX61	123	46.2	2.40E-03
CEBPB	245	58.9	2.40E-06		CEBPB	156	58.6	5.10E-04
STAT5A	253	60.8	2.60E-06		STAT5A	162	60.9	4.60E-04
FREAC4	209	50.2	4.10E-06		FREAC4	137	51.5	3.00E-04
E2F	248	59.6	8.80E-06		E2F	154	57.9	3.00E-03
CEBP	319	76.7	1.00E-05		CEBP	200	75.2	2.90E-03
HNF3B	187	45.0	1.30E-05		HNF3B	122	45.9	5.20E-04
NMYC	150	36.1	1.60E-05		NMYC	93	35.0	3.10E-03
NFY	214	51.4	2.60E-05		NFY	135	50.8	2.70E-03
HLF	183	44.0	2.60E-05		HLF	123	46.2	2.20E-04
FOXO1	198	47.6	2.80E-07		CREB	129	48.5	2.60E-04
HFH3	195	46.9	3.40E-07		NKX25	179	67.3	3.70E-04
TBP	174	41.8	3.90E-07		BRACH	155	58.3	7.30E-04
SRY	199	47.8	4.30E-07		COUP	122	45.9	9.20E-04
MYCMAX	276	66.3	4.30E-07		ATF6	132	49.6	9.40E-04
AHRARNT	238	57.2	5.70E-07		HTF	150	56.4	1.00E-03
IRF7	207	49.8	2.60E-06		FOXO3	87	32.7	1.00E-03
HFH1	206	49.5	3.00E-06		SEF1	143	53.8	1.20E-03
GATA	204	49.0	4.00E-06		SP1	79	29.7	1.30E-03
GCNF	251	60.3	4.00E-06		FAC1	135	50.8	1.40E-03
STAT1	185	44.5	4.20E-06		AML1	204	76.7	1.50E-03
SOX9	202	48.6	4.50E-06		TAXCREB	150	56.4	1.50E-03
SREBP1	261	62.7	7.80E-06		E4BP4	124	46.6	1.60E-03
FREAC3	195	46.9	7.90E-06		MYOD	154	57.9	1.60E-03
1-Oct	360	86.5	1.30E-05		MZF1	136	51.1	1.80E-03
FREAC2	154	37.0	1.30E-05		P53	176	66.2	2.30E-03
PAX4	322	77.4	1.50E-05		BRN2	139	52.3	2.40E-03
MEIS1AHOXA9	163	39.2	1.70E-05		IK3	125	47.0	2.80E-03
EVI1	335	80.5	1.80E-05		ATF	94	35.3	2.80E-03
BACH2	194	46.6	2.40E-05		PAX6	148	55.6	2.80E-03
MSX1	188	45.2	2.40E-05		NKX3A	129	48.5	2.90E-03
FOXD3	159	38.2	2.60E-05		GATA3	73	27.4	3.00E-03
GFI1	198	47.6	2.80E-05		NFE2	102	38.3	3.90E-03

STEM-CELL-REPORTS-D-17-00202

SUPPLEMENTAL METHODS

Whole-cell patch-clamp recordings. NHP and human iPSC-CM monolayers were enzymatically dispersed (Accutase, Sigma) and attached to Matrigel-coated glass coverslips (Warner, USA). Whole-cell patch clamp recordings were conducted using an EPC-10 patch clamp amplifier (HEKA, Germany). 3-4 MΩ glass pipettes were prepared with a micropipette puller (Sutter Instrument, P-97, USA) using thin-wall borosilicate glass (A-M System, USA). Action potentials (APs) were recorded from iPSC-CMs superfused with Tyrode solution at 37°C (TC-324B heating system, Warner, USA). The Tyrode solution consisted of NaCl (140 mM), KCl (5.4 mM), $CaCl_2$ (1.8 mM), $MgCl_2$ (1 mM), HEPES (10 mM), and glucose (10 mM); pH was adjusted to 7.4 with NaOH. The pipette solution consisted of KCl (120 mM), $MgCl_2$ (1 mM), Mg-ATP (3 mM), HEPES (10 mM), and EGTA (10 mM), pH was adjusted to 7.2 with KOH. Data were acquired using PatchMaster software (HEKA, Germany) and digitized at 1.0 kHz. Data were analyzed using a custom-written MATLAB program.

Culture of NHP and human fibroblasts. NHP and human skin fibroblasts were cultured using FGM™ Fibroblast Growth Media Kits (Lonza, Switzerland). Cells were passaged every 5 days.

Myocardial infarction. Sixty male RNU rats (Charles River Laboratories, Wilmington, MA) weighing between 250-350g, were treated with buprenorphine (0.05 mg/kg s.c.) and cefazolin (50 mg/kg i.m.), and then anesthetized with 2% inhaled isoflurane. Rectal temperature was monitored and body temperature was maintained at 37°C with a heating plate. After intubation of the trachea, rats were ventilated with a tidal volume of 0.5 ml/kg at a rate of 90 breaths per minute. A left thoracotomy was performed at the fourth intercostal space and myocardial ischemia was induced by occlusion of the left anterior descending (LAD) coronary

artery against a segment of PE-200 tubing. Sixty minutes after the ligation, the ligature was released and the chest was closed. Buprenorphine (0.05 mg/kg s.c.), carprofen (5 mg/kg, s.c.), and cefazolin (50 mg/kg i.m.) were given as post-operative medications. All operations were performed by a blinded microsurgeon. Study protocols were approved by the Stanford Animal Research Committee. Animal care was provided in accordance with the Stanford University School of Medicine guidelines and policies for the use of laboratory animals.

P-V loop. Animals were anesthetized with 2% inhaled isoflurane, and a multi-segment 1.4F Millar PV-loop catheter (SPR-838, Millar Instruments, TX) was inserted via the right carotid artery into the LV (Zhao et al. , 2012). Adequate placement of the catheter was verified by the PV-loop signals. A 5-0 silk suture was used for inferior vena cava occlusion (IVCO), followed by the abdominal incision closure followed. Baseline hemodynamics was recorded, followed by IVCO. The linear end-systolic pressure-volume relationship (ESPVR) was obtained from the series of pressure-volume relationship regression curves at decreased preloads.

Tissue preparation for capillary density. To measure vessel density, heparinized animals were euthanized with saturated KCl after being anesthetized with 4% isoflurane. The ascending aorta was dissected and cannulated for perfusion of a vasodilation solution and a fixation solution (Limbourg et al. , 2009). Briefly, 10 ml of 1 x Dulbecco's Phosphate-Buffered Saline (DPBS) with heparin (100 U/ml), 10 ml of vasodilation solution [1 x DPBS with adenosine (100 mM) and sodium nitroprusside (10 mM)], and 10 ml of fixation solution [4% paraformaldehyde (PFA) solution (Thermo Fisher Scientific, CA] were infused consecutively at a constant pressure of 80 mmHg.

Histology staining. Hearts were collected and perfused with 4% PFA. The right ventricle was trimmed and LV was transversely sliced into 6 rings with 1.5-2 mm in thickness. LV

samples were stored in PFA at 4ºC for 24 hr, and transferred into 30% sucrose (Sigma Aldrich, MO) afterwards for another 24 hr. Then the LV rings were embedded individually in cryomold molds with the OCT compound (Tissue-Tek, Fisher Scientific, NH). LV rings were sectioned at 10 μm thickness and assessed for the following:

A) Scar size: LV sections from each ring were stained with Masson's trichrome. The scar size was quantified as the percentage of the area of the fibrotic tissue to the area of each LV ring, then normalized by the weight of each LV section before fixation.

B) Graft staining: Both implanted NHP iPSC-CMs and human iPSC-CMs were identified with antibody against human mitochondria and cardiac troponin T (TnT).

C) Interstitial fibrosis: Tissue sections were stained with Picro-Sirius Red (PSR) to identify fibrosis deposition at both border zone and remote zone. Using ImagePro-Plus software, the percentage of total interstitial fibrosis was quantified at 20x magnification (Peter et al. , 2007).

D) Cell size: The profile of endogenous rat cardiomyocytes was recognized using rhodamine-conjugated wheat germ agglutinin (WGA). The circumference of each cardiomyocyte was traced at 40x magnification and quantified using ImagePro-Plus software.

E) Capillary density: Using CD144 (Cell Signaling, MA), capillaries were identified as a single endothelial cell layer with a diameter less than 25 μm. Capillary density was quantified at 40x magnification as the absolute number per unit myocardial area.

Proteomic VEGF assay. Media collected from both cells in both normoxic and anaerobic conditions were centrifuged at 500g for 3 min. Using the Human Proteomic Angiogenesis Assay, 1 ml supernatant was transferred into 1.5 ml Eppendorf tubes with 0.5 ml Array Buffer 4. Fifteen microliter of reconstituted Detection Antibody Cocktail was added to each sample and incubated for one hour. The samples were then added to a 4-well multi-dish containing blotting membrane,

and kept at 4˚C overnight. Membranes were washed in 20 ml wash buffer for 10 min x 3 times and rinsed with deionized water in between. Afterwards, 2 ml of diluted Streptavidin-HRP with Array Buffer 5 was added into each well of the 4-well multi-dish and incubated for 30 min at room temperature. The membrane was then developed with the Chemi Reagent Mix followed by imaging with the Bio-Rad Chemidoc Imaging system. Using Image J software, the intensity of positive blot representing VEGF was analyzed. The final results of both NHP iPSC-CMs and human iPSC-CMs were calculated and presented as fold-change relative to normoxic condition.

RNA preparation. RNA was extracted using RNeasy Mini Kit (Qiagen, Germany). Briefly, cultured iPSC-CMs were homogenized in Trizol (1 ml / 5 x 10^6 cells, Roche, Switzerland) and chloroform (0.2 ml) was added. After vortexing and incubating at room temperature for 10 min, samples were centrifuged at 12,000 rpm at 4°C for 20 min. The upper aqueous phase containing RNA was collected with equal volume of 70% ethanol added, and the resulting mixture was loaded into an isolation column provided with the kit. After washing several times with wash buffer, RNA was eluted from the column with nuclease free water. RNA quality was tested using NanoDrop (Thermo Scientific, DE) with the criterion of $1.8 < A_{260/280} < 2.0$.

Liquid chromatography-mass spectrometry (LC-MS). Media samples were briefly vortexed and 50 µL of each were taken to a new microfuge tube. 200 µL of cold methanol with an internal standard (^{13}C glutamine, 50 uM concentration in each sample) was added to each tube. Samples were then vortexed for 30 sec and allowed to sit at -80˚C for 30 min to facilitate protein precipitation. Samples were vortexed again for 30 sec, followed by centrifugation at 14,000 rpm for 10 min at 4˚C, after which 50 µL of supernatant was transferred to glass LC-MS vial. For LC-MS, sample volumes were transferred to glass vials and kept at 4˚C in the autosampler compartment until 1 µl of sample was injected for analysis. Compounds were separated using a

Thermo Vanquish UPLC coupled to a Thermo QExactive Orbitrap mass spectrometer. Separation was performed using a Millipore (Sequant) Zic-pHILIC 2.1 × 150 mm 5 µm column maintained at 25˚C using a flow rate of 0.3 mL/min and a 15 min linear gradient starting from 90:10 acetonitrile: 20 mM ammonium bicarbonate, pH 9.6 to 45:55 acetonitrile: 20 mM ammonium bicarbonate, pH 9.6. Detection was performed in positive and negative ion modes through sequential sample injections using a heated electrospray ionization (HESI) source operated at 2.5 kV (negative mode) and 3.5 kV (positive mode), sheath gas flow of 40, auxiliary gas flow of 20, sweep gas flow of 2, capillary temperature of 275˚C, and auxiliary gas heater temperature of 350˚C. Data were collected using data-dependent tandem MS collection with MS1 parameters of 70,000 mass resolution, 100 ms maximum IT time, 3×10^6 AGC volume, a mass range of 67 to 1000 m/z, MS2 parameters of 17,500 mass resolution, 50 ms maximum IT time, 1×10^5 AGC volume, loop count of 5, isolation window of 0.5 m/z, NCE of 35, and a 10-second dynamic exclusion. When possible, LC-MS peaks were assigned metabolite identity by matching accurate mass and retention time against pure standards. Data extraction and analysis were performed using Mzmine, XCMS, and a combination of in-house developed tools. Metabolite pathway enrichment was obtained from Metlin libraries.

STEM-CELL-REPORTS-D-17-00202

Reference

Limbourg, A., Korff T., Napp L.C., Schaper W., Drexler H., Limbourg F.P. (2009). Evaluation of Postnatal Arteriogenesis and Angiogenesis in a Mouse Model of Hind-Limb Ischemia. Nat Protoc *4*, 1737-1746.

Peter, P.S., Brady J.E., Yan L., Chen W., Engelhardt S., Wang Y., Sadoshima J., Vatner S.F., Vatner D.E. (2007). Inhibition of P38 Alpha Mapk Rescues Cardiomyopathy Induced by Overexpressed Beta 2-Adrenergic Receptor, but Not Beta 1-Adrenergic Receptor. J Clin Invest *117*, 1335-1343.

Zhao, X., Park J., Ho D., Gao S., Yan L., Ge H., Iismaa S., Lin L., Tian B., Vatner D.E., *et al.* (2012). Cardiomyocyte Overexpression of the Alpha1a-Adrenergic Receptor in the Rat Phenocopies Second but Not First Window Preconditioning. Am J Physiol Heart Circ Physiol *302*, H1614-1624.
