# Supplementary material for: Comparison of Non-human Primate versus Human Induced Pluripotent Stem Cell-Derived Cardiomyocytes for Treatment of Myocardial Infarction
Source: Stem Cell Reports. 2018 Feb 1;10(2):422–35. doi: 10.1016/j.stemcr.2018.01.002 (PMC5830958; doi:10.1016/j.stemcr.2018.01.002)
Supplement: Document S2. Article plus Supplemental Information [file mmc3.pdf]

Stem Cell Reports
Article
OPEN ACCESS

Comparison of Non-human Primate versus Human Induced Pluripotent Stem Cell-Derived Cardiomyocytes for Treatment of Myocardial Infarction

Xin Zhao,[1,2,7] Haodong Chen,[1,2,7] Dan Xiao,[1,2,7] Huaxiao Yang,[1,2] Ilanit Itzhaki,[1,2] Xulei Qin,[1,2] Tony Chour,[1,2] Aitor Aguirre,[3] Kim Lehmann,[3] Youngkyun Kim,[1,2] Praveen Shukla,[1,2] Alexandra Holmström,[1,2] Joe Z. Zhang,[1,2] Yan Zhuge,[1,2] Babacar C. Ndoye,[1,2] Mingtao Zhao,[1,2] Evgenios Neofytou,[1,2] Wolfram-Hubertus Zimmermann,[4,5] Mohit Jain,[3] and Joseph C. Wu[1,2,6,*]
[1]Stanford Cardiovascular Institute, Stanford, CA 94305-5454, USA
[2]Institute for Stem Cell Biology and Regenerative Medicine, Stanford, CA 94305, USA
[3]Departments of Medicine and Pharmacology, University of California, San Diego, CA 92093, USA
[4]Institute of Pharmacology and Toxicology, University Medical Center Goettingen, 37075 Goettingen, Germany
[5]DZHK (German Center for Cardiovascular Research), Partner Site, Goettingen, Germany
[6]Department of Medicine, Division of Cardiology, Stanford University School of Medicine, Stanford, CA 94305, USA
[7]Co-first author
*Correspondence: joewu@stanford.edu
https://doi.org/10.1016/j.stemcr.2018.01.002

SUMMARY

Non-human primates (NHPs) can serve as a human-like model to study cell therapy using induced pluripotent stem cell-derived cardiomyocytes (iPSC-CMs). However, whether the efficacy of NHP and human iPSC-CMs is mechanistically similar remains unknown. To examine this, RNU rats received intramyocardial injection of 1×10^7 NHP or human iPSC-CMs or the same number of respective fibroblasts or PBS control (n = 9–14/group) at 4 days after 60-min coronary artery occlusion-reperfusion. Cardiac function and left ventricular remodeling were similarly improved in both iPSC-CM-treated groups. To mimic the ischemic environment in the infarcted heart, both cultured NHP and human iPSC-CMs underwent 24-hr hypoxia *in vitro*. Both cells and media were collected, and similarities in transcriptomic as well as metabolomic profiles were noted between both groups. In conclusion, both NHP and human iPSC-CMs confer similar cardioprotection in a rodent myocardial infarction model through relatively similar mechanisms via promotion of cell survival, angiogenesis, and inhibition of hypertrophy and fibrosis.

INTRODUCTION

With the recent progress in effective preventions and advanced treatments, mortality rates for heart failure and acute myocardial infarction (MI) have declined. However, 1 in every 4 deaths is still caused by heart disease in the United States (Vos et al., 2012). Despite early pharmacologic and medical device intervention to reduce myocardial loss after infarction, viable cardiomyocytes (CMs) within the ischemic zone and adjacent areas still experience increased workload and metabolic stress, resulting in heart failure with further cell loss and pathologic myocardial remodeling (Pfeffer and Braunwald, 1990).

In recent years, stem cell-based regenerative therapy has entered preclinical and clinical trials to repair the infarcted heart (Laflamme et al., 2007; Mangi et al., 2003; Menasché et al., 2015, 2018). The unique properties of induced pluripotent stem cells (iPSCs), including their ability for self-renewal, provide a sufficient resource to generate differentiated cells for transplantation. Although preclinical studies have shown that CMs differentiated from iPSCs can improve cardiac function and attenuate myocardial remodeling after MI (Carpenter et al., 2012; Citro et al., 2014), the field is still mainly at the preclinical trial stage due to technical and practical reasons (Neofytou et al., 2015). It is important to examine the safety and immunogenicity further in a human-like animal model, such as non-human primates (NHPs). NHPs have phylogenetic proximity to humans and can be an ideal model for preclinical autologous/allogeneic transplantation studies. However, an essential step before NHP trials is to prove the equivalence of functional efficacies and underlying therapeutic mechanisms between NHP iPSC-CMs and human iPSC-CMs. This step is necessary to translate NHP animal results to clinical studies.

In the present study, we injected NHP iPSC-CMs or human iPSC-CMs intramyocardially in a subacute MI rat model. Four weeks after cell injection, similar improvements in left ventricular (LV) function and myocardial remodeling were noted in both cell-treated groups, but not in the control groups treated with PBS or NHP fibroblasts or human fibroblasts. We further examined the effects of oxygen depletion on NHP iPSC-CMs and human iPSC-CMs *in vitro*, which is a condition the cells first encounter after injection into the heart. The majority of the changes in transcriptomic and metabolomic profile of NHP and human iPSC-CMs were similar, with some species-specific differences.

 © 2018 The Authors.
This is an open access article under the CC BY license (http://creativecommons.org/licenses/by/4.0/).

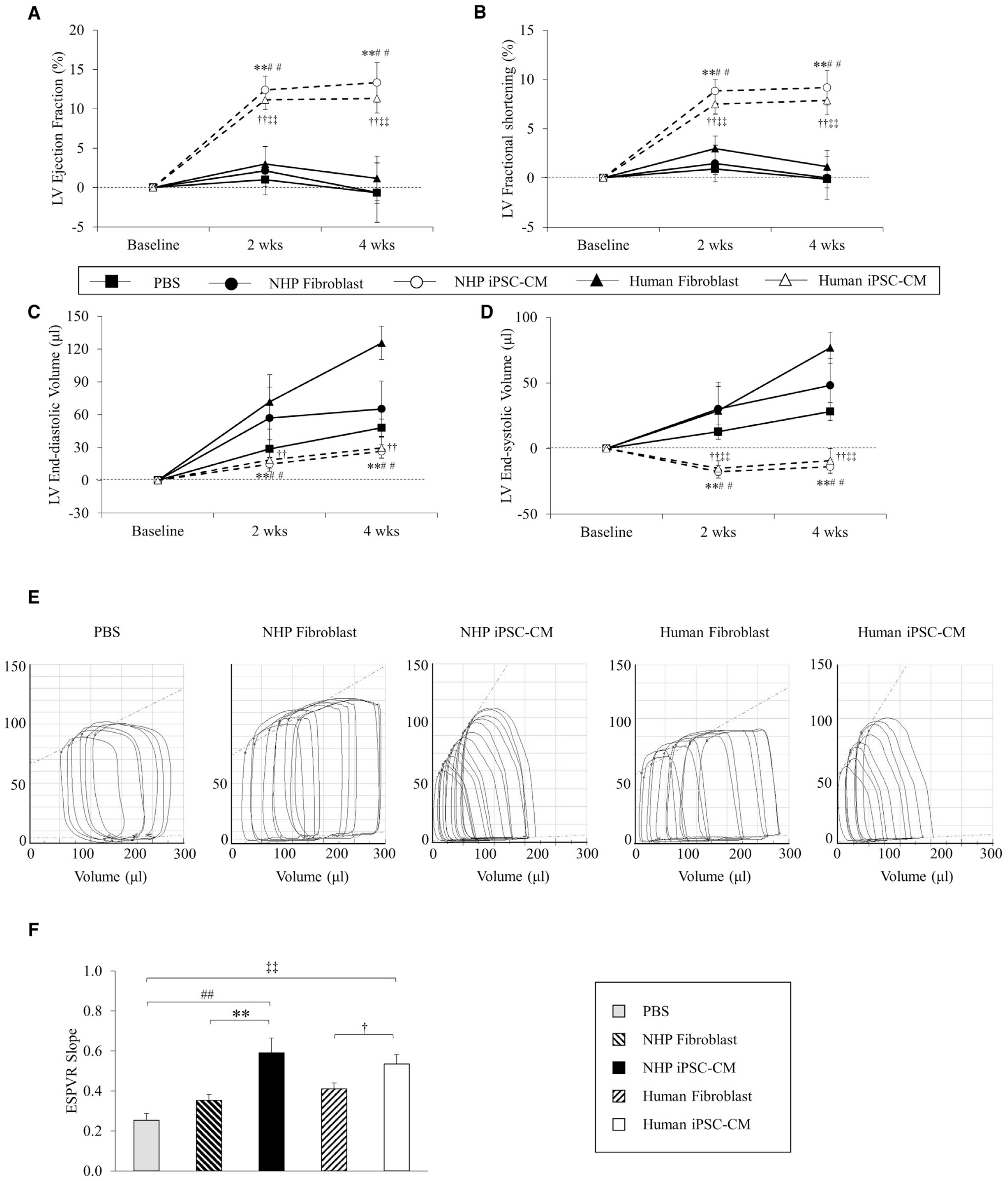

Figure 1. Cardiac Function Assessment after Cell Injection

Echocardiography was monitored at 1 day before cell injection, and 2 weeks and 4 weeks after cell injection.

(A and B) LV ejection fraction (A) and fractional shortening (B) were improved significantly in both NHP and human iPSC-CM groups compared with the PBS group.

(legend continued on next page)

RESULTS

CM Differentiation from NHP iPSCs and Human iPSCs

Thirty days after differentiation, silencing of reprogramming factors were confirmed by qPCR in both NHP and human iPSC-CMs (data not shown). Immunohistological staining showed that both NHP and human iPSC-CMs expressed key cardiac markers such as cardiac troponin T (cTnT) and α-actinin. In addition, the sarcomere structure was clear and well aligned in both NHP and human iPSC-CMs (Figure S1A), confirming successful differentiation. Both NHP and human iPSC-CMs showed cardiac-specific electrophysiological phenotype by displaying the three subtypes of cardiac action potential: ventricular-like, atrial-like, and nodal-like (Figure S1B and Table S1).

Left Ventricular Function Change after Cell Injection

One day before cell injection, post-MI cardiac function was assessed by echocardiography (Table S2). The rats were divided into five groups to receive PBS, NHP fibroblasts, NHP iPSC-CMs, human fibroblasts, or human iPSC-CMs. Four weeks after injection, the left ventricular ejection fraction (LVEF) did not change significantly in the PBS group (−0.7% ± 1.4%) or fibroblast control groups (−0.6% ± 3.8% in NHP fibroblast group; +1.2% ± 2.8% in human fibroblast group) but was increased significantly in both the NHP iPSC-CM group (+13.3% ± 1.9%) and the human iPSC-CM group (+11.3% ± 2.6%) ($p < 0.01$, Figure 1A). Similarly, fractional shortening was also significantly improved in both cell-treated groups (+9.2% ± 1.5% in NHP iPSC-CM group and +7.9% ± 1.8% in human iPSC-CM group versus −0.1% ± 0.9% in PBS group, 0.0% ± 2.2% in NHP fibroblast group, and +1.2% ± 1.6% in human fibroblast group; $p < 0.01$, Figure 1B). The improved contractility was accompanied by the amelioration of LV chamber dilatation at both phases of end-diastole (+27 ± 5.9 μL in NHP iPSC-CM group and +24 ± 7.9 μL in human iPSC-CM group versus +48 ± 7.9 μL in PBS group, +65 ± 25 μL in NHP fibroblast group, and +126 ± 15 μL in human fibroblast group; $p < 0.05$, Figure 1C) and end-systole (−14 ± 5.4 μL in NHP iPSC-CM group and −9.3 ± 9.4 μL in human iPSC-CM group versus +28 ± 6.7 μL in PBS group, +48 ± 21 μL in NHP fibroblast group, and +77 ± 12 μL in human fibroblast group; $p < 0.05$, Figure 1D) compared with the baseline.

Hemodynamic analysis further confirmed the improvement of LV systolic and diastolic function in both NHP and human iPSC-CM-treated groups. With a similar heart rate, LV systolic pressure, and LV systemic pressure, we found that the maximum dP/dt was significantly higher in both NHP iPSC-CM (7,497 ± 316 mmHg/s, n = 11) and human iPSC-CM groups (7,991 ± 434 mmHg/s, n = 12) compared with the NHP fibroblast (5,835 ± 242 mmHg/s, n = 9), human fibroblast (6,385 ± 259 mmHg/s, n = 9), and PBS control groups (6,628 ± 103 mmHg/s, n = 7; $p < 0.05$) (Table S3). The slope of end-systolic pressure-volume relationship generated by inferior vena cava occlusion suggests a similarly increased contractility in both NHP iPSC-CM and human iPSC-CM groups compared with both NHP and human fibroblast-treated groups as well as the PBS-treated group ($p < 0.01$, Figures 1E and 1F). The LV minimum dP/dt was lower in both NHP iPSC-CM (−6,393 ± 385 mmHg/s) and human iPSC-CM groups (−6,740 ± 499 mmHg/s) compared with NHP fibroblast (−5,357 ± 333 mmHg/s) and human fibroblast groups (−5,633 ± 196 mmHg/s), as well as the PBS group (−5,327 ± 101 mmHg) ($p < 0.05$, Table S3). The end-diastolic pressure and time constant, tau, in both cell-treated groups were similar and were significantly decreased compared with both fibroblast groups or PBS group ($p < 0.05$, Table S3).

Reduced Infarct Size and Increased Viable Myocardium in Cell-Treated Groups

Four weeks after cell transplantation, graft survival was identified in the ischemic zone at different sections of the left ventricle in both NHP and human iPSC-CM groups as assessed by cTnT and human specific mitochondria double staining (Figures 2A and 2B). The graft resulted in an increased amount of viable tissue within the infarct zone in both NHP iPSC-CM (39% ± 3.2%) and human iPSC-CM groups (41% ± 3.2%) compared with the NHP

(C) LV end-diastolic volume was markedly dilated in the PBS control, NHP fibroblast, and human fibroblast groups compared with both NHP and human iPSC-CM groups.
(D) LV end-systolic volume was increased in the PBS control, NHP fibroblast, and human fibroblast groups but decreased in both NHP and human iPSC-CM groups (n = 14 independent experiments). Hemodynamics was analyzed using pressure-volume Millar catheter.
(E) Examples of changes in pressure-volume relationships during inferior vena cava occlusion.
(F) Slope of end-systolic pressure-volume relationship (ESPVR) showed significantly preserved cardiac contractility in both NHP and human iPSC-CM groups.
PBS, n = 7; NHP fibroblast, n = 9; NHP iPSC-CM, n = 11; human fibroblast, n = 9; human iPSC-CM, n = 12 independent experiments. Data are presented as mean ± SEM. $^{**}p < 0.01$, NHP iPSC-CM versus NHP fibroblast; $^{\#\#}p < 0.01$, NHP iPSC-CM versus PBS; $^{\dagger}p < 0.05$, $^{\dagger\dagger}p < 0.01$, human iPSC-CM versus human fibroblast; $^{\ddagger\ddagger}p < 0.01$, human iPSC-CM versus PBS; by one-way ANOVA.

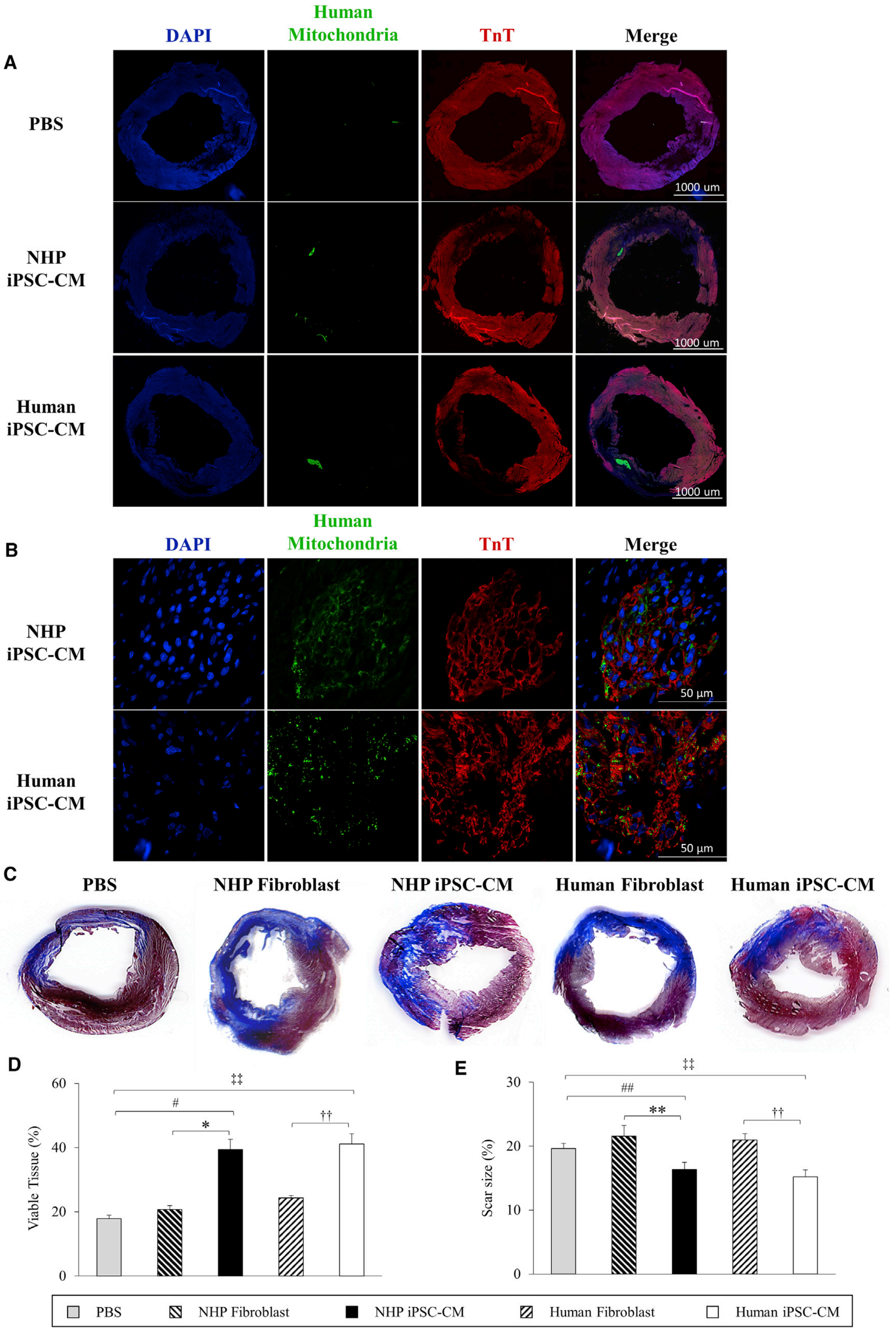

(legend on next page)

fibroblast (21% ± 1.2%) and human fibroblast groups (24% ± 0.7%), as well as the PBS group (18% ± 1.1%; $p < 0.05$) (Figures 2C and 2D). Additionally, the infarct size was significantly smaller in the groups treated with NHP iPSC-CMs (16% ± 1.1%) and human iPSC-CMs (15% ± 1.1%) than that treated with NHP fibroblast (22% ± 1.7%), human fibroblast (21% ± 1.7%), and PBS (20% ± 0.8%; $p < 0.05$) (Figure 2E).

Attenuated Myocardial Remodeling with Increased Angiogenesis after CM Transplantation

With the improvement in LV function and more viable tissue in cell-treated groups, the extent of myocardial remodeling was further assessed at both the border zone (BZ) and remote zone (RZ) of the infarct site according to the histology approach. The endogenous rat cardiomyocytes were significantly smaller in the groups treated with NHP iPSC-CMs (393 ± 36 μm^2 at BZ and 343 ± 21 μm^2 at RZ) or human iPSC-CMs (381 ± 29 μm^2 at BZ and 343 ± 21 μm^2 at RZ) compared with PBS group (579 ± 69 μm^2 at BZ and 505 ± 40 μm^2 at RZ), NHP fibroblast group (552 ± 25 μm^2 at BZ and 413 ± 25 μm^2 at RZ), and human fibroblast group (524 ± 31 μm^2 at BZ and 391 ± 24 μm^2 at RZ), suggesting a more limited hypertrophy ($p < 0.05$, Figures 3A and 3B). A similar reduction of cardiac fibrosis was noted in both cell-treated groups (16% ± 0.8% at BZ and 11% ± 0.4% at RZ in the NHP iPSC-CM group, 16% ± 1.0% at BZ and 12% ± 0.5% at RZ in the human iPSC-CM group), but not in control groups (20% ± 0.9% at BZ and 15% ± 0.9% at RZ in PBS group, 18% ± 1.2% at BZ and 15% ± 1.0% at RZ in NHP fibroblast group, 20% ± 1.1% at BZ and 15% ± 0.9% at RZ in human fibroblast group) ($p < 0.05$; Figures 3C and 3D). In addition to the attenuation of the myocardial remodeling, the capillary density in both NHP iPSC-CM (1,672 ± 110/mm^2 at BZ and 2,387 ± 205/mm^2 at RZ) and human iPSC-CM groups (1,795 ± 130/mm^2 at BZ and 2,475 ± 170/mm^2 at RZ) were significantly higher than those in the NHP fibroblast group (1,199 ± 88/mm^2 at BZ and 1,840 ± 109/mm^2 at RZ), human fibroblast group (1,158 ± 120/mm^2 at BZ and 1,889 ± 161/mm^2 at RZ), and PBS group (1,163 ± 109/mm^2 at BZ and 1,665 ± 136/mm^2 at RZ) ($p < 0.01$; Figures 3E and 3F), indicating a possible increase in angiogenesis and a reduction in cellular remodeling/hypertrophy.

Gene Profile Change in Response to Oxygen Depletion *In Vitro*

When transplanted, exogenous cells initially encounter the hypoxic environment of the ischemic heart. To study their responses to hypoxic condition, we next subjected cultured NHP iPSC-CMs and human iPSC-CMs to oxygen depletion for 24 hr. RNA sequencing (RNA-seq) revealed a total of 444 genes upregulated and 161 genes downregulated in NHP iPSC-CMs compared with 288 genes upregulated and 97 genes downregulated in human iPSC-CMs. Among these genes, only 159 were commonly upregulated and 28 commonly downregulated in the two groups (Table S4). Further analysis indicates that these genes are responsible for regulation of glucose metabolism and post-translational modification, among other processes (Figure 4A). However, when evaluating the functions of differentially expressed genes in the two species, more common pathways were identified with the functions of promoting cell viability, cell survival, and glycolysis, as well as in inhibiting cardiac hypertrophy and fibrosis (Figure 5A). Interestingly, some pathways that are regulated toward opposite directions between the two groups are more related to the fate of connective tissue cells, fibroblasts, and endothelial cells (Figure 5B).

Using Ingenuity Pathway Analysis (IPA), genes involved in shared pathways regulating cell survival, angiogenesis (Figures S2A and S2B), hypertrophy, and fibrosis (Figures S3A and S3B) are dimensionally divided into nucleus, cytosol, membrane, and extracellular space according to the spatial distribution of their encoded proteins. Interestingly, the vascular endothelial growth factor (VEGF) was significantly elevated in both groups (4.6-fold increase in NHP iPSC-CMs and 2.7-fold in human iPSC-CMs; $p < 0.01$, Figure 4B) and was noted in all four protective pathways. More importantly, this protein was located in the extracellular space according to IPA as a secreted factor. The media collected from the cultured CMs further proved that VEGF was indeed secreted from both types of

Figure 2. Graft and Scar Size Assessment at 4 Weeks after Myocardial Infarction

(A) Examples of viable graft in the ischemic zone. Both NHP and human iPSC-CMs were identified with non-specific cardiac troponin T and human specific mitochondria at 4× magnification.

(B) Viable grafts in both groups of iPSC-CM-treated hearts were observed under 63× magnification.

(C) Representative pictures of infarcted left ventricle with trichrome staining. PBS and fibroblast control groups showed more transmural infarction than iPSC-CM-treated groups, which had more viable myocardium.

(D) The amount of viable myocardium was quantified within the ischemic zone and presented as a percentage of the area of ischemic zone.

(E) Scar size, presented as a percentage of fibrotic tissue to whole myocardial area, was compared among PBS and cell-treated groups.

PBS, n = 5; NHP fibroblast, n = 6; NHP iPSC-CM, n = 6; human fibroblast, n = 6; human iPSC-CM, n = 6 independent experiments. Data are presented as mean ± SEM. *$p < 0.05$, **$p < 0.01$, NHP iPSC-CM versus NHP fibroblast; $^{\#}p < 0.05$, $^{\#\#}p < 0.01$, NHP iPSC-CM versus PBS; $^{\dagger\dagger}p < 0.01$, human iPSC-CM versus human fibroblast; $^{\ddagger\ddagger}p < 0.01$, human iPSC-CM versus PBS.

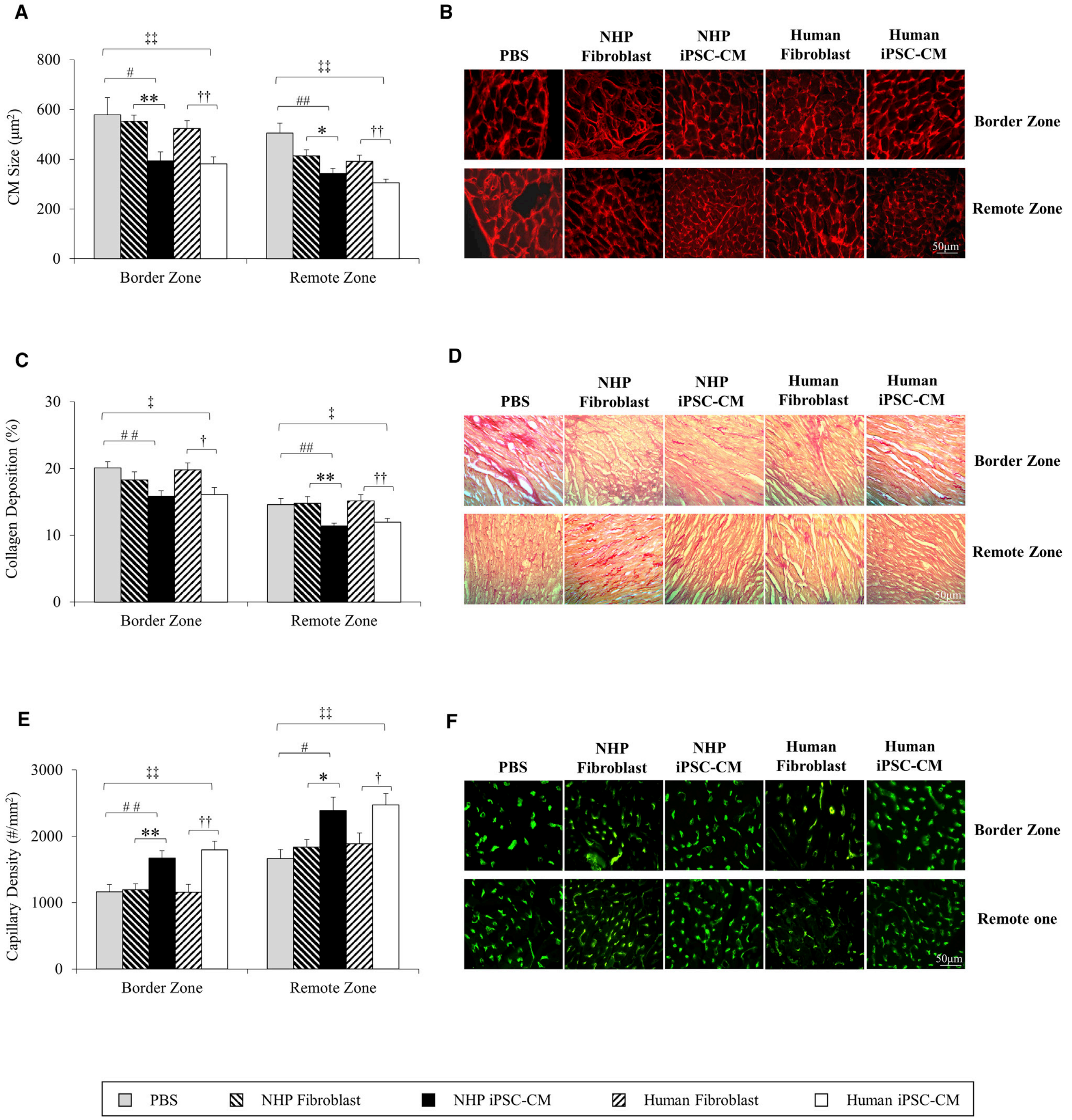

Figure 3. Myocardial Remodeling Was Compared at Histological Level at Border Zone and Remote Zone to the Infarct

(A) Cardiomyocyte size was compared among PBS, NHP fibroblast, NHP iPSC-CM, human fibroblast, and human iPSC-CM groups.

(B) Examples of wheat germ agglutinin staining showing the size of endogenous mouse cardiomyocytes of the three groups.

(C) Fibrosis was quantified as the percentage of interstitial collagen deposition.

(D) Representative images of Picro-Sirius red staining showing interstitial fibrosis of the three groups.

(E) Capillary density was quantified as absolute number of capillaries per unit area.

(F) Representative CD144 staining showing capillaries.

PBS, n = 5; NHP fibroblast, n = 6; NHP iPSC-CM, n = 6; human fibroblast, n = 6; human iPSC-CM, n = 6 independent experiments. Data are presented as mean ± SEM. $^{*}p < 0.05$, $^{**}p < 0.01$, NHP iPSC-CM versus NHP fibroblast; $^{\#}p < 0.05$, $^{\#\#}p < 0.01$, NHP iPSC-CM versus PBS; $^{\dagger}p < 0.05$, $^{\dagger\dagger}p < 0.01$, human iPSC-CM versus human fibroblast; $^{\ddagger}p < 0.05$, $^{\ddagger\ddagger}p < 0.01$ human iPSC-CM versus PBS.

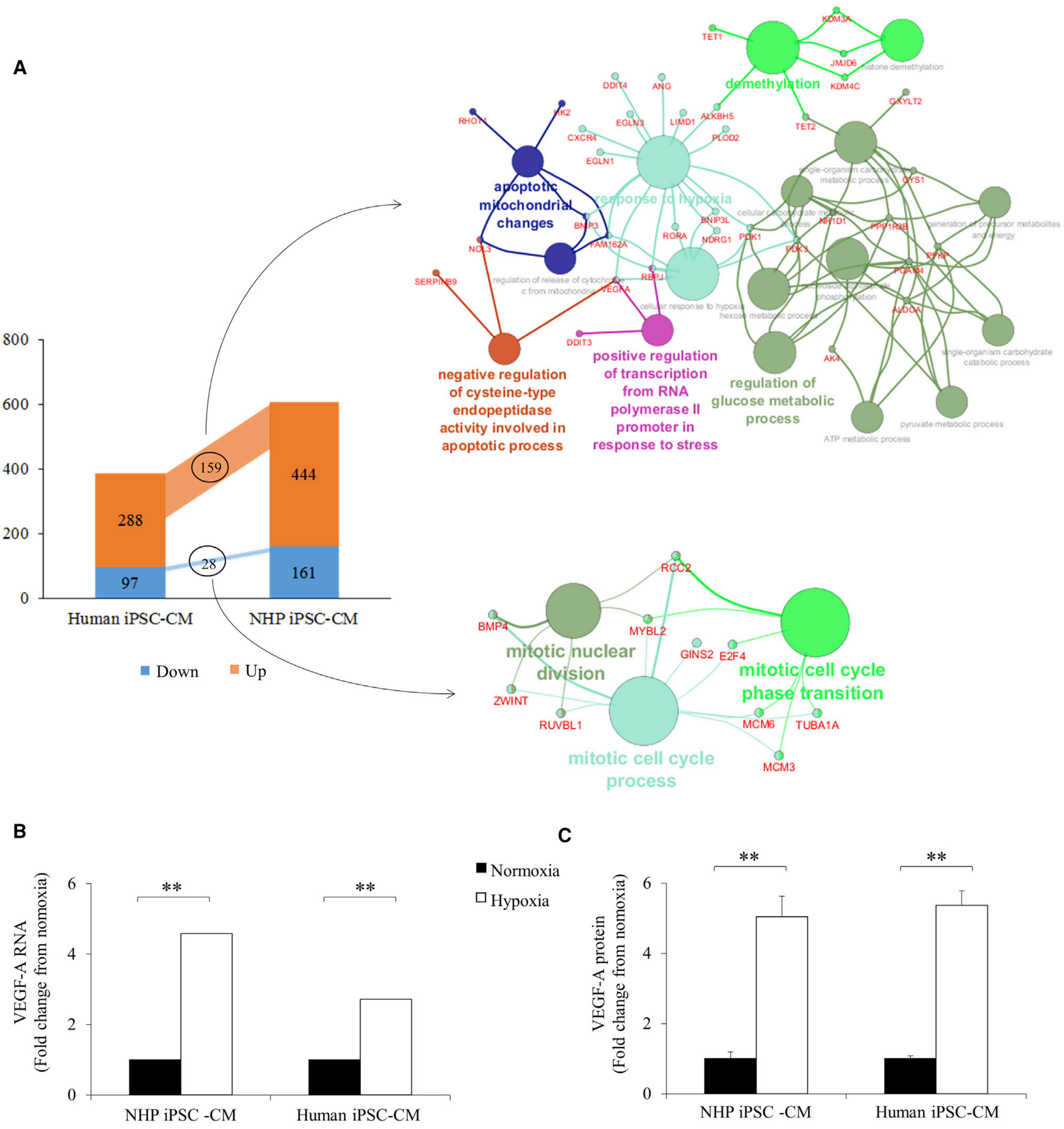

Figure 4. RNA-Seq Analysis of Both NHP and Human iPSC-CMs in Response to 24-hr Oxygen Depletion
(A) The numbers of significantly regulated genes ($p < 0.05$) and the significant biological function of the common genes in both groups are listed.
(B) Fold change of VEGF gene in hypoxic iPSC-CMs from both species.
(C) Fold change of VEGF in culture medium using proteomic angiogenesis assay.
n = 3 independent experiments. Data are presented as mean ± SEM. **$p < 0.01$.

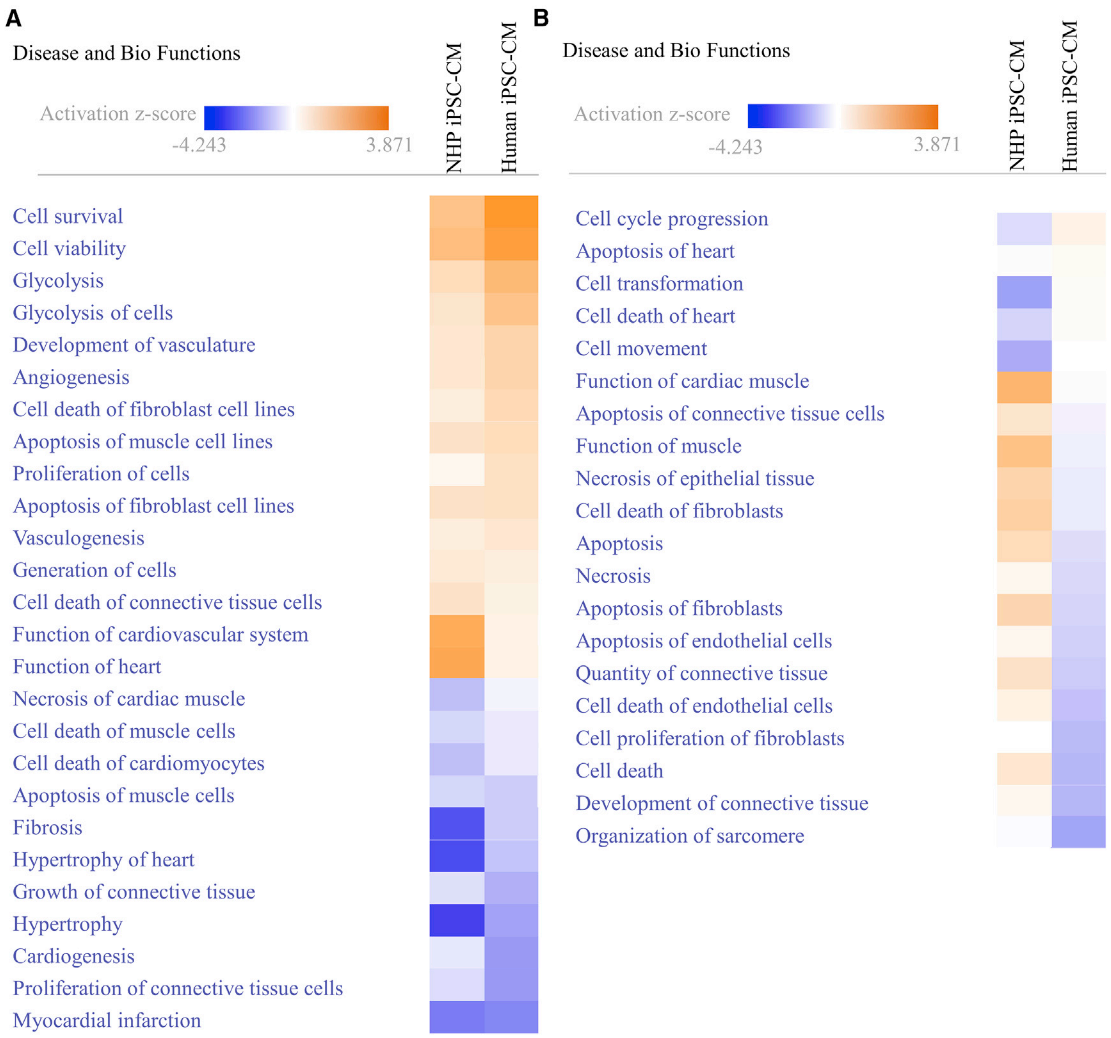

Figure 5. Comparison of Biological Function Revealed by RNA-Seq Analysis between NHP and Human iPSC-CMs after 24-hr Hypoxia
(A) Common biological functions of the genes.
(B) Biological functions that are regulated toward opposite directions.
n = 3 independent experiments.

iPSC-CMs after oxygen depletion (5.0 ± 0.6-fold increase in NHP iPSC-CMs and 5.4 ± 0.4-fold in human iPSC-CMs; $p < 0.01$, Figure 4C).

To understand how these differentially expressed genes were regulated, we next studied the transcription factors that bind to the conserved regions of these genes using the online Database for Annotation, Visualization and Integrated Discovery (DAVID) v6.8. We found that among the top enriched transcription factors, 25 of them, including hepatic leukemia factor and insulin-like growth factor 1 (IGF-1), were shared by both species. Other transcription factors distinctly appeared in one of the groups, such as FOXO1 and STAT1 in NHP iPSC-CMs versus FOXO3 and ATF6 in human iPSC-CMs (Table S5).

Metabolic Paracrine Actions from iPSC-CMs after Oxygen Depletion

To elucidate how paracrine factors secreted from iPSC-CMs affect the microenvironment through metabolic regulation, we collected culture media from NHP and human iPSC-CMs at 24 hr after oxygen depletion for metabolomics analysis. A heatmap was generated based on metabolomics data that compared the fold change of the number of metabolites detected under the hypoxic condition with those under the normoxic condition (Figure 6A). The pattern in the heatmap shows that the signatures of both species are largely similar except for some local differences. A total of 318 variables/metabolites were retained in the volcano plot, among which 70 variables in NHP

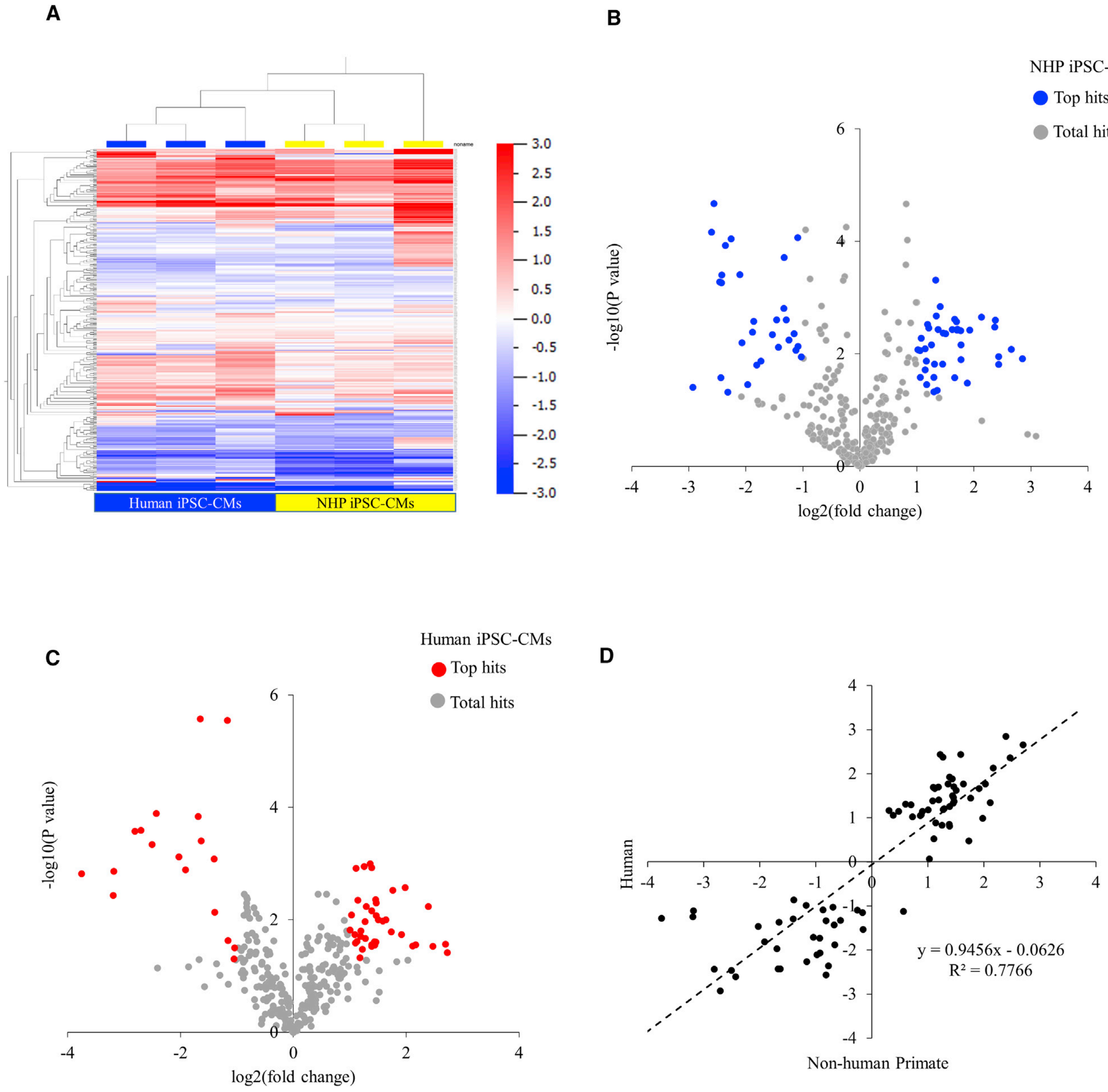

Figure 6. Metabolomic Analysis of the Culture Media in Response to 24-hr Hypoxia

(A) Heatmap generated from metabolomic analysis. Relative expression values (log ratios versus normoxic condition) were used.

(B) Volcano plot of the total and top regulated ($p < 0.05$) metabolites seen in NHP iPSC-CMs.

(C) Volcano plot of the total and top regulated ($p < 0.05$) metabolites seen in human iPSC-CMs.

(D) Comparison of the metabolites changed more than 2-fold between the two species.

n = 3 independent experiments.

iPSC-CMs (Figure 6B) and 58 in human iPSC-CMs (Figure 6C) reached statistical difference ($p < 0.05$). When metabolites that were changed over 2-fold from normoxic condition between the two groups were plotted, the slope of the regression line was 0.95, with a coefficient of determination R^2 of 0.78, suggesting the majority of the changes in metabolites were proportional between NHP and human iPSC-CMs (Figure 6D). Both groups were found to be involved in regulating ketogenesis, ketolysis, and methylglyoxal-related metabolism (Figure S4A). However, metabolites from NHP iPSC-CMs were more involved in glycogenolysis, whereas the metabolites from human

iPSC-CMs were related to lactose degradation and citric acid cycle (Figures S4B and S4C).

DISCUSSION

Our study compared the similarities and differences between NHP iPSC-CMs and human iPSC-CMs for treatment of subacute MI in a rodent model. For clinical relevance, rather than using permanent coronary artery ligation as used by previous studies (Citro et al., 2014; Lepperhof et al., 2014; Ong et al., 2015), we utilized the ischemia-reperfusion model to simulate MI patients with re-established blood supply after intervention. In addition, instead of acute treatment within 24 hr after MI (Carpenter et al., 2012; Lepperhof et al., 2014; Ong et al., 2015), we injected cells at day 4 post infarction, which is the subacute stage of MI, to simulate the delay for iPSC-CM match and preparation for cell transplantation. After cell injection, both NHP and human iPSC-CM groups showed similar improvement in LV contractility at 2 weeks post transplantation, and this improvement was preserved at week 4. Furthermore, both echocardiography and hemodynamic analysis demonstrate the improvement in both systolic and diastolic function as shown by LVEF and tau. To prove that such an effect is specific to iPSC-CMs, we analyzed a separate negative cohort with functionally unrelated NHP or human fibroblasts. Similar to previous studies (Kolossov et al., 2006; Yeghiazarians et al., 2012), during the 4-week period after cell delivery, fibroblast-treated groups did not alter cardiac function, and severe LV chamber dilatation was observed at the end of the 4-week period. It is worth noting that while the end-diastolic volume continuously increased after cell transplantation in the control groups, the end-systolic volume was markedly reduced in both iPSC-CM-treated groups. This continual dilatation and remodeling in end-diastolic volume may represent a compensatory mechanism in response to increased wall stress (Grossman, 1980). In previous studies using iPSC-CMs from different species, the changes in LV function varied from study to study (Citro et al., 2014; Wu et al., 2016). In our current study, both NHP and human iPSC-CMs showed similar potency in improving LV function and attenuation in chamber dilatation.

This similarity in functional improvement was accompanied by similar changes at the histological levels in both NHP and human iPSC-CMs. We noted that within the ischemic zone, the total viable tissues (composed of grafts and host myocardium) were present to a similar extent between the two iPSC-CM-treated groups. In addition to the increased viable tissue and reduced scar size, we noted a smaller cardiomyocyte size and less interstitial fibrotic tissue in both iPSC-CM-treated groups at both BZ and RZ. This indicates a similar attenuation in remodeling with less hypertrophy and less reactive fibrosis. Moreover, consistent with previous studies showing angiogenesis in the iPSC-CM-treated MI hearts (Lepperhof et al., 2014; Ye et al., 2014), we also found that the capillary density was similarly increased in both iPSC-CM-treated groups at the BZ and RZ. Although the total amount of grafts was difficult to quantify accurately at 4 weeks after cell transplantation, we observed that a small amount of grafts exist at different sections of the LV wall in both iPSC-CM-treated groups. They presented as either free cells or clusters at the ischemic zone and BZ, but there was no sign of remuscularization or contact between engraftment and host myocardium as reported in other studies (Chong et al., 2014; Shiba et al., 2016). Therefore, the improved function was not directly due to the engraftment in the ischemic zone. Comparing the size of the grafts with the whole LV wall, it is reasonable to infer that paracrine effects of iPSC-CMs play a pivotal role in maintaining cardiac function and attenuating myocardial remodeling. Indeed, numerous studies have shown that adult stem cells and their derived cells affect the microenvironment via paracrine actions (Mirotsou et al., 2007; Ong et al., 2015; Ye et al., 2015).

To further understand post-transplantation response of the graft to the host ischemic myocardium, we simulated the oxygen-depletion condition the grafts encountered *in vivo*, and compared the transcriptomic and metabolomic profiles of both iPSC-CMs *in vitro*. Given the species difference, it was not surprising to see certain differences in the iPSC-CMs at the molecular level. Among all genes that were significantly up- or downregulated, RNA-seq revealed only a small portion of shared genes between NHP and human iPSC-CMs. Despite few overlapping genes, we noted that many biological functions were regulated similarly toward the same direction. This finding suggests that in addition to using some of the shared genes, NHP and human iPSC-CMs utilized different sets of genes to achieve the same *in vivo* outcomes. For instance, along with a similar increase in capillary density *in vivo*, the RNA-seq showed a comparable upregulation in VEGF and angiogenin. However, IGF receptor (Bid et al., 2012) was significantly upregulated only in NHP iPSC-CMs, whereas platelet-derived growth factor (Moriya et al., 2014) was upregulated more in human iPSC-CMs. These phenomena also applied to the genes promoting cell survival and inhibiting fibrosis and hypertrophy (Figures S2 and S3), as well as transcription factors that are involved in cardioprotection (Table S5). Although *in vivo* analysis showed that the protection against MI was similar between the two groups, RNA-seq and pathway analysis revealed that fewer genes were involved in these related pathways in human iPSC-CMs compared with NHP iPSC-CMs. Transcription factors

that control these genes were changed more dramatically in NHP iPSC-CMs (Table S5).

We also found that some pathways were differentially activated between NHP and human iPSC-CMs, indicating a species-specific response to hypoxia. Interestingly, VEGF was found to be upregulated in both NHP and human iPSC-CMs. VEGF is implicated in all four cardioprotective biological functions, promoting cell survival and angiogenesis as well as inhibiting hypertrophy and fibrosis. Based on IPA, the VEGF gene encodes a protein that will be eventually secreted to extracellular space. Indeed, proteomic analysis of the culture media after oxygen depletion also showed an increased VEGF level. VEGF has been demonstrated by several studies to confer benefits to the ischemic heart (Byrne et al., 2005; Hoeben et al., 2004; Park et al., 2009; Xu et al., 2011). Although gene therapy for MI with VEGF failed in clinical trials (Kastrup et al., 2005; Stewart et al., 2009), its contribution to cardioprotection cannot be excluded.

In addition to VEGF, we also found other hypoxia-induced differentially expressed genes shared by both NHP and human iPSC-CMs. Jumonji domain-containing protein 6 (JMJD6) is an arginine demethylase known to demethylate histone H3 at arginine 2 and histone H4 at arginine 3 (Chang et al., 2007) and found to be upregulated in hypoxia-treated cardiomyocytes in our RNA-seq data. Previous reports have shown that JMJD6 regulates RNA splicing through modification of splicing factor U2 small nuclear ribonucleoprotein auxiliary factor 65-kDa subunit (Webby et al., 2009) and is required in endothelial cells to regulate the splicing of VEGF receptor as well as angiogenic sprouting (Boeckel et al., 2011). Despite its expression in cardiomyocytes as shown by immunostaining (Human Protein Atlas, www.proteinatlas.org), the specific role of JMJD6 in cardiomyocytes under hypoxia condition is not known and may require additional investigation. Moreover, we found some genes to be downregulated by hypoxia in both NHP and human iPSC-CMs, including MYB Proto-Oncogene Like 2 (MYBL2), also known as B-MYB. MYBL2 is a cyclin-dependent kinase and has been shown to activate genes during the S phase of the cell cycle (Joaquin and Watson, 2003). It is unclear how the downregulation of MYBL2 facilitates the ischemic response of cardiomyocytes, but downregulation of the cell cycle has been shown to protect cells from DNA damage or ischemia-induced cell death (Nowsheen and Yang, 2012). Taken together, our RNA-seq data suggest common pathways for both NHP and humna iPSC-CMs in response to ischemia.

The metabolomic profile of the two types of iPSC-CMs in response to oxygen depletion was another parameter we examined in comparing the two species. Unlike changes in the transcriptomic profile in which a small number of common genes were shared between the two species, the changes in secreted metabolites were much more alike. This similarity existed not only in the direction of the changes but also in the extent of the changes of individual metabolites (Figure 6D). In examining the top changed metabolic pathways, we noted, along with certain similarities between the two species, that there were more regulated pathways unique to one species (Figures S4B and S4C).

One potential limitation of our study is the absence of a large animal model due to prohibitive costs (e.g., transplantation of NHP or human iPSC-CMs into NHP). Allogeneic transplantation with rodent iPSC-CM is also an option. However, current protocols do not allow for the generation of rat or mouse iPSC-CMs at a sufficient amount and purity level (Liao et al., 2009; Liu et al., 2013; Merkl et al., 2013; Takenaka-Ninagawa et al., 2014; Yamaguchi et al., 2014). Nevertheless, our observation of comparable improvement with either NHP or human iPSC-CMs in a xenogeneic rodent model allows an objective comparison between these two species. Using the current model we could normalize confounding factors such as rejection and arrhythmia, thus creating no bias against either NHP or human iPSC-CM transplantation. Finally, our experiments provide additional information regarding functional, transcriptomic, and metabolomic changes between NHP and human iPSC-CMs that may lead to better preclinical study designs in the future.

Our study provides the cross-species comparison of iPSC-CMs for the treatment of MI in a xenogeneic rodent model. Given the phylogenetic distance between human and rhesus (23.3 million years) (Kumar and Hedges, 1998), it is not surprising to observe differences and similarities when comparing NHP with human iPSC-CMs. Although the therapeutic efficacy *in vivo* and common pathways *in vitro* between the two types of iPSC-CMs were very similar, there were nevertheless differences at the transcriptomic and metabolomic levels. Future plans to study individual genes or metabolic pathways in NHP iPSC-CMs for predicting outcome in human iPSC-CMs should proceed with caution due to these differences, the exact significance of which awaits further investigation.

EXPERIMENTAL PROCEDURES

For an extended description of methods, please refer to Supplemental Experimental Procedures.

Culture and Maintenance of NHP and Human iPSCs

Fibroblasts from a rhesus macaque monkey and healthy male human donor were reprogrammed into iPSCs via Sendai virus vectors carrying the Yamanaka reprogramming factors (Oct4, Sox2, Nanog, and cMyc). Both NHP and human iPSCs were grown to

90% confluence (Lan et al., 2013; Sun et al., 2009) on Matrigel-coated plates (ES Qualified, BD Biosciences, San Diego, CA) using a chemically defined E8 medium as previously described (Chen et al., 2011). The medium was changed daily, and cells were passaged every 3–4 days with EDTA (Thermo Fisher Scientific, CA).

Cardiac Differentiation

Both NHP and human iPSCs were grown to 90% confluence and subsequently differentiated into beating cardiomyocytes as previously described (Ebert et al., 2014; Liang et al., 2016). In brief, on day 0 cells were supplemented with a basal medium (RPMI 1640 [Thermo Fisher Scientific] and 2% B27 supplement minus insulin [Thermo Fisher Scientific]), with the addition at a concentration of 2–8 μM of CHIR-99021 [Selleck Chemicals], a selective inhibitor of glycogen synthase kinase 3β, which activates the canonical Wnt signaling pathway. On day 2, the medium was replaced with basal medium without CHIR99021 supplementation. On day 3, 5 μM of IWR-1 [Selleck Chemicals], a Wnt antagonist, was added to basal medium for 2 days. On day 5 and every subsequent other day until harvest, the medium was replaced with fresh basal medium.

Cell Transplantation

Four days after reperfusion, animals were randomly grouped into five groups: (1) 1×10^7 NHP iPSC-CMs (n = 14), (2) 1×10^7 human iPSC-CMs (n = 14), (3) 1× PBS (n = 14), (4) 1×10^7 human fibroblast (n = 9), and (5) 1×10^7 NHP fibroblast (n = 9). The chest was reopened and cells or PBS were injected intramyocardially at 2–3 sites along the edge of the infarct zone with a total volume of 70 μL with a 28-gauge insulin syringe. Study protocols were approved by the Stanford Animal Research Committee. Animal care was provided in accordance with the Stanford University School of Medicine guidelines and policies for the use of laboratory animals.

Echocardiography

At 1 day before injection, and 2 and 4 weeks after injection, cardiac function was assessed using transthoracic echocardiography (Vevo 2100 Imaging System, VisualSonics). Animals were anesthetized with 2% inhaled isoflurane, and LV internal dimensions were measured in systole and diastole using leading-edge methods and guidelines of the American Society of Echocardiography (Sahn et al., 1978). LV systolic function was estimated using the Vevo Lab software for calculation.

In Vitro Oxygen Depletion Treatment

At 30 days after differentiation, 3×10^6 NHP iPSC-CMs and 3×10^6 human iPSC-CMs were collected and replated onto Matrigel-coated 6-well plates. Culture media were changed every 2 days for 5 days. On day 5, half of the plates were placed in an anaerobic pouch (GasPak EZ Anaerobe Pouch System, Fisher Scientific, MA) without changing culture media and the other half were left untouched. The pouch was sealed with an anaerobic indicator. The color of indicator changed to blue for the anaerobic conditions. Cells were incubated for another 24 hr before media from both hypoxic and normoxic iPSC-CMs were collected for proteomic VEGF analysis. Cells were collected for transcriptomic profile analysis.

RNA Sequencing

Kits used for RNA-seq were purchased from Thermo Fisher. Total RNA was mixed with ERCC RNA Spike-In Mix 1 and polyadenylated mRNA was isolated using the Dynabeads mRNA DIRECT Micro Purification Kit. A cDNA library was prepared using the Ion Total RNA-seq Kit v2 and sequenced by the Ion Proton using Ion PI Hi-Q Sequencing kit. Unaligned BAM files were generated by the Ion Torrent Suite 5.0.4 and converted into FASTQ files. The reads for three replicates for human or NHP iPSC-CMs, with or without oxygen depletion, were mapped to reference genome hg19 (human) or rheMac8 (NHP) using TopHat (v2.0.13), assembled with Cufflinks (v2.2.1), and differentially expressed genes induced by oxygen depletion treatment were identified by Cuffdiff (v2.2.1). Because the rhesus macaque genome is not well annotated, human genome (RefSeq Genes) and the rhesus macaque genome were aligned for annotation, resulting in the same gene IDs for both human and rhesus macaque. Molecular pathways and disease or toxicological functions of differentially expressed genes (false discovery rate <0.05, log ratio >1.5) were analyzed by IPA (Qiagen).

Statistical Analysis

Data are expressed as mean ± SEM. Statistical significance was determined using Student's t test or ANOVA test evaluations. $p < 0.05$ was taken as a minimal level of significance.

ACCESSION NUMBERS

The accession number for the raw and processed data from the RNA-seq experiments reported in this paper is GEO: GSE108676.

SUPPLEMENTAL INFORMATION

Supplemental Information includes Supplemental Experimental Procedures, four figures, and five tables and can be found with this article online at https://doi.org/10.1016/j.stemcr.2018.01.002.

AUTHOR CONTRIBUTIONS

X.Z. conceived the study, designed the experiments, performed *in vivo* cardiac function assessments and *in vitro* hypoxic assay, and wrote the manuscript. H.C. examined iPSC-CM purity, performed RNA-seq, analyzed metabolomic data, and wrote the manuscript. D.X. differentiated the cells, characterized the cell lines, performed and analyzed echocardiography, and wrote the manuscript. I.I. and P.S. performed electrophysiology. H.Y. analyzed and characterized the histological findings. X.Q. performed echocardiography. T.C., A.H., Y.Z., B.C.N., J.Z.Z., Y.K., and M.Z. differentiated and characterized the cell lines. A.A., K.L., and M.J. performed metabolomic analysis. E.N. supervised animal experiments, including surgeries and histology. W-H.Z. contributed to the experimental design. J.C.W. conceived the idea and provided experimental advice, manuscript writing, and funding support. All authors reviewed the manuscript.

ACKNOWLEDGMENTS

This work was supported by the California Institute for Regenerative Medicine (RT3-07798, TR3-05556, and DR2A-05394) and the

NIH R01 HL133272, R01 HL113006, and R01 HL132875 (J.C.W.), R03 HL133720-01 (M.J.), and K01 HL135464-01 (A.A.).

Received: March 17, 2017
Revised: January 3, 2018
Accepted: January 4, 2018
Published: February 1, 2018

REFERENCES

Bid, H.K., Zhan, J., Phelps, D.A., Kurmasheva, R.T., and Houghton, P.J. (2012). Potent inhibition of angiogenesis by the IGF-1 receptor-targeting antibody SCH717454 is reversed by IGF-2. Mol. Cancer Ther. *11*, 649–659.

Boeckel, J.N., Guarani, V., Koyanagi, M., Roexe, T., Lengeling, A., Schermuly, R.T., Gellert, P., Braun, T., Zeiher, A., and Dimmeler, S. (2011). Jumonji domain-containing protein 6 (Jmjd6) is required for angiogenic sprouting and regulates splicing of VEGF-receptor 1. Proc. Natl. Acad. Sci. USA *108*, 3276–3281.

Byrne, A.M., Bouchier-Hayes, D.J., and Harmey, J.H. (2005). Angiogenic and cell survival functions of vascular endothelial growth factor (VEGF). J. Cell Mol. Med. *9*, 777–794.

Carpenter, L., Carr, C., Yang, C.T., Stuckey, D.J., Clarke, K., and Watt, S.M. (2012). Efficient differentiation of human induced pluripotent stem cells generates cardiac cells that provide protection following myocardial infarction in the rat. Stem Cells Dev. *21*, 977–986.

Chang, B., Chen, Y., Zhao, Y., and Bruick, R.K. (2007). JMJD6 is a histone arginine demethylase. Science *318*, 444–447.

Chen, G., Gulbranson, D.R., Hou, Z., Bolin, J.M., Ruotti, V., Probasco, M.D., Smuga-Otto, K., Howden, S.E., Diol, N.R., Propson, N.E., et al. (2011). Chemically defined conditions for human iPSC derivation and culture. Nat. Methods *8*, 424–429.

Chong, J.J., Yang, X., Don, C.W., Minami, E., Liu, Y.W., Weyers, J.J., Mahoney, W.M., Van Biber, B., Cook, S.M., Palpant, N.J., et al. (2014). Human embryonic-stem-cell-derived cardiomyocytes regenerate non-human primate hearts. Nature *510*, 273–277.

Citro, L., Naidu, S., Hassan, F., Kuppusamy, M.L., Kuppusamy, P., Angelos, M.G., and Khan, M. (2014). Comparison of human induced pluripotent stem-cell derived cardiomyocytes with human mesenchymal stem cells following acute myocardial infarction. PLoS One *9*, e116281.

Ebert, A.D., Kodo, K., Liang, P., Wu, H., Huber, B.C., Riegler, J., Churko, J., Lee, J., de Almeida, P., Lan, F., et al. (2014). Characterization of the molecular mechanisms underlying increased ischemic damage in the aldehyde dehydrogenase 2 genetic polymorphism using a human induced pluripotent stem cell model system. Sci. Transl. Med. *6*, 255ra130.

Grossman, W. (1980). Cardiac hypertrophy: useful adaptation or pathologic process? Am. J. Med. *69*, 576–584.

Hoeben, A., Landuyt, B., Highley, M.S., Wildiers, H., Van Oosterom, A.T., and De Bruijn, E.A. (2004). Vascular endothelial growth factor and angiogenesis. Pharmacol. Rev. *56*, 549–580.

Joaquin, M., and Watson, R.J. (2003). Cell cycle regulation by the B-Myb transcription factor. Cell. Mol. Life Sci. *60*, 2389–2401.

Kastrup, J., Jorgensen, E., Ruck, A., Tagil, K., Glogar, D., Ruzyllo, W., Botker, H.E., Dudek, D., Drvota, V., Hesse, B., et al. (2005). Direct intramyocardial plasmid vascular endothelial growth factor-A165 gene therapy in patients with stable severe angina pectoris a randomized double-blind placebo-controlled study: the Euroinject One trial. J. Am. Coll. Cardiol. *45*, 982–988.

Kolossov, E., Bostani, T., Roell, W., Breitbach, M., Pillekamp, F., Nygren, J.M., Sasse, P., Rubenchik, O., Fries, J.W., Wenzel, D., et al. (2006). Engraftment of engineered ES cell-derived cardiomyocytes but not BM cells restores contractile function to the infarcted myocardium. J. Exp. Med. *203*, 2315–2327.

Kumar, S., and Hedges, S.B. (1998). A molecular timescale for vertebrate evolution. Nature *392*, 917–920.

Laflamme, M.A., Chen, K.Y., Naumova, A.V., Muskheli, V., Fugate, J.A., Dupras, S.K., Reinecke, H., Xu, C., Hassanipour, M., Police, S., et al. (2007). Cardiomyocytes derived from human embryonic stem cells in pro-survival factors enhance function of infarcted rat hearts. Nat. Biotechnol. *25*, 1015–1024.

Lan, F., Lee, A.S., Liang, P., Sanchez-Freire, V., Nguyen, P.K., Wang, L., Han, L., Yen, M., Wang, Y., Sun, N., et al. (2013). Abnormal calcium handling properties underlie familial hypertrophic cardiomyopathy pathology in patient-specific induced pluripotent stem cells. Cell Stem Cell *12*, 101–113.

Lepperhof, V., Polchynski, O., Kruttwig, K., Bruggemann, C., Neef, K., Drey, F., Zheng, Y., Ackermann, J.P., Choi, Y.H., Wunderlich, T.F., et al. (2014). Bioluminescent imaging of genetically selected induced pluripotent stem cell-derived cardiomyocytes after transplantation into infarcted heart of syngeneic recipients. PLoS One *9*, e107363.

Liang, P., Sallam, K., Wu, H., Li, Y., Itzhaki, I., Garg, P., Zhang, Y., Vermglinchan, V., Lan, F., Gu, M., et al. (2016). Patient-specific and genome-edited induced pluripotent stem cell-derived cardiomyocytes elucidate single-cell phenotype of Brugada syndrome. J. Am. Coll. Cardiol. *68*, 2086–2096.

Liao, J., Cui, C., Chen, S., Ren, J., Chen, J., Gao, Y., Li, H., Jia, N., Cheng, L., Xiao, H., et al. (2009). Generation of induced pluripotent stem cell lines from adult rat cells. Cell Stem Cell *4*, 11–15.

Liu, Z., Wen, X., Wang, H., Zhou, J., Zhao, M., Lin, Q., Wang, Y., Li, J., Li, D., Du, Z., et al. (2013). Molecular imaging of induced pluripotent stem cell immunogenicity with in vivo development in ischemic myocardium. PLoS One *8*, e66369.

Mangi, A.A., Noiseux, N., Kong, D., He, H., Rezvani, M., Ingwall, J.S., and Dzau, V.J. (2003). Mesenchymal stem cells modified with Akt prevent remodeling and restore performance of infarcted hearts. Nat. Med. *9*, 1195–1201.

Menasché, P., Vanneaux, V., Fabreguettes, J.R., Bel, A., Tosca, L., Garcia, S., Bellamy, V., Farouz, Y., Pouly, J., Damour, O., et al. (2015). Towards a clinical use of human embryonic stem cell-derived cardiac progenitors: a translational experience. Eur. Heart J. *36*, 743–750.

Menasché, P., Vanneaux, V., Hagège, A., Bel, A., Cholley, B., Parouchev, A., Cacciapuoti, I., Al-Daccak, R., Benhamouda, N., Blons, H., et al. (2018). Transplantation of Human Embryonic Stem Cell–Derived Cardiovascular Progenitors for Severe Ischemic Left Ventricular Dysfunction. JACC *71*, 429–438.

Merkl, C., Saalfrank, A., Riesen, N., Kuhn, R., Pertek, A., Eser, S., Hardt, M.S., Kind, A., Saur, D., Wurst, W., et al. (2013). Efficient generation of rat induced pluripotent stem cells using a non-viral inducible vector. PLoS One *8*, e55170.

Mirotsou, M., Zhang, Z., Deb, A., Zhang, L., Gnecchi, M., Noiseux, N., Mu, H., Pachori, A., and Dzau, V. (2007). Secreted frizzled related protein 2 (Sfrp2) is the key Akt-mesenchymal stem cell-released paracrine factor mediating myocardial survival and repair. Proc. Natl. Acad. Sci. USA *104*, 1643–1648.

Moriya, J., Wu, X., Zavala-Solorio, J., Ross, J., Liang, X.H., and Ferrara, N. (2014). Platelet-derived growth factor C promotes revascularization in ischemic limbs of diabetic mice. J. Vasc. Surg. *59*, 1402–1409.e1-4.

Neofytou, E., O'Brien, C.G., Couture, L.A., and Wu, J.C. (2015). Hurdles to clinical translation of human induced pluripotent stem cells. J. Clin. Invest. *125*, 2551–2557.

Nowsheen, S., and Yang, E.S. (2012). The intersection between DNA damage response and cell death pathways. Exp. Oncol. *34*, 243–254.

Ong, S.G., Huber, B.C., Lee, W.H., Kodo, K., Ebert, A.D., Ma, Y., Nguyen, P.K., Diecke, S., Chen, W.Y., and Wu, J.C. (2015). Microfluidic single-cell analysis of transplanted human induced pluripotent stem cell-derived cardiomyocytes after acute myocardial infarction. Circulation *132*, 762–771.

Park, C.W., Kim, H.W., Lim, J.H., Yoo, K.D., Chung, S., Shin, S.J., Chung, H.W., Lee, S.J., Chae, C.B., Kim, Y.S., et al. (2009). Vascular endothelial growth factor inhibition by dRK6 causes endothelial apoptosis, fibrosis, and inflammation in the heart via the Akt/eNOS axis in Db/Db mice. Diabetes *58*, 2666–2676.

Pfeffer, M.A., and Braunwald, E. (1990). Ventricular remodeling after myocardial infarction. Experimental observations and clinical implications. Circulation *81*, 1161–1172.

Sahn, D.J., DeMaria, A., Kisslo, J., and Weyman, A. (1978). Recommendations regarding quantitation in M-mode echocardiography: results of a survey of echocardiographic measurements. Circulation *58*, 1072–1083.

Shiba, Y., Gomibuchi, T., Seto, T., Wada, Y., Ichimura, H., Tanaka, Y., Ogasawara, T., Okada, K., Shiba, N., Sakamoto, K., et al. (2016). Allogeneic transplantation of iPS cell-derived cardiomyocytes regenerates primate hearts. Nature *538*, 388–391.

Stewart, D.J., Kutryk, M.J., Fitchett, D., Freeman, M., Camack, N., Su, Y., Della Siega, A., Bilodeau, L., Burton, J.R., Proulx, G., et al. (2009). VEGF gene therapy fails to improve perfusion of ischemic myocardium in patients with advanced coronary disease: results of the northern trial. Mol. Ther. *17*, 1109–1115.

Sun, N., Panetta, N.J., Gupta, D.M., Wilson, K.D., Lee, A., Jia, F., Hu, S., Cherry, A.M., Robbins, R.C., Longaker, M.T., et al. (2009). Feeder-free derivation of induced pluripotent stem cells from adult human adipose stem cells. Proc. Natl. Acad. Sci. USA *106*, 15720–15725.

Takenaka-Ninagawa, N., Kawabata, Y., Watanabe, S., Nagata, K., and Torihashi, S. (2014). Generation of rat-induced pluripotent stem cells from a new model of metabolic syndrome. PLoS One *9*, e104462.

Vos, T., Flaxman, A.D., Naghavi, M., Lozano, R., Michaud, C., Ezzati, M., Shibuya, K., Salomon, J.A., Abdalla, S., Aboyans, V., et al. (2012). Years lived with disability (YLDS) for 1160 sequelae of 289 diseases and injuries 1990-2010: a systematic analysis for the global burden of disease study 2010. Lancet *380*, 2163–2196.

Webby, C.J., Wolf, A., Gromak, N., Dreger, M., Kramer, H., Kessler, B., Nielsen, M.L., Schmitz, C., Butler, D.S., Yates, J.R., 3rd., et al. (2009). Jmjd6 catalyses lysyl-hydroxylation of U2AF65, a protein associated with RNA splicing. Science *325*, 90–93.

Wu, S., Zhu, Y., Liu, H., Tang, L., Du, R., Shen, Y., Feng, J., Zhang, K., Xu, C., Zhang, S., et al. (2016). In vivo dynamic metabolic changes after transplantation of induced pluripotent stem cells for ischemic injury. J. Nucl. Med. *57*, 2012–2015.

Xu, X.H., Xu, J., Xue, L., Cao, H.L., Liu, X., and Chen, Y.J. (2011). VEGF attenuates development from cardiac hypertrophy to heart failure after aortic stenosis through mitochondrial mediated apoptosis and cardiomyocyte proliferation. J. Cardiothorac. Surg. *6*, 54.

Yamaguchi, T., Hamanaka, S., and Nakauchi, H. (2014). The generation and maintenance of rat induced pluripotent stem cells. Methods Mol. Biol. *1210*, 143–150.

Ye, J., Gaur, M., Zhang, Y., Sievers, R.E., Woods, B.J., Aurigui, J., Bernstein, H.S., and Yeghiazarians, Y. (2015). Treatment with hESC-derived myocardial precursors improves cardiac function after a myocardial infarction. PLoS One *10*, e0131123.

Ye, L., Chang, Y.H., Xiong, Q., Zhang, P., Zhang, L., Somasundaram, P., Lepley, M., Swingen, C., Su, L., Wendel, J.S., et al. (2014). Cardiac repair in a porcine model of acute myocardial infarction with human induced pluripotent stem cell-derived cardiovascular cells. Cell Stem Cell *15*, 750–761.

Yeghiazarians, Y., Gaur, M., Zhang, Y., Sievers, R.E., Ritner, C., Prasad, M., Boyle, A., and Bernstein, H.S. (2012). Myocardial improvement with human embryonic stem cell-derived cardiomyocytes enriched by p38MAPK inhibition. Cytotherapy *14*, 223–231.

Stem Cell Reports, Volume *10*

Supplemental Information

Comparison of Non-human Primate versus Human Induced Pluripotent Stem Cell-Derived Cardiomyocytes for Treatment of Myocardial Infarction

Xin Zhao, Haodong Chen, Dan Xiao, Huaxiao Yang, Ilanit Itzhaki, Xulei Qin, Tony Chour, Aitor Aguirre, Kim Lehmann, Youngkyun Kim, Praveen Shukla, Alexandra Holmström, Joe Z. Zhang, Yan Zhuge, Babacar C. Ndoye, Mingtao Zhao, Evgenios Neofytou, Wolfram-Hubertus Zimmermann, Mohit Jain, and Joseph C. Wu

STEM-CELL-REPORTS-D-17-00202

Supplemental Materials

Comparison of Non-Human Primate vs. Human Induced Pluripotent Stem Cell-Derived Cardiomyocytes for Treatment of Myocardial Infarction

Xin Zhao[1, 2, 7], Haodong Chen[1, 2, 7], Dan Xiao[1, 2, 7], Huaxiao Yang[1, 2], Ilanit Itzhaki[1, 2], Xulei Qin[1, 2], Tony Chour[1, 2], Aitor Aguirre[3], Kim Lehmann[3], Youngkyun Kim[1, 2], Praveen Shukla[1, 2], Alexandra Holmström[1, 2], Joe Z. Zhang[1, 2], Yan Zhuge[1, 2], Babacar C. Ndoye[1, 2], Mingtao Zhao[1, 2], Evgenios Neofytou[1, 2], Wolfram-Hubertus Zimmermann[4, 5], Mohit Jain[3], Joseph C. Wu[1, 2, 6*]

[1]Stanford Cardiovascular Institute, Stanford, California

[2]Institute for Stem Cell Biology and Regenerative Medicine, Stanford, California

[3]Departments of Medicine and Pharmacology, University of California, San Diego, California

[4]Institute of Pharmacology and Toxicology, University Medical Center, Goettingen, Goettingen, Germany

[5]DZHK (German Center for Cardiovascular Research, partner site Goettingen, Germany

[6]Department of Medicine, Division of Cardiology, Stanford University School of Medicine, Stanford, California

[7]Authors (X.Z., H.C. and D.X.) contributed equally to this study

STEM-CELL-REPORTS-D-17-00202

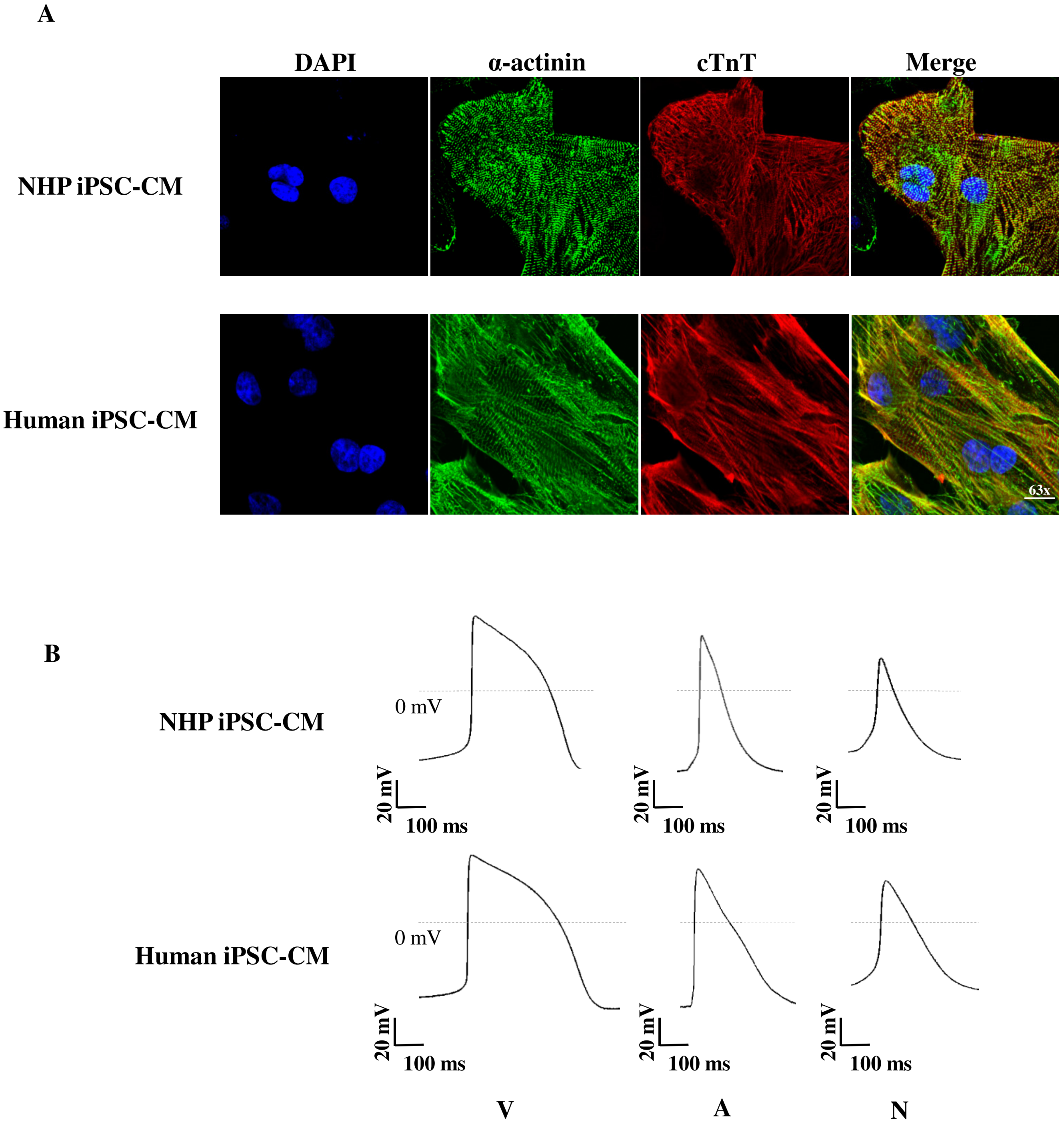

Figure S1. Histological and electrophysiological characterization of NHP iPSC-CMs and human iPSC-CMs. **(A)** Representative iPSC-CM structures stained with α-actinin (green), cardiac troponin T (red), and DAPI (blue). Thirty-day differentiated iPSC-CMs showed well-aligned sarcomere structure. **(B)** Representative recordings of the three major CM action potential (AP) subtypes using whole cell patch clamp. Cells exhibit ventricular-like (V), atrial-like (A), or nodal-like (N) AP morphology.

STEM-CELL-REPORTS-D-17-00202

A Cell Survival

NHP iPSC-CMs

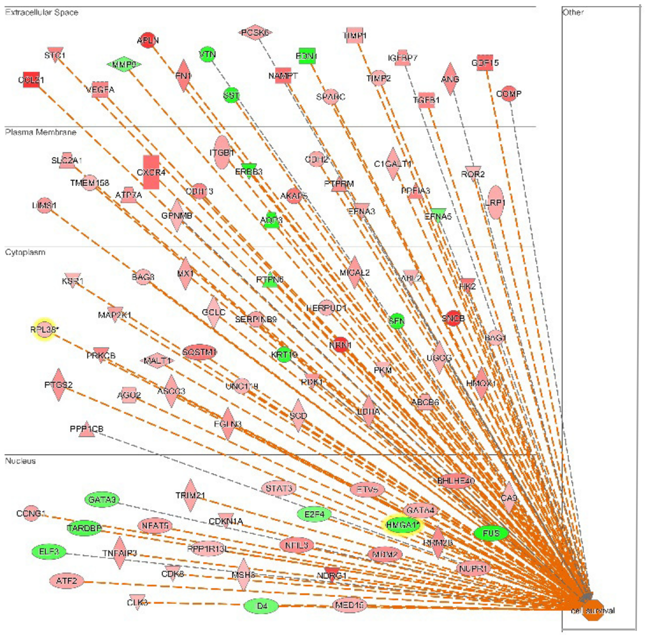

Human iPSC-CMs

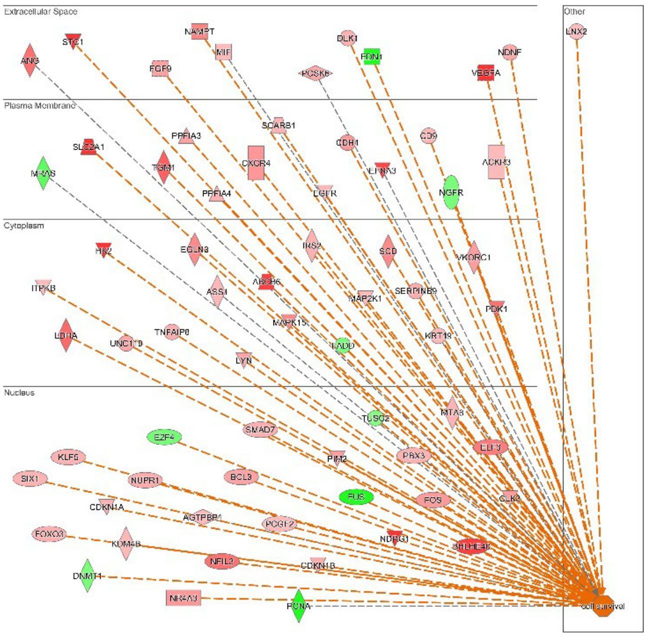

B Angiogenesis

NHP iPSC-CMs

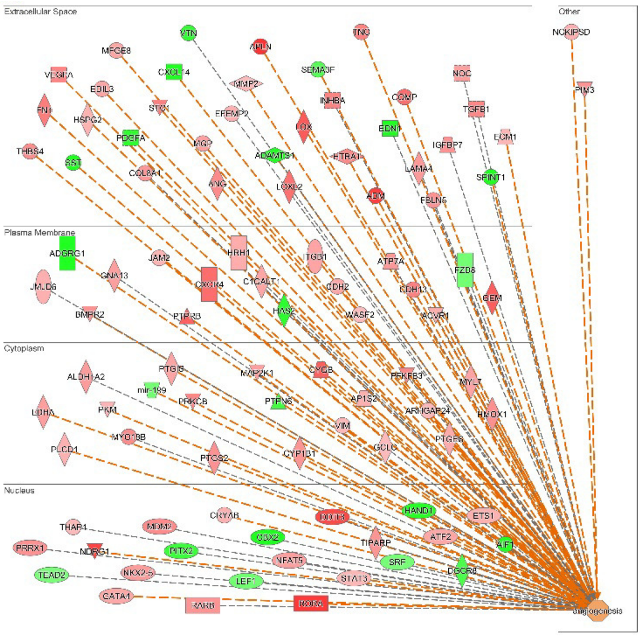

Human iPSC-CMs

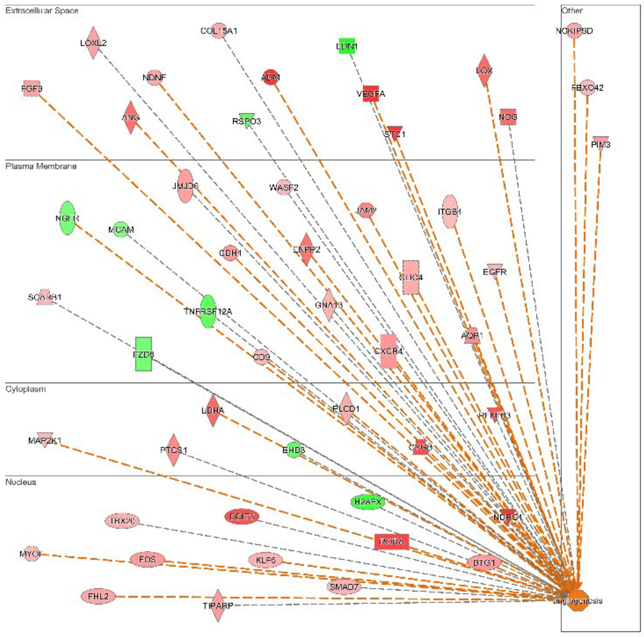

Figure S2. IPA analysis from RNA-seq categorized the significantly regulated genes according to extracellular matrix, membrane, cytosol, and nucleus. Genes in red means upregulation, genes in green means downregulation, and the dash lines in red suggest the gene promotes the pathway. **(A)** Genes responsible for cell survival. **(B)** Genes responsible for angiogenesis.

STEM-CELL-REPORTS-D-17-00202

A Hypertrophy

NHP iPSC-CMs

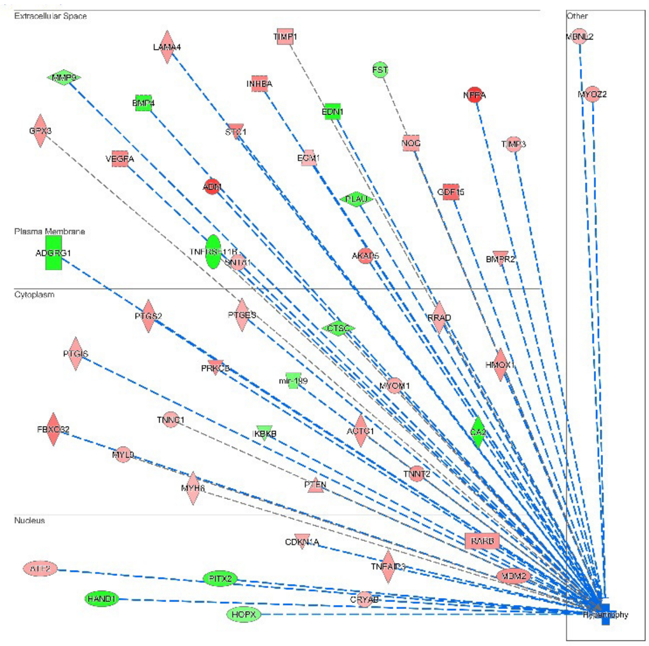

Human iPSC-CMs

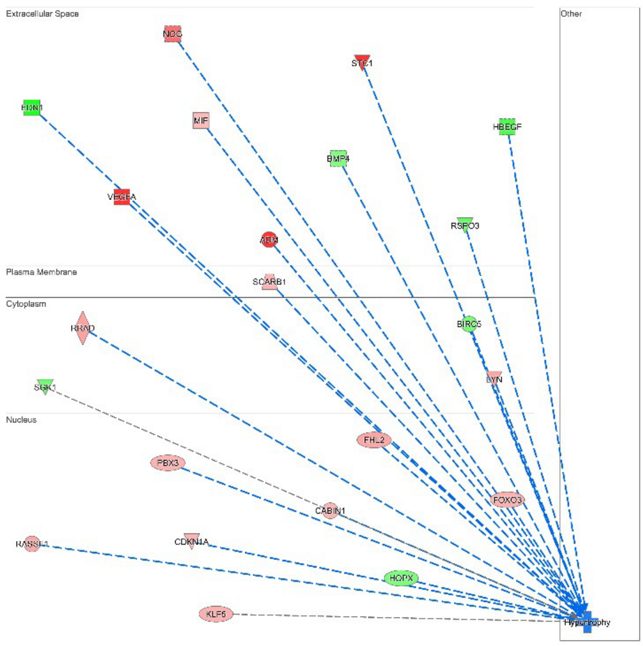

B Fibrosis

NHP iPSC-CMs

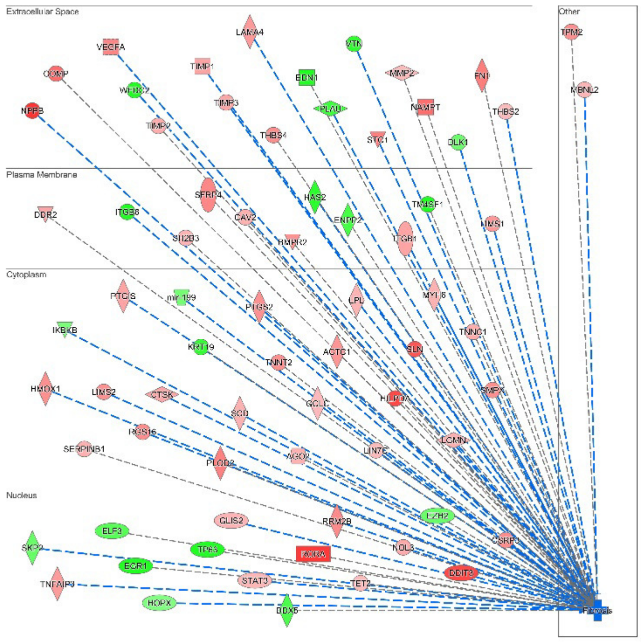

Human iPSC-CMs

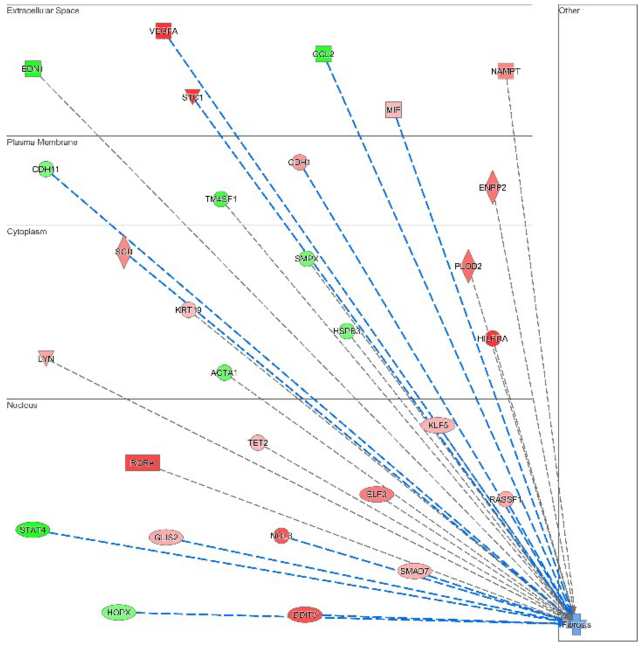

Figure S3. IPA analysis from RNA-seq categorized the significantly regulated genes according to extracellular matrix, membrane, cytosol, and nucleus. Genes in red means upregulation, genes in green means downregulation, and the dash lines in blue suggest the gene inhibits the pathway. **(A)** Genes responsible for hypertrophy. **(B)** Genes responsible for fibrosis.

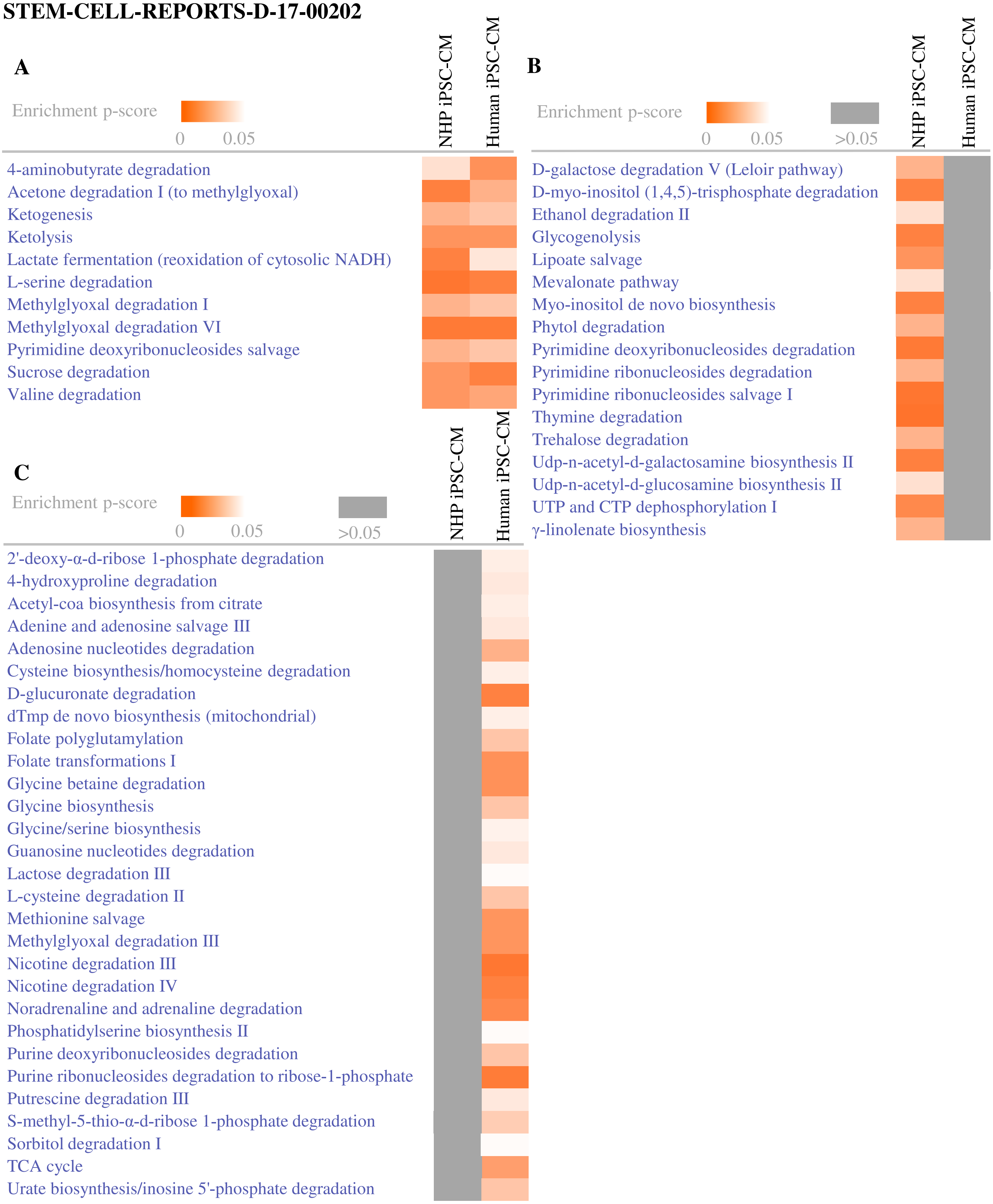

Figure S4. Metabolomics analysis of metabolism related pathways in response to oxygen depletion. **(A)** Common pathways shared between the two species. **(B)** Pathways that are significantly regulated in NHP iPSC-CMs, but not in human iPSC-CMs (grey color). **(C)** Pathways that are significantly regulated in human iPSC-CMs, but not in NHP iPSC-CMs (grey color). N=3.

Table S1: Summary of action potential parameters of NHP and human iPSC-CMs

**NHP iPSC-CMs**	**MDP (mv)**	**APA (mV)**	**Overshoot (mV)**	**Upstroke Velocity (V/sec)**	**APD50 (msec)**	**APD70 (msec)**	**APD90 (msec)**	**Beating rate (bpm)**
**Ventricular-like**	-65 ± 9.2	107 ± 6.0	43 ± 8.6	19 ± 1.3	150 ± 56	174 ± 65	193 ± 71	81 ± 12
**Atrial-like**	-62 ± 4.2	102 ± 7.3	40 ± 7.8	25 ± 15	105 ± 53	130 ± 56	159 ± 58	89 ± 35
**Nodal-like**	-47 ± 3.7	72 ± 2.7	25 ± 6.4	3.4 ± 0.2	71 ± 11	101 ± 25	146 ± 50	134 ± 72
**Human iPSC-CMs**								
**Ventricular-like**	-64 ± 3.9	113 ± 9.1	49 ± 5.8	21 ± 7.7	230 ± 67	264 ± 73	290 ± 78	60 ± 18
**Atrial-like**	-64 ± 4.5	105 ± 7.4	40 ± 9.2	31 ± 20	133 ± 49	169 ± 61	208 ± 72	66 ± 30
**Nodal-like**	-51 ± 2.9	82 ± 10	32 ± 6.9	3.1 ± 1.7	79 ± 26	102 ± 34	130 ± 41	133 ± 28

Results are provided as mean ± SEM. MDP: maximal diastolic potential. APA: action potential amplitude. APD50: action potential duration at 50% repolarization. APD70: action potential duration at 70% repolarization. APD90: action potential duration at 90% repolarization. bpm: beats per minute.

Table S2: Echocardiography assessment before cell injection

	PBS (n=14)	NHP Fibroblast (n=9)	NHP iPSC-CM (n=14)	Human fibroblast (n=9)	Human iPSC-CM (n=14)
LV Ejection Fraction (%)	48.4±1.7	36.0±1.3	48.2±2.9	34.1±3.4	47.9±2.0
LV Fractional Shortening (%)	25.1±1.1	17.9±0.7	25.1±1.8	17.0±1.9	24.9±1.2
LV End-diastolic Volume (µl)	216±13	295±19	193±10	285±17	210±8.2
LV End-systolic Volume (µl)	113±9	190±14	101±9	191±19	110±6.8

Data are presented as Mean ± SE

Table S3: Cardiac function at 4 weeks after cell injection

	**PBS (n=7)**	**NHP Fibroblast (n=9)**	**NHP iPSC-CM (n=11)**	**Human fibroblast (n=9)**	**Human iPSC-CM (n=12)**
Heart Rate (bpm)	356±13	372±14	353±7.9	357±9	345±9.9
LV Systolic Pressure (mmHg)	113±4.3	99±2.0	111±2.4	101±2.4	112±2.6†
Mean Arterial Pressure (mmHg)	105±5.5	91±2.9	98±3.0	90±2.3	100±3.0
LV Maximum dP/dt (mmHg/s)	6628±103	5835±242	7497±316*$^{\#}$	6385±259	7991±434*†
LV Minimum dP/dt (mmHg/s)	-5327±101	-5357±333	-6393±385*$^{\#}$	-5633±196	-6740±499‡
End-diastolic Pressure (mmHg)	8.2±0.9	12±0.8	5.4±0.6**$^{\#}$	8.6±0.7	5.2±0.7†‡
Tau (ms)	11±0.4	12±0.4	9.8±0.4**	12±0.3	9.7±0.3††‡

Data are presented as mean ± SEM; *p<0.05, **p<0.01 NHP iPSC-CM vs. NHP fibroblast; $^{\#}$p<0.05, $^{\#\#}$p<0.01 NHP iPSC-CM vs. PBS; †p<0.05, ††p<0.01 human iPSC-CM vs. human fibroblast; and ‡p<0.05, ‡‡p<0.01 human iPSC-CM vs. PBS.

Table S5: Comparison of transcriptional factors regulated after hypoxia in NHP iPSC-CMs and human iPSC-CMs

NHP iPSC-CM					Human iPSC-CM			
Term	Count	%	Benjamini		Term	Count	%	Benjamini
SRF	319	76.7	2.50E-10		SRF	193	72.6	2.90E-04
MEF2	330	79.3	4.80E-10		MEF2	204	76.7	6.40E-04
SOX5	234	56.2	1.60E-09		SOX5	133	50.0	3.10E-03
RSRFC4	228	54.8	4.90E-09		RSRFC4	134	50.4	1.20E-03
USF	266	63.9	1.30E-07		USF	167	62.8	2.20E-04
TATA	247	59.4	2.10E-07		TATA	147	55.3	3.10E-03
HNF1	252	60.6	2.20E-07		HNF1	160	60.2	2.90E-04
AP1	269	64.7	2.20E-07		AP1	170	63.9	2.40E-04
GATA1	342	82.2	2.20E-07		GATA1	218	82.0	4.30E-04
FOXO4	247	59.4	2.40E-07		FOXO4	150	56.4	1.50E-03
FOXJ2	289	69.5	2.50E-07		FOXJ2	181	68.0	4.30E-04
CDC5	211	50.7	2.70E-07		CDC5	128	48.1	1.30E-03
LMO2COM	262	63.0	4.60E-07		LMO2COM	165	62.0	4.70E-04
CDP	266	63.9	5.10E-07		CDP	166	62.4	8.40E-04
FREAC7	223	53.6	5.10E-07		FREAC7	134	50.4	3.00E-03
POU3F2	262	63.0	5.30E-07		POU3F2	165	62.0	5.00E-04
ARNT	219	52.6	5.50E-07		ARNT	141	53.0	3.20E-04
NKX61	202	48.6	1.20E-06		NKX61	123	46.2	2.40E-03
CEBPB	245	58.9	2.40E-06		CEBPB	156	58.6	5.10E-04
STAT5A	253	60.8	2.60E-06		STAT5A	162	60.9	4.60E-04
FREAC4	209	50.2	4.10E-06		FREAC4	137	51.5	3.00E-04
E2F	248	59.6	8.80E-06		E2F	154	57.9	3.00E-03
CEBP	319	76.7	1.00E-05		CEBP	200	75.2	2.90E-03
HNF3B	187	45.0	1.30E-05		HNF3B	122	45.9	5.20E-04
NMYC	150	36.1	1.60E-05		NMYC	93	35.0	3.10E-03
NFY	214	51.4	2.60E-05		NFY	135	50.8	2.70E-03
HLF	183	44.0	2.60E-05		HLF	123	46.2	2.20E-04
FOXO1	198	47.6	2.80E-07		CREB	129	48.5	2.60E-04
HFH3	195	46.9	3.40E-07		NKX25	179	67.3	3.70E-04
TBP	174	41.8	3.90E-07		BRACH	155	58.3	7.30E-04
SRY	199	47.8	4.30E-07		COUP	122	45.9	9.20E-04
MYCMAX	276	66.3	4.30E-07		ATF6	132	49.6	9.40E-04
AHRARNT	238	57.2	5.70E-07		HTF	150	56.4	1.00E-03
IRF7	207	49.8	2.60E-06		FOXO3	87	32.7	1.00E-03
HFH1	206	49.5	3.00E-06		SEF1	143	53.8	1.20E-03
GATA	204	49.0	4.00E-06		SP1	79	29.7	1.30E-03
GCNF	251	60.3	4.00E-06		FAC1	135	50.8	1.40E-03
STAT1	185	44.5	4.20E-06		AML1	204	76.7	1.50E-03
SOX9	202	48.6	4.50E-06		TAXCREB	150	56.4	1.50E-03
SREBP1	261	62.7	7.80E-06		E4BP4	124	46.6	1.60E-03
FREAC3	195	46.9	7.90E-06		MYOD	154	57.9	1.60E-03
1-Oct	360	86.5	1.30E-05		MZF1	136	51.1	1.80E-03
FREAC2	154	37.0	1.30E-05		P53	176	66.2	2.30E-03
PAX4	322	77.4	1.50E-05		BRN2	139	52.3	2.40E-03
MEIS1AHOXA9	163	39.2	1.70E-05		IK3	125	47.0	2.80E-03
EVI1	335	80.5	1.80E-05		ATF	94	35.3	2.80E-03
BACH2	194	46.6	2.40E-05		PAX6	148	55.6	2.80E-03
MSX1	188	45.2	2.40E-05		NKX3A	129	48.5	2.90E-03
FOXD3	159	38.2	2.60E-05		GATA3	73	27.4	3.00E-03
GFI1	198	47.6	2.80E-05		NFE2	102	38.3	3.90E-03

STEM-CELL-REPORTS-D-17-00202

SUPPLEMENTAL METHODS

Whole-cell patch-clamp recordings. NHP and human iPSC-CM monolayers were enzymatically dispersed (Accutase, Sigma) and attached to Matrigel-coated glass coverslips (Warner, USA). Whole-cell patch clamp recordings were conducted using an EPC-10 patch clamp amplifier (HEKA, Germany). 3-4 MΩ glass pipettes were prepared with a micropipette puller (Sutter Instrument, P-97, USA) using thin-wall borosilicate glass (A-M System, USA). Action potentials (APs) were recorded from iPSC-CMs superfused with Tyrode solution at 37°C (TC-324B heating system, Warner, USA). The Tyrode solution consisted of NaCl (140 mM), KCl (5.4 mM), $CaCl_2$ (1.8 mM), $MgCl_2$ (1 mM), HEPES (10 mM), and glucose (10 mM); pH was adjusted to 7.4 with NaOH. The pipette solution consisted of KCl (120 mM), $MgCl_2$ (1 mM), Mg-ATP (3 mM), HEPES (10 mM), and EGTA (10 mM), pH was adjusted to 7.2 with KOH. Data were acquired using PatchMaster software (HEKA, Germany) and digitized at 1.0 kHz. Data were analyzed using a custom-written MATLAB program.

Culture of NHP and human fibroblasts. NHP and human skin fibroblasts were cultured using FGM™ Fibroblast Growth Media Kits (Lonza, Switzerland). Cells were passaged every 5 days.

Myocardial infarction. Sixty male RNU rats (Charles River Laboratories, Wilmington, MA) weighing between 250-350g, were treated with buprenorphine (0.05 mg/kg s.c.) and cefazolin (50 mg/kg i.m.), and then anesthetized with 2% inhaled isoflurane. Rectal temperature was monitored and body temperature was maintained at 37°C with a heating plate. After intubation of the trachea, rats were ventilated with a tidal volume of 0.5 ml/kg at a rate of 90 breaths per minute. A left thoracotomy was performed at the fourth intercostal space and myocardial ischemia was induced by occlusion of the left anterior descending (LAD) coronary

artery against a segment of PE-200 tubing. Sixty minutes after the ligation, the ligature was released and the chest was closed. Buprenorphine (0.05 mg/kg s.c.), carprofen (5 mg/kg, s.c.), and cefazolin (50 mg/kg i.m.) were given as post-operative medications. All operations were performed by a blinded microsurgeon. Study protocols were approved by the Stanford Animal Research Committee. Animal care was provided in accordance with the Stanford University School of Medicine guidelines and policies for the use of laboratory animals.

P-V loop. Animals were anesthetized with 2% inhaled isoflurane, and a multi-segment 1.4F Millar PV-loop catheter (SPR-838, Millar Instruments, TX) was inserted via the right carotid artery into the LV (Zhao et al. , 2012). Adequate placement of the catheter was verified by the PV-loop signals. A 5-0 silk suture was used for inferior vena cava occlusion (IVCO), followed by the abdominal incision closure followed. Baseline hemodynamics was recorded, followed by IVCO. The linear end-systolic pressure-volume relationship (ESPVR) was obtained from the series of pressure-volume relationship regression curves at decreased preloads.

Tissue preparation for capillary density. To measure vessel density, heparinized animals were euthanized with saturated KCl after being anesthetized with 4% isoflurane. The ascending aorta was dissected and cannulated for perfusion of a vasodilation solution and a fixation solution (Limbourg et al. , 2009). Briefly, 10 ml of 1 x Dulbecco's Phosphate-Buffered Saline (DPBS) with heparin (100 U/ml), 10 ml of vasodilation solution [1 x DPBS with adenosine (100 mM) and sodium nitroprusside (10 mM)], and 10 ml of fixation solution [4% paraformaldehyde (PFA) solution (Thermo Fisher Scientific, CA] were infused consecutively at a constant pressure of 80 mmHg.

Histology staining. Hearts were collected and perfused with 4% PFA. The right ventricle was trimmed and LV was transversely sliced into 6 rings with 1.5-2 mm in thickness. LV

samples were stored in PFA at 4ºC for 24 hr, and transferred into 30% sucrose (Sigma Aldrich, MO) afterwards for another 24 hr. Then the LV rings were embedded individually in cryomold molds with the OCT compound (Tissue-Tek, Fisher Scientific, NH). LV rings were sectioned at 10 µm thickness and assessed for the following:

A) Scar size: LV sections from each ring were stained with Masson's trichrome. The scar size was quantified as the percentage of the area of the fibrotic tissue to the area of each LV ring, then normalized by the weight of each LV section before fixation.

B) Graft staining: Both implanted NHP iPSC-CMs and human iPSC-CMs were identified with antibody against human mitochondria and cardiac troponin T (TnT).

C) Interstitial fibrosis: Tissue sections were stained with Picro-Sirius Red (PSR) to identify fibrosis deposition at both border zone and remote zone. Using ImagePro-Plus software, the percentage of total interstitial fibrosis was quantified at 20x magnification (Peter et al. , 2007).

D) Cell size: The profile of endogenous rat cardiomyocytes was recognized using rhodamine-conjugated wheat germ agglutinin (WGA). The circumference of each cardiomyocyte was traced at 40x magnification and quantified using ImagePro-Plus software.

E) Capillary density: Using CD144 (Cell Signaling, MA), capillaries were identified as a single endothelial cell layer with a diameter less than 25 µm. Capillary density was quantified at 40x magnification as the absolute number per unit myocardial area.

Proteomic VEGF assay. Media collected from both cells in both normoxic and anaerobic conditions were centrifuged at 500g for 3 min. Using the Human Proteomic Angiogenesis Assay, 1 ml supernatant was transferred into 1.5 ml Eppendorf tubes with 0.5 ml Array Buffer 4. Fifteen microliter of reconstituted Detection Antibody Cocktail was added to each sample and incubated for one hour. The samples were then added to a 4-well multi-dish containing blotting membrane,

and kept at 4˚C overnight. Membranes were washed in 20 ml wash buffer for 10 min x 3 times and rinsed with deionized water in between. Afterwards, 2 ml of diluted Streptavidin-HRP with Array Buffer 5 was added into each well of the 4-well multi-dish and incubated for 30 min at room temperature. The membrane was then developed with the Chemi Reagent Mix followed by imaging with the Bio-Rad Chemidoc Imaging system. Using Image J software, the intensity of positive blot representing VEGF was analyzed. The final results of both NHP iPSC-CMs and human iPSC-CMs were calculated and presented as fold-change relative to normoxic condition.

RNA preparation. RNA was extracted using RNeasy Mini Kit (Qiagen, Germany). Briefly, cultured iPSC-CMs were homogenized in Trizol (1 ml / 5 x 10^6 cells, Roche, Switzerland) and chloroform (0.2 ml) was added. After vortexing and incubating at room temperature for 10 min, samples were centrifuged at 12,000 rpm at 4°C for 20 min. The upper aqueous phase containing RNA was collected with equal volume of 70% ethanol added, and the resulting mixture was loaded into an isolation column provided with the kit. After washing several times with wash buffer, RNA was eluted from the column with nuclease free water. RNA quality was tested using NanoDrop (Thermo Scientific, DE) with the criterion of $1.8 < A_{260/280} < 2.0$.

Liquid chromatography-mass spectrometry (LC-MS). Media samples were briefly vortexed and 50 µL of each were taken to a new microfuge tube. 200 µL of cold methanol with an internal standard (^{13}C glutamine, 50 uM concentration in each sample) was added to each tube. Samples were then vortexed for 30 sec and allowed to sit at -80˚C for 30 min to facilitate protein precipitation. Samples were vortexed again for 30 sec, followed by centrifugation at 14,000 rpm for 10 min at 4˚C, after which 50 µL of supernatant was transferred to glass LC-MS vial. For LC-MS, sample volumes were transferred to glass vials and kept at 4˚C in the autosampler compartment until 1 µl of sample was injected for analysis. Compounds were separated using a

Thermo Vanquish UPLC coupled to a Thermo QExactive Orbitrap mass spectrometer. Separation was performed using a Millipore (Sequant) Zic-pHILIC 2.1 × 150 mm 5 µm column maintained at 25˚C using a flow rate of 0.3 mL/min and a 15 min linear gradient starting from 90:10 acetonitrile: 20 mM ammonium bicarbonate, pH 9.6 to 45:55 acetonitrile: 20 mM ammonium bicarbonate, pH 9.6. Detection was performed in positive and negative ion modes through sequential sample injections using a heated electrospray ionization (HESI) source operated at 2.5 kV (negative mode) and 3.5 kV (positive mode), sheath gas flow of 40, auxiliary gas flow of 20, sweep gas flow of 2, capillary temperature of 275˚C, and auxiliary gas heater temperature of 350˚C. Data were collected using data-dependent tandem MS collection with MS1 parameters of 70,000 mass resolution, 100 ms maximum IT time, 3×10^6 AGC volume, a mass range of 67 to 1000 m/z, MS2 parameters of 17,500 mass resolution, 50 ms maximum IT time, 1×10^5 AGC volume, loop count of 5, isolation window of 0.5 m/z, NCE of 35, and a 10-second dynamic exclusion. When possible, LC-MS peaks were assigned metabolite identity by matching accurate mass and retention time against pure standards. Data extraction and analysis were performed using Mzmine, XCMS, and a combination of in-house developed tools. Metabolite pathway enrichment was obtained from Metlin libraries.

STEM-CELL-REPORTS-D-17-00202

Reference

Limbourg, A., Korff T., Napp L.C., Schaper W., Drexler H., Limbourg F.P. (2009). Evaluation of Postnatal Arteriogenesis and Angiogenesis in a Mouse Model of Hind-Limb Ischemia. Nat Protoc *4*, 1737-1746.

Peter, P.S., Brady J.E., Yan L., Chen W., Engelhardt S., Wang Y., Sadoshima J., Vatner S.F., Vatner D.E. (2007). Inhibition of P38 Alpha Mapk Rescues Cardiomyopathy Induced by Overexpressed Beta 2-Adrenergic Receptor, but Not Beta 1-Adrenergic Receptor. J Clin Invest *117*, 1335-1343.

Zhao, X., Park J., Ho D., Gao S., Yan L., Ge H., Iismaa S., Lin L., Tian B., Vatner D.E.*, et al.* (2012). Cardiomyocyte Overexpression of the Alpha1a-Adrenergic Receptor in the Rat Phenocopies Second but Not First Window Preconditioning. Am J Physiol Heart Circ Physiol *302*, H1614-1624.
